# Sex differences in endocannabinoids during 3 years of Mediterranean diet intervention: Association with insulin resistance and weight loss in a population with metabolic syndrome

**DOI:** 10.3389/fnut.2022.1076677

**Published:** 2022-12-01

**Authors:** Natalia Soldevila-Domenech, Antoni Pastor, Aleix Sala-Vila, Iolanda Lázaro, Anna Boronat, Daniel Muñoz, Olga Castañer, Beatriz Fagundo, Dolores Corella, Fernando Fernández-Aranda, Miguel Ángel Martínez-González, Jordi Salas-Salvadó, Montserrat Fitó, Rafael de la Torre

**Affiliations:** ^1^Integrative Pharmacology and Systems Neurosciences Research Group, Neuroscience Research Program, Hospital del Mar Medical Research Institute (IMIM), Barcelona, Spain; ^2^Department of Medicine and Life Sciences, Universitat Pompeu Fabra, Barcelona, Spain; ^3^CIBER Fisiopatología Obesidad y Nutrición, Instituto de Salud Carlos III, Madrid, Spain; ^4^Cardiovascular Risk and Nutrition Research Group, Epidemiology and Public Health Program, Hospital del Mar Medical Research Institute (IMIM), Barcelona, Spain; ^5^Fatty Acid Research Institute, Sioux Falls, SD, United States; ^6^Endocrinology Service, Hospital del Mar, Barcelona, Spain; ^7^Department of Physiotherapy, Fundació Universitària del Bages, Manresa, Spain; ^8^Department of Preventive Medicine and Public Health, School of Medicine, University of Valencia, Valencia, Spain; ^9^Department of Psychiatry, University Hospital of Bellvitge-IDIBELL, Barcelona, Spain; ^10^Department of Clinical Sciences, School of Medicine and Health Sciences, University of Barcelona, Barcelona, Spain; ^11^Psychoneurobiology of Eating and Addictive Behaviours Group, Neuroscience Program, Institut d’Investigació Biomèdica de Bellvitge-IDIBELL, L’Hospitalet de Llobregat, Barcelona, Spain; ^12^Department of Preventive Medicine and Public Health, University of Navarra, Pamplona, Spain; ^13^Navarra’s Health Research Institute (IdiSNA), Pamplona, Spain; ^14^Department of Nutrition, Harvard T.H. Chan School of Public Health, Boston, MA, United States; ^15^Universitat Rovira i Virgili, Departament de Bioquímica i Biotecnologia, Unitat de Nutrició Humana, Reus, Spain; ^16^Institut d’Investigació Sanitària Pere Virgili, Hospital Universitari Sant Joan de Reus, Reus, Spain

**Keywords:** sex differences, Mediterranean diet, endocannabinoids, metabolic syndrome, weight loss, insulin resistance, anandamide (AEA), 2-arachidonoylglycerol (2-AG)

## Abstract

**Background:**

Excess circulating endocannabinoids (eCBs) and imbalanced *N*-acylethanolamines (NAEs) related eCBs abundance could influence dietary weight loss success. We aimed to examine sex differences in the impact of a 3-years Mediterranean diet (MedDiet) intervention on circulating eCBs, NAEs and their precursor fatty acids, and to analyze the interplay between changes in eCBs or NAEs ratios, insulin resistance and the achievement of clinically meaningful weight reductions.

**Methods:**

Prospective cohort study in a subsample of *N* = 105 participants (54.3% women; 65.6 ± 4.6 years) with overweight or obesity and metabolic syndrome that underwent a 3-years MedDiet intervention (PREDIMED-Plus study). Plasma eCBs and NAEs, including 2-arachidonoylglycerol (2-AG), anandamide (AEA), oleoylethanolamide (OEA) and palmitoylethanolamide (PEA), fatty acids, diet, glycemic homeostasis (including the assessment of insulin resistance-HOMA-IR), and cardiovascular risk markers were monitored (at 0-6-12-36 months).

**Results:**

Mediterranean diet adherence increased in both sexes and remained high during the 3 years of follow-up. Reductions in body weight, glycemic and cardiovascular parameters were larger in men than in women. Women presented higher concentrations of NAEs than men throughout the study. In both sexes, AEA and other NAEs (including OEA, and PEA) decreased after 6 months (for AEA: −4.9%), whereas the ratio OEA/AEA increased after 1 year (+5.8%). Changes in 2-AG (−3.9%) and the ratio OEA/PEA (+8.2%) persisted over the 3 years of follow-up. In women, 6-months changes in AEA (OR = 0.65) and the ratio OEA/AEA (OR = 3.28) were associated with the achievement of 8% weight reductions and correlated with HOMA-IR changes (*r* = 0.29 and *r* = −0.34). In men, OEA/PEA changes were associated with 8% weight reductions (OR = 2.62) and correlated with HOMA-IR changes (*r* = −0.32).

**Conclusion:**

A 3-years MedDiet intervention modulated plasma concentrations of eCBs and NAEs. Changes in AEA and in the relative abundance of NAEs were associated with clinically meaningful weight reductions. However, marked sex differences were identified in eCBs and NAEs, as well as in the efficacy of the intervention in terms of glycemic and cardiovascular parameters, which could be related to post-menopause alterations in glucose metabolism. These findings support a sex-balanced research strategy for a better understanding of the mechanisms underlying the regulation of body weight loss.

## Introduction

Obesity is a complex multifactorial disease defined by excessive adiposity that currently affects nearly two thirds of adults (1, 2). The obesity pandemic is a serious public health challenge globally as it acts as a gateway to a range of other non-communicable diseases, including cardiovascular diseases, type 2 diabetes, several types of cancers, non-alcoholic fatty liver disease and Alzheimer’s disease (1). Lifestyle modifications, including dietary intervention, is the first step in weight management (3). Accordingly, adherence to an hypocaloric Mediterranean diet (MedDiet) has been associated with weight loss benefits (4) and with multiple health-promoting effects (5). However, weight loss maintenance is difficult to achieve for the majority of people with obesity (6, 7). A better understanding of the biological mechanisms that control appetite and energy utilization, considering sexual dimorphism in energy and substrate metabolism, is needed to design more effective interventions for the prevention and management of obesity-related metabolic diseases (7–9).

The endogenous cannabinoid system, (that is, the endocannabinoid system), is an ubiquitous lipid signaling system that has a critical role in obesity and metabolic syndrome (10). Endocannabinoids (eCBs), the lipid messengers of this neuromodulatory system, are produced *on-demand* by cell membrane phospholipids in the brain, where they stimulate hunger by modulation of synaptic transmission (11, 12), but also in peripheral tissues, including adipose tissue, liver, pancreas, skeletal muscle and gut, where they control lipid and glucose metabolism (13–15). Accordingly, elevated circulating eCBs increase food consumption and suppress orexigenic signals, promote lipogenesis and decrease satiety signals (16). In turn, insulin release decreases plasma concentrations of eCBs (17, 18). Consequently, dyslipidemia, leptin and insulin resistance have been proposed as the mechanisms underlying the hyperactive eCB system in obesity (19, 20).

The two most studied eCBs are *N-*arachidonoyl-ethanolamine (anandamide, AEA) and 2-arachidonoylglycerol (2-AG) (16). Both derive from arachidonic acid (ARA), although they differ in the routes of biosynthesis and degradation (21). eCBs exert their biological actions by binding to the cannabinoid receptors CB1 and CB2, and also to non-cannabinoid receptors such as PPARα and TPRV (in the case of AEA) or PPARγ (in the case of both AEA and 2-AG) (22). Moreover, circulating concentrations of 2-AG and AEA are influenced by the age, body mass index (BMI), visceral fat and metabolic factors (e.g., menopause, diabetes) (23–26). In addition to eCBs, other lipid-related compounds, called eCB-like or cannabimimetic compounds, are included as part of the eCB system although they do not bind to CB1 or CB2 receptors (22, 27). The most known are *N-*acylethanolamines (NAEs), that include compounds such as oleoylethanolamide (OEA), palmitoylethanolamide (PEA) and *N-*docosahexaenoylethanolamine (DHEA), for which oleic acid, palmitic acid and docosahexaenoic acid (DHA) serve as their respective precursor fatty acids (13, 16). All NAEs, including AEA, are biosynthesized and degraded by the same enzymes, which explains the high correlation observed between them (28). However, in contrast to AEA, OEA decreases appetite (29) and DHEA and PEA have anti-inflammatory properties (30). They exert their functions by binding to PPARα, GPR119 or GPR110 receptors (31, 32). In recent studies, the relative abundance of these NAEs expressed in the form of ratios (e.g., OEA/AEA) has been proposed to improve the understanding of the regulation of the endocannabinoid system (23, 33).

Fatty acid composition in the diet could therefore modulate the tissue concentrations of eCBs and NAEs (13, 22, 34), especially in the context of a MedDiet, as it is high in unsaturated fats (about 35–40% of the total energy), mostly from monounsaturated fatty acids (MUFAs, mainly from olive oil) and polyunsaturated fatty acids (PUFAs, mainly from nuts and fish) (35–38). Accordingly, results from a recent randomized clinical trial (RCT) showed that a 8-weeks intervention with MedDiet lowered plasma AEA concentrations and increased the ratios OEA/AEA and OEA/PEA in overweight and obese subjects (33). However, there is a lack of knowledge about the long-term influence of a MedDiet intervention in the endocannabinoid system and its role on weight loss maintenance.

This study aims to analyze sex differences in the interplay between eCBs and NAEs, weight reductions and insulin resistance, during 3 years of MedDiet intervention in a population of older adults with overweight or obesity and metabolic syndrome. The present report has three specific objectives. First, to investigate the presence of sex differences in the impact of a long-term MedDiet intervention on circulating eCBs, NAEs, and their precursor fatty acids. Second, to analyze whether the inter-dependencies between eCBs, precursor fatty acids, glycemic and cardiovascular risk factors and the intake of lipid-rich foods differ by sex. Third, to study the association between eCBs and NAEs ratios and the achievement of a successful weight reduction of at least 8% of body weight after 6 months or 5% of body weight after 3 years of a MedDiet intervention in men and women, and its relationship with insulin resistance.

## Materials and methods

### Study design and population

Prospective cohort study in a subsample of 105 participants from the PREDIMED-Plus study who were recruited at the IMIM-Hospital del Mar study site (Barcelona, Spain). Details on the study design and procedures of the PREDIMED-Plus, as well as the inclusion/exclusion criteria have been already published (4, 39, 40), and the study protocol is available at http://predimedplus.com/. In short, inclusion criteria for the PREDIMED-Plus included community-dwelling overweight or obese subjects (BMI between 27 and 40 kg/m^2^), aged between 55 and 75 years in the case of men and between 60 and 75 years in the case of women, who met at least three criteria of metabolic syndrome (41). The PREDIMED-Plus is one of the largest trials in nutrition ever undertaken in Spain. It is a multi-center randomized parallel-group primary prevention trial that aims to evaluate the long-term effectiveness of a lifestyle intervention with an energy-reduced MedDiet (er-MedDiet, involving 30% calorie reduction), physical activity promotion and behavioral support of weight loss goals (intervention group), compared to a more traditional MedDiet intervention without energy reduction and weight loss goals (active control group), on: (i) the incidence of cardiovascular events (non-fatal myocardial infarction, non-fatal stroke, or cardiovascular death) and (ii) weight loss and long-term maintenance of weight-loss. To promote MedDiet adherence all participants received an allotment of extra-virgin olive oil (1 L/month) and occasionally almonds (125 g/month). In the present study, participants allocated to the intervention or control groups were pooled together and analyzed as a single group, as both were exposed to a MedDiet intervention and showed minimal differences in the modulation of eCBs over the 3 years of follow-up (analyses included in the Supplementary material and available upon request).

The PREDIMED-Plus trial is registered at the International Standard Randomized Controlled Trial database (ISRCTN; 89898870).

### Variables

All variables included in the present study were measured four times: at baseline and after 6 months, 1 year, and 3 years.

#### Anthropometric measurement and biochemical analysis

Weight, height, hip, and waist circumference were measured by nurses with standardized procedures. For descriptive purposes, body mass index (BMI) was also categorized using general population cut-off values based on morbidity and mortality studies of Caucasian population (42): normo-weight (BMI 18.5–24.9 kg/m^2^), overweight (BMI 25.0–29.9 kg/m^2^), obesity I (BMI 30.0–34.9 kg/m^2^) and obesity II (BMI 35.0–39.9 kg/m^2^). Blood pressure was measured in triplicate using a validated semiautomatic oscillometer (Omron HEM 297 705C). Blood samples were collected after an overnight fast to determine lipid concentrations [triglycerides, total cholesterol and high-density lipoprotein cholesterol (HDL-c)] and glycemic control [glycosylated hemoglobin (HbA1c), glucose and insulin], using standard methodology. Low-density lipoprotein cholesterol (LDLc) concentrations were calculated with the Friedewald’s formula whenever triglycerides were inferior to 300 mg/dL.

Baseline type 2 diabetes was defined by previous clinical diagnosis of diabetes or HbA1c ≥ 6.5% or use of anti-diabetic medication or use of insulin or fasting plasma glucose >126 mg/dL. Participants without a diagnosis of diabetes were diagnosed with prediabetes if their fasting plasma glucose concentrations were between 100 and 125 mg/dL at both the screening and baseline visits, and their HbA1c concentrations were between 5.7 and 6.4%. Anti-diabetic medication included insulin, metformin and other oral hypoglycemic drugs (dipeptidyl peptidase 4 inhibitors, sulfonylureas, insulin secretagogues, sodium-glucose cotransporter-2 (SGLT2) inhibitors or thiazolidinediones). Insulin resistance was estimated using the homeostasis model assessment of insulin resistance (HOMA-IR) index with the following formula: $H\,O\,M\,A-I\,R=\frac{\left(f\,a\,s\,t\,i\,n\,g\,i\,n\,s\,u\,l\,i\,n\,i\,n\,\mu \,I\,U/m\,L\right)\times \left(f\,a\,s\,t\,i\,n\,g\,g\,l\,u\,c\,o\,s\,e\,i\,n\,m\,g/d\,L\right)}{405}$ (43).

Finally, demonstration of a clinically significant weight loss of at least 8% of baseline weight after 6 months and of at least 5% of baseline body weight after 3 years were selected as primary efficacy criterion. The 8% cut-off is the specific weight loss objective of the PREDIMED-Plus study (4). Moreover, the European Medicines Agency (EMA) states that a weight loss of at least 5% after 12 months is a valid primary efficacy criterion as relevant improvements in risk factors associated with overweight or obesity have been observed with weight loss of 5 to 10% of initial weight (44).

#### Intervention adherence and food consumption

Adherence to the er-MedDiet was evaluated by trained dietitians with a 17-item er-Mediterranean Adherence Screener (MEDAS) questionnaire (45). Values ranged 0–17 and higher values indicated greater adherence. Scores were also categorized as low (0–7 points), moderate (8–10 points) and high (11–17 points) adherence (46). Intakes of food groups (extra virgin olive oil (EVOO), total olive oil, walnuts, nuts, fatty fish, total fish or seafood, meat products and dairy products), energy (kcal/day) and specific nutrients (total fat, trans fatty acids, total PUFA, total n3-PUFA, n3-PUFA from fatty fish (eicosapentaenoic [EPA] + DHA), MUFA, and saturated fatty acids-SAFA) were quantified in g/day using the Spanish version of a validated 143-item semiquantitative food-frequency questionnaire (FFQ) (47, 48). On the other hand, leisure-time physical activity measured as metabolic equivalent tasks (METs-minute/week) was evaluated with the Minnesota REGICOR Short Physical Activity questionnaire (VREM) (49).

#### Fatty acid determination in blood erythrocytes

Fatty acids were determined in erythrocytes rather than whole plasma or serum because, given the lifespan of erythrocytes (around 120 days), this matrix better reflects the long-term intake of fatty acids (50). Whole blood was extracted from volunteers in the morning after an overnight fast and collected in 10 mL K_2_EDTA Vacutainer tubes. After extraction the tube was homogenized ten times by inversion, aliquoted and stored at −8°C. For the analysis of fatty acids, forty microliters of whole blood were spiked with 10 μg of the internal standard (ISTD) 1,2-dinonadecanoyl-sn-glycero-3-phosphocholine (Avanti, Merck), hemolyzed with distilled water and centrifuged. The supernatant (containing hemoglobin and serum lipids) was discarded, and the pellet (consisting of >99% of red blood cell membranes) was directly trans-esterified using acidified methanol to prepare fatty acid methyl esters (FAMEs), as previously described (51). FAMEs were analyzed by gas chromatography/electron ionization mass spectrometry (GC/MSEI), using an Agilent 6890N GC equipped with an Agilent 7683 autosampler, and an Agilent 5973N mass spectrometry detector. FAMEs were separated with a J&W DB-Fast FAME capillary column (30 m × 0.2 mm × 0.25 μm film thickness, Agilent). The injector temperature was set at 250°C, and 1 μL injections were performed (split ratio 25:1). GC was run using an optimized temperature program, as follows: the program started at 50°C, held for 0.5 min, increased to 194°C at a rate of 25°C/min, held for 1 min, increased to 245°C at a rate of 5°C/min, and held for 3 min. Helium was used as a carrier gas (14 psi, constant pressure mode). FAMEs were detected using the selected ion monitoring (SIM) mode. Several m/z ions common to saturated, monounsaturated, and polyunsaturated FAMEs were monitored (52). Twelve mixtures of FAME external calibration standards, spiked with C19:0-methyl ester in an equivalent amount to that included in samples as phospholipid, were prepared by diluting FAME mix certified reference material (Supelco 37 Component FAME Mix, Merck) in hexane. The concentration of FAMEs in the samples were calculated by linear regression of the peak area ratio relative to that of the internal standard.

The following 20 fatty acids were quantified and are named as symbols or common names: C12:0 (lauric acid), C14:0 (myristic acid), C15:0 (pentadecylic acid), C16:0 (palmitic acid), C16:1n-7 (palmitoleic acid), C17:0 (margaric acid), C18:0 (stearic acid), C18:1n-9 (oleic acid), C18:2n-6 (linoleic acid, LA), C18:3n-3 (alpha-linolenic acid, ALA), C20:0 (arachidic acid), C20:1n-9 (*cis*-11-eicosenoic acid), C20:2n-6 (all *cis*-11,14-eicosadienoic acid), C20:3n-6 (dihomo-gamma-linolenic acid, DHGLA), C20:4n-6 (arachidonic acid, ARA), C20:5n-3 (eicosapentaenoic acid, EPA), C22:0 (behenic acid), C22:1n-9 (erucic acid), C22:6n-3 (docosahexaenoic acid, DHA) and C24:0 (lignoceric acid). The amount of each fatty acid is expressed as a concentration (μM).

#### Endocannabinoids quantification in plasma

Blood extracted from volunteers was collected in the morning after an overnight fast in 10 mL Tubes K2E (EDTA) 18.0 mg (BD Vacutainer tubes) and centrifuged within 10 min at 1,700 *g* in a refrigerated centrifuge (4°C) during 15 min. For the stabilization of the samples, one milliliter of plasma for eCB analysis was separated immediately, spiked with 8 μL of a 250 μg/mL orlistat ethanol solution and stored at −80°C until eCB analysis. Orlistat was used to minimize *ex-vivo* production of 2-AG from plasma (53). eCBs and related compounds were extracted from plasma with tert-butyl-methyl-ether and analyzed by LC-MS/MS in an Acquity UPLC coupled to a Xevo TQ-S Micro triple quadrupole mass spectrometer (Waters Associates, Milford, MA, USA) following a previously validated method (53). Quantification was done by isotopic dilution with the response of isotopically labeled internal standards (2-AG-d5, AEA-d4, DHEA-d4, LEA-d4, OEA-d4, PEA-d4, SEA-3). Reagents were from Merck (Darmstadt, Germany) and standards were obtained from Cayman Chemical (Ann Arbor, Michigan) and Toronto Research Chemicals (North Ontario, Canada). The following compounds were quantified: 2-AG, AEA, *N-*dihomo-γ-linolenoyl ethanolamide (DGLEA), *N-*docosatetraenoylethanolamine (DEA), DHEA, *N-*linoleoylethanolamine (LEA), OEA, *N*-palmitoleoylethanolamine (POEA), PEA, and *N-*stearoylethanolamine (SEA). 2-AG concentrations are reported as the sum of the two isomers 2-AG and 1-AG. The ratios between OEA/AEA, PEA/AEA, DHEA/AEA and OEA/PEA were also calculated. The amount of each endocannabinoid is expressed as a concentration (nM).

The following ratios between eCBs (in nM) and their precursor fatty acid (in μM) were calculated and multiplied by 1000 (expressed in ‰): 2-AG/C20:4n-6 (i.e., 2-AG/ARA), AEA/C20:4n-6 (i.e., AEA/ARA), DGLEA/C20:3n-6 (i.e., DGLEA/DHGLA), DHEA/C22:6n-3 (i.e., DHA/DHA), LEA/C18:2n-6 (i.e., LEA/LA), OEA/C18:1n-9 (i.e., OEA/oleic acid), PEA/C16:0 (i.e., PEA/palmitic acid), POEA/C16:1n-7 (i.e., POEA/palmitoleic acid) and SEA/C18:0 (SEA/stearic acid).

### Statistical analyses

All the analyses were performed in the whole population and by sex, although a description of eCBs and fatty acids stratified by intervention/control groups is included in Supplementary material. Descriptive results in the whole population and by intervention/control groups are presented as figures but tables are available upon request.

First, descriptive statistics of study variables at each time point (baseline, 6 months, 1 year, and 3 years) were obtained as mean ± standard deviation (SD) and median ± interquartile range [quartile 1 (Q1), quartile 3 (Q3)]. Changes from baseline were computed as absolute changes (mean ± SD) and also as relative changes in the case of eCBs (average percentual change from baseline ± SD). Additionally, standardized mean differences between sexes at each time point and in change over time from baseline were computed as Cohen’s d, with cut-offs for effect size interpretation of 0.2 (small), 0.5 (medium), 0.8 (large) and 1.2 (very large) (54, 55). Departures from normality were detected with the Shapiro–Wilk’s test. Accordingly, the 76% of variables (including dietary, anthropometric, glycemic and cardiovascular factors, eCBs, and fatty acids) did not follow a normal distribution at baseline. Consequently, all dependent variables were normalized before estimating linear models using Ordered Quantile (ORQ) transformation, which is effective in producing a successful normalizing transformation regardless of the underlying distribution (56).

Univariate differences between sexes were tested using chi-squared test for categorical variables and Student’s *t*-test for continuous variables. Multivariable-adjusted differences at each time point were estimated with linear models, including sex as explanatory variable, and adjusting by intervention group, age, and diagnostic of diabetes. Mean changes from baseline were analyzed using linear mixed effects models, with participant included as random effects and with time as explanatory variable, and adjusting by the previous covariates. Sex differences in the mean rate of change over time were analyzed by including the interaction between time and sex in such models.

Then, an exploratory univariate correlation analysis among metabolic syndrome characteristics, fat-rich foods, fatty acids and eCBs was performed using the Pearson’s correlation coefficient after normalizing variables. Results are presented in the form of a correlation matrix. In turn, the Spearman’s correlation coefficient was used to explore the relationship between change in eCBs or NAEs, and change in their precursor fatty acids or insulin resistance after 6 months without normalizing variables, in order to keep the percentage change as the unit of measurement.

Finally, subjects were split in two groups of short-term responders and non-responders following the achievement of 8% weight reduction after 6 months, and long-term respondents following the achievement of 5% weight reduction after 3 years. Multivariable-adjusted logistic regression models were used to examine the associations between the percentual change in eCBs and NAEs ratios after 6 months and 3 years, and the probability of achieving these weight reduction goals at those time points. Such models were performed separately in men and women and were adjusted by baseline body weight and by age, intervention group and type 2 diabetes. Potential nonlinear relations were examined using generalized additive models (GAM) with penalized regression splines and automatic smoothness estimation (57).

The rates of missing data are reported for each variable at each time point. The proportion of missing values for eCBs, NAEs and fatty acids is 0% at baseline, 2.9% at 6 months, 11.4% at 1 year and 18.1% at 3 years. Regarding cardiovascular risk factors and dietary intakes, the percentage of missing values is 0% at both baseline and 6 months, 0–5.7% at 1 year, and 3.8–15.2% at 3 years. There were not drop-outs in the study, so missing was assumed to be completely at random (MCAR) and each specific analysis was performed on individuals with complete information on the variables involved. All the analyses were performed with R statistical software, version 4.1.2.

## Results

**FIGURE 1:**
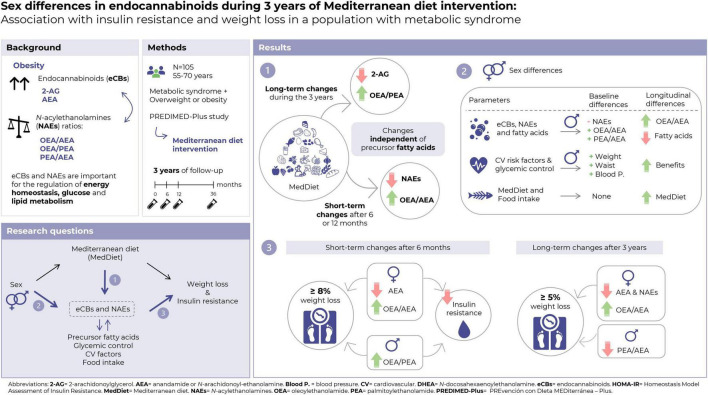
Summary of the main results of the present study.

The main results of the present study are summarized in Figure 1.

### Baseline characteristics of the study population

A total of 105 individuals (57 women and 48 men) were included in the present study (Table 1). Participants were aged 65.6 ± 4.6 years, most of them were retired and almost half presented type 2 diabetes (52.1% in men and 38.6% in women). At baseline, women presented higher MedDiet adherence than men (7.8 ± 2.4 points vs. 6.8 ± 2.7 points; *P* = 0.034), and globally only 11.4% of participants were highly adhered to the MedDiet (er-MEDAS score higher than 11). As part of the inclusion criteria, 83.8% of participants had obesity and the remaining 16.2% presented overweight.

**TABLE 1:** Characteristics of the study population and univariate differences between sexes.

		All	Men	Women	*P*-value
Variable	Category	*N* (%)	*N* (%)	*N* (%)	
*N*		105 (100)	48 (100)	57 (100)	
**Baseline characteristics**					
Study group	Intervention	52 (49.5)	25 (52.1)	27 (47.4)	0.775
Age	Mean (SD)	65.6 (4.6)	65.2 (5.3)	66.0 (3.8)	0.357
Education (years)	Mean (SD)	11.5 (4.1)	13.1 (4.4)	10.2 (3.3)	<**0.001**
Education level	Primary	36 (34.3)	11 (22.9)	25 (43.9)	0.087
	Secondary	41 (39.0)	20 (41.7)	21 (36.8)	
	University (grade)	10 (9.5)	7 (14.6)	3 (5.3)	
	University (higher)	18 (17.1)	10 (20.8)	8 (14.0)	
Employment status	Employed	22 (21.0)	16 (33.3)	6 (10.5)	**0.007**
	Unemployed	5 (4.8)	1 (2.1)	4 (7.0)	
	Housework	3 (2.9)	0 (0.0)	3 (5.3)	
	Retired	75 (71.4)	31 (64.6)	44 (77.2)	
Civil status	Married	82 (78.1)	41 (85.4)	41 (71.9)	0.058
	Single	13 (12.4)	6 (12.5)	7 (12.3)	
	Widowed	10 (9.5)	1 (2.1)	9 (15.8)	
Smoking status	Never smoker	56 (53.3)	17 (35.4)	39 (68.4)	**0.003**
	Former smoker	38 (36.2)	23 (47.9)	15 (26.3)	
	Smoker	11 (10.5)	8 (16.7)	3 (5.26)	
Diabetes status	Prediabetes	14 (13.3)	3 (6.3)	11 (19.3)	0.112
	Diabetes	47 (44.8)	25 (52.1)	22 (38.6)	
Er-MedDiet adherence	Low	55 (52.4)	30 (62.5)	25 (43.9)	0.147
	Moderate	38 (36.2)	13 (27.1)	25 (43.9)	
	High	12 (11.4)	5 (10.4)	7 (12.3)	
Er-MedDiet score	Mean (SD)	7.4 (2.6)	6.8 (2.7)	7.8 (2.4)	**0.034**
**Use of medication**					
Medication for high cholesterol		39 (37.1)	18 (37.5)	21 (36.8)	0.999
Use of tranquilizers/sedatives		25 (23.8)	7 (14.6)	18 (31.6)	0.071
Anti-diabetic medication	All types	40 (38.1)	20 (41.7)	20 (35.1)	0.624
	Insulin	4 (3.81)	1 (2.08)	3 (5.26)	0.623
	Metformin	37 (35.2)	20 (41.7)	17 (29.8)	0.289
	Other oral hypoglycemic medications	15 (14.3)	9 (18.8)	6 (10.5)	0.358
**Successful short- and long-term weight reductions**					
8% weight loss after 6 months (“short-term respondents”)		34 (32.4)	21 (43.8)	13 (22.8)	**0.038**
5% weight loss after 3 years (“long-term respondents”)		60 (59.4)	26 (56.5)	34 (61.8)	0.737
**Obesity prevalence**					
BMI category at baseline	Over-weight	17 (16.2)	9 (18.8)	8 (14.0)	0.836
	Obesity I	48 (45.7)	21 (43.8)	27 (47.4)	
	Obesity II	40 (38.1)	18 (37.5)	22 (38.6)	
BMI category at 1 year	Over-weight	42 (40.0)	22 (45.8)	20 (35.1)	0.241
	Obesity I	42 (40.0)	19 (39.6)	23 (40.4)	
	Obesity II	20 (19.0)	6 (12.5)	14 (24.6)	
	Obesity III	1 (1)	1 (2.1)	0 (0.0)	
BMI category at 3 years	Normal weight	1 (1)	1 (2.2)	0 (0.0)	0.436
	Over-weight	38 (37.6)	20 (43.5)	18 (32.7)	
	Obesity I	37 (36.6)	15 (32.6)	22 (40.0)	
	Obesity II	25 (24.8)	10 (21.7)	15 (27.3)	

N, number; SD, standard deviation; Er-MedDiet, energy-restricted Mediterranean diet; BMI, body mass index. Bold values denote statistical significance at *P* < 0.05.

### Changes in body weight, cardiovascular risk profile, and glycemic metabolism

After 1 and 3 years of intervention, the prevalence of obesity decreased a 23.8 and 21.4%, respectively. Moreover, after 6 months of intervention, participants showed large significant improvements in cardiovascular and glycemic factors, and the observed changes in weight, BMI, waist and hip circumferences, HbA1c, insulin, HOMA-IR, LDL-c, total cholesterol, triglycerides and blood pressure were maintained after 3 years (Supplementary Figure 1).

**FIGURE 2:**
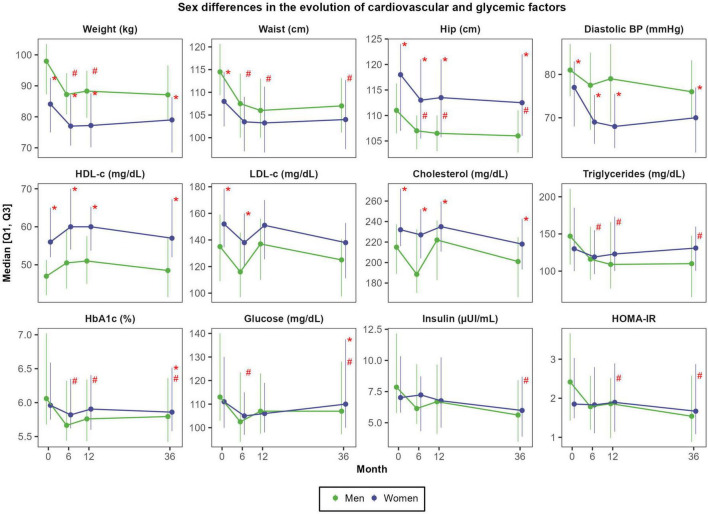
Sex differences in the evolution of cardiovascular and glycemic factors. *N* = 48 men and *N* = 57 women. Red asterisks (*) indicate significant sex differences at each time point (*P* < 0.05). Red hashes (#) indicate significant sex differences in the rate of change from baseline (*P* < 0.05). Dependent variables were normalized before estimating linear or linear mixed effects models (when deemed appropriated) using ordered quantile normalization (ORQ) transformation. Models were adjusted by sex, age, intervention group, and diagnostic of diabetes. Q1, first quartile; Q3, third quartile; BP, blood pressure; HbA1c, glycosylated hemoglobin; HDL-c, high-density lipoprotein cholesterol; HOMA-IR, Homeostasis Model Assessment of Insulin Resistance; LDL-c, low-density lipoprotein cholesterol.

Absolute weight reductions were more pronounced in men than in women: the average decrease after 3 years was −7.5 ± 6.5 kg in men (representing a reduction of 7.9 ± 6.0% of body weight) and −4.9 ± 5.1 kg in women (equivalent to a −6.0 ± 4.8% weight loss); *P* = 0.062 (Supplementary Table 1). After 3 years, men also experienced greater reductions than women in waist circumference (−7.7 ± 7.1 cm vs. −4.1 ± 5.4 cm; *P* = 0.008), glucose (−9.4 ± 20.9 mg/dL vs. 1.9 ± 23.8 mg/dL; *P* = 0.012), insulin (−3.4 ± 3.6 μUI/mL vs. −1.8 ± 3.5 μUI/mL; *P* = 0.029), HOMA-IR (−1.2 ± 1.4 vs. −0.4 ± 1.5 units; *P* = 0.008) and triglycerides (−46.1 ± 76.1 mg/dL vs. −14.5 ± 55.8 mg/dL; *P* = 0.005) (Figure 2).

### Changes in intervention adherence and food consumption

**FIGURE 3:**
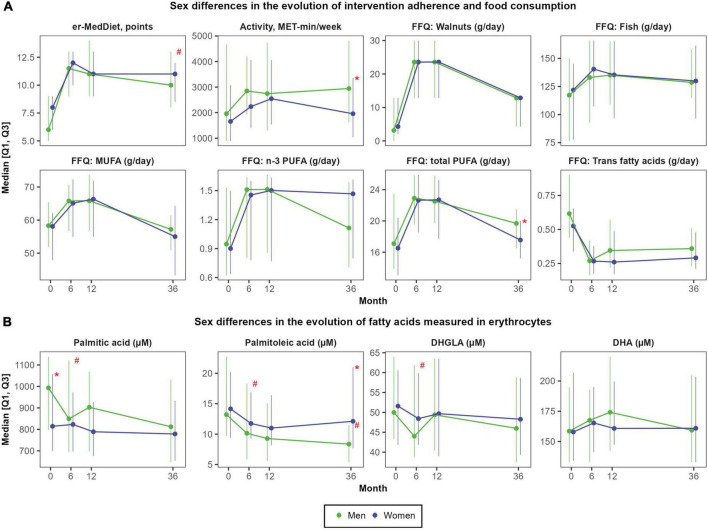
Sex differences in the evolution of self-reported food consumption and physical activity **(A)** and in the evolution of fatty acids measured in red blood cells **(B)**. *N* = 48 men and *N* = 57 women. Red asterisks (*) indicate significant sex differences at each time point (*P* < 0.05). Red hashes (#) indicate significant sex differences in the rate of change from baseline (*P* < 0.05). Dependent variables were normalized before estimating linear or linear mixed effects models (when deemed appropriated) using ordered quantile normalization (ORQ) transformation. Models were adjusted by sex, age, intervention group, and diagnostic of diabetes. Q1, first quartile; Q3, third quartile; DHA, docosahexaenoic acid; DHGLA, dihomo-gamma-linolenic acid; Er-MedDiet, energy-reduced Mediterranean diet; FFQ, food frequency questionnaire; MET, metabolic equivalent tasks; MUFA, monounsaturated fatty acids; PUFA, polyunsaturated fatty acids.

On the one hand, there were large improvements in MedDiet adherence after 6 months (+4.0 ± 3.1 points; Cohen’s d of 1.52; *P* < 0.001), which were sustained during the follow-up (average er-MedDiet score between 10 and 11 points) (Supplementary Figure 2). Accordingly, after 3 years, the proportion of participants with a high er-MedDiet adherence increased to 56.2 and 49.5%, respectively (data not shown). Men and women did not differ in MedDiet adherence during the intervention (Figure 3A), although men presented greater improvements in MedDiet adherence after 3 years (+3.9 ± 2.9 points vs. +2.5 ± 2.9 points; *P* = 0.017) due to their lower baseline adherence to this dietary pattern. On the other hand, changes in physical activity were more sustained in men than in women, since both sexes increased the levels of physical activity after 6 months and 1 year (mean change of 629.6 ± 2059.9 MET-min/week; *d* = 0.29; *P* < 0.001) but women recovered baseline levels after 3 years (Supplementary Table 2). Men also presented higher total energy intake than women at baseline (2510 ± 711 vs. 2169 ± 465 Kcal/day; *P* = 0.005) and during all the follow-up, although no sex differences were observed in total energy intake change over the 3 years of follow-up.

There were not sex differences in the consumption of high-fat foods. Nuts were the food group with the largest increase after 12 months, from 15.8 ± 15.6 g/day to 39.6 ± 21.7 g/day (*d* = 1.26; *P* < 0.001) (Supplementary Figure 2). The consumption of EVOO also increased after 1 year from 35.2 ± 18.9 g/day to 47.0 ± 10.8 g/day (*d* = 0.76, *P* < 0.001), but almost recovered baseline consumption after 3 years (*d* = 0.27, *P* = 0.045). The consumption of fatty fish also increased after 1 and 3 years, from 37.3 ± 24.1 g/day to 48.0 ± 23.0 g/day (*d* = 0.46, *P* < 0.001).

### Changes in fatty acids

In the overall population, mean changes in fatty acids concentrations were small and only significant for palmitoleic acid after 6 months (reduction of −4.7 ± 13.8 μM; *d* = −0.34; *P* < 0.001) and 3 years (reduction of −2.9 ± 15.5 μM; *d* = −0.30; *P* < 0.001), DHGLA after 6 months (reduction of −4.0 ± 19.4 μM; *d* = −0.20; *P* = 0.008), and the omega-3 fatty acids EPA (increase of +3.8 ± 15.0 μM after 1 year; *d* = 0.29; *P* = 0.006) and DHA (increase of +23.6 ± 101.9 μM after 1 year; *d* = 0.25, *P* = 0.026) (Supplementary Figure 3).

However, there were large sex differences in change in multiple fatty acids over time (Supplementary Table 3). After 6 months, the concentration of palmitic acid, palmitoleic acid, stearic acid, oleic acid, LA, ARA and DHGLA decreased in men but did not change in women (Figure 3B). After 3 years, concentrations of palmitoleic acid also decreased in men but did not change in women (−7.3 ± 17.2 μM vs. +0.6 ± 13.2 μM; *P* = 0.002). The evolution of fatty acids was not affected by the allocation of participants to the intervention group (er-MedDiet) or to the control group (traditional MedDiet) (Supplementary Figure 4).

### Changes in endocannabinoids and *N*-acylethanolamines

Peak changes in most eCBs and NAEs were observed after 6 months of intervention (Supplementary Table 4). 2-AG presented a small decrease at 6 months that remained stable over the 3 years of intervention (relative change of −10.6 ± 41.5%; *d* = −0.29; *P* = 0.004). Contrarily, the ratio OEA/PEA steadily increased during the intervention (relative change after 3 years of +4.5 ± 9.6%; *d* = 0.58; *P* < 0.001). In turn, NAEs concentrations (AEA, DEA, DGLEA, LEA, PEA, POEA, and SEA) slightly decreased after 6 months (relative change ranging −3.4% to −7.0%; *P* < 0.05) but recovered the baseline concentrations after 1 year and remained stable after 3 years, except for AEA which slightly increased at 3 years (+13.1 ± 38.9%; *d* = 0.25; *P* = 0.019). OEA remained rather stable until the third year, where it presented a moderate increase (+12.4 ± 30.7; *d* = 0.35; *P* = 0.002). An inverse U-shape curve was observed for OEA/AEA ratio, increasing from baseline to 6 months and 1 year (+7.0 ± 15.8%; *d* = 0.35; *P* < 0.001), and then decreasing at 3 years, recovering the baseline concentrations. Ultimately, the ratio DHEA/AEA slightly increased after 6 months (+6.5 ± 24.1%; *d* = 0.10; *P* = 0.054), whereas the ratio PEA/AEA remained stable after 6 months and 1 year but decreased after 3 years (−4.1 ± 17.9%; *d* = −0.37; *P* = 0.002).

**FIGURE 4:**
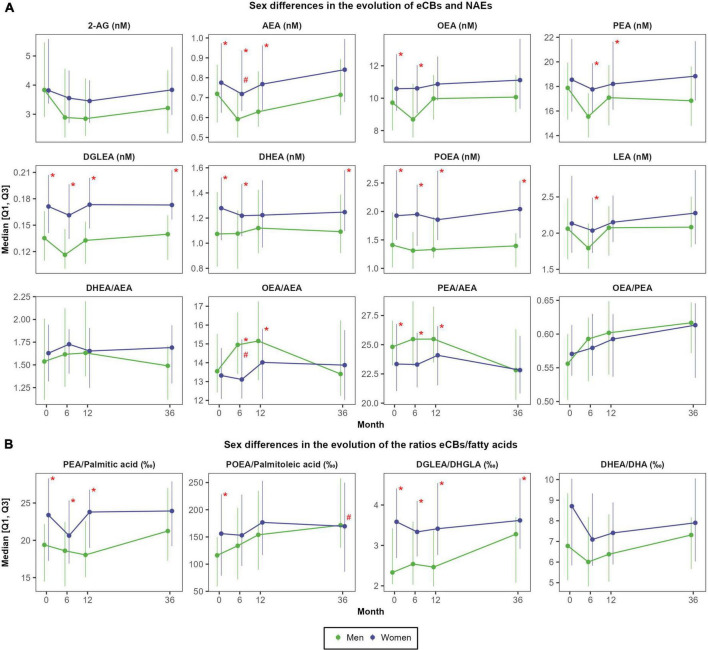
Sex differences in the evolution of eCBs concentrations **(A)** and in the evolution of the ratios between eCBs and their precursor fatty acids **(B)**. *N* = 48 men and *N* = 57 women. Red asterisks (*) indicate significant sex differences at each time point (*P* < 0.05). Red hashes (#) indicate significant sex differences in the rate of change from baseline (*P* < 0.05). Dependent variables were normalized before estimating linear or linear mixed effects models (when deemed appropriated) using ordered quantile normalization (ORQ) transformation. Models were adjusted by sex, age, intervention group, and diagnostic of diabetes. Q1, first quartile; Q3, third quartile; 2-AG, 2-arachidonoylglycerol; AEA, anandamide or *N-*arachidonoyl-ethanolamine; DHEA, *N-*docosahexaenoylethanolamine; DGLEA, *N-*dihomo-γ-linolenoyl ethanolamide; DHA, docosahexaenoic acid; DHGLA, dihomo-gamma-linolenic acid; eCBs, endocannabinoids; LEA, *N-*linoleoylethanolamine; NAEs, *N-*acylethanolamines; OEA, oleoylethanolamide; PEA, palmitoylethanolamide; POEA, *N-*palmitoleoylethanolamine.

As shown in Figure 4A, there were moderate-to-large sex differences in NAEs concentrations at each time point. Women presented higher concentrations of DGLEA (Cohen’s d effect size of sex differences ranging 0.77 to 1.05), POEA (*d* = 0.54 to 0.91), SEA (*d* = 0.59 to 0.97), DHEA (*d* = 0.47 to 0.60) and AEA (*d* = 0.31 to 0.54), whereas the ratios OEA/AEA and PEA/AEA were higher in men, particularly after 6 months (*d* = −0.48 and *d* = −0.54, respectively) (Supplementary Table 5). Moreover, the increase in OEA/AEA at 6 months was more pronounced in men than in women (mean change of +9.7 ± 14.2% vs. +2.5 ± 10.1%; *d* = −0.48; *P* < 0.001). Conversely, 2-AG concentrations and the ratio OEA/PEA did not differ between sexes.

Ultimately, participants allocated to the intervention group (energy-restricted MedDiet intervention) presented greater increases in the ratios OEA/AEA and OEA/PEA after 1 and 3 years than participants allocated to the active control group with a more traditional MedDiet intervention without energy restriction (Supplementary Figure 5). Specifically, the average relative change in OEA/AEA after 1 year was +11.2 ± 16.5% in the intervention group and +2.7 ± 13.9% in the control group (*d* = 0.61; *P* = 0.012); and after 3 years these values were +5.3 ± 16.5% vs. −0.8 ± 13.4% (*d* = 0.39; *P* = 0.047). In turn, the ratio OEA/PEA increased +6.4 ± 11.7% in the intervention group and +2.7 ± 13.9% in the control group (*d* = 0.42; *P* = 0.045) after 1 year, and these values after 3 years were +12.1 ± 14.4% for the intervention group and +4.3 ± 13.0% for the control group (*d* = 0.60; *P* = 0.003).

### Changes in the ratios between endocannabinoids or *N*-acylethanolamines and respective fatty acid precursors

The ratios eCBs/fatty acids followed a similar trajectory than the ones observed for eCBs alone except for the ratio POEA/palmitoleic acid (Figure 4B). Specifically, the ratio POEA/palmitoleic acid moderately increased after 1 year (mean change of +45.2 ± 120.9‰; *d* = 0.45; *P* < 0.001), and such changes were also sustained after 3 years (mean change of +51.4 ± 131.7‰; *d* = 0.50; *P* < 0.001) (Supplementary Figure 6). As observed for individual NAEs, women presented higher ratios AEA/ARA, DGLEA/DHGLA, PEA/palmitic acid, and SEA/stearic acid than men during all the follow-up (Supplementary Table 6). Intervention and control groups did not differ in the ratios eCBs/fatty acids (Supplementary Figure 7).

In men, a weak linear relationship between baseline fatty acids concentrations and their derived eCBs was only found for palmitoleic acid and POEA (*r* = 0.32; *P* = 0.026), palmitic acid and PEA (*r* = 0.36; *P* = 0.013), and between DHGLA and DGLEA (*r* = 0.36; *P* = 0.013). In addition, EPA but not DHA moderately correlated with DHEA (*r* = 0.48; *P* < 0.001). In women, plasma concentrations of eCBs did not relate with the concentrations of their precursor fatty acids in red blood cells, except for DHEA which correlated with both DHA (*r* = 0.34; *P* = 0.010) and EPA (*r* = 39; *P* = 0.002) (Supplementary Data 1). Finally, 6-months changes in eCBs and NAEs did not correlate with the respective changes in their precursor fatty acids, neither in men nor in women (Supplementary Figure 8).

### Baseline correlations between endocannabinoids, dietary and metabolic factors

**FIGURE 5:**
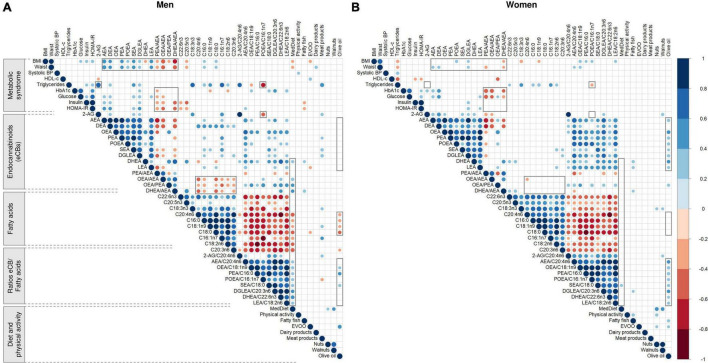
Representation of the Pearson’s r correlation matrix between baseline metabolic syndrome characteristics, diet, endocannabinoids and fatty acids in men **(A)** and women **(B)**. *N* = 48 men and *N* = 57 women. Blue and red cells indicate positive and negative correlations, respectively. Empty cells indicate non-significant correlations (*P* > 0.05). Areas in gray highlight some sex differences in the patterns of correlations. Values were normalized before estimating Pearson’s correlation, using ordered quantile normalization (ORQ) transformation. BMI, body mass index; BP, blood pressure; HbA1c, glycosylated hemoglobin; HDL-c, high-density lipoprotein cholesterol; HOMA-IR, Homeostasis Model Assessment of Insulin Resistance; LDL-c, low-density lipoprotein cholesterol; 2-AG, 2-arachidonoylglycerol; AEA, anandamide or *N-*arachidonoyl-ethanolamine; DHEA, *N-*docosahexaenoylethanolamine; DGLEA, *N-*dihomo-γ-linolenoyl ethanolamide; LEA, *N-*linoleoylethanolamine; OEA, oleoylethanolamide; PEA, palmitoylethanolamide; POEA, *N-*palmitoleoylethanolamine; C16:0, palmitic acid; C16:1n7, palmitoleic acid; C18:0, stearic acid; C18:1n9, oleic acid; C18:2n6, linoleic acid (LA); C18:3n3, alpha-linolenic acid (ALA); C20:3n6, dihomo-gamma-linolenic acid (DHGLA); C20:4n6, arachidonic acid (ARA); C20:5n3, eicosapentaenoic acid (EPA); C22:6n3, docosahexaenoic acid (DHA); Er-MedDiet, energy-reduced Mediterranean diet; EVOO, extra virgin olive oil.

In men, baseline adherence to the er-MedDiet positively correlated with DHEA (*r* = 0.35; *P* = 0.015) and the ratios DHEA/AEA (*r* = 0.41; *P* = 0.004), OEA/PEA (*r* = 0.41; *P* = 0.004) and OEA/AEA (*r* = 0.38; *P* = 0.008) (Figure 5A). In turn, the self-reported consumption of fatty fish positively correlated with DHEA (*r* = 0.29; *P* = 0.042) and especially the ratio DHEA/AEA (*r* = 0.35; *P* = 0.015) but did not significantly correlate with the blood concentrations of n3-PUFA although a trend was observed for EPA (*r* = 0.28; *P* = 0.057). On the other hand, BMI and waist circumference showed moderate negative correlations with the ratios of eCBs, especially DHEA/AEA (*r* = −0.63 and −0.56; *P* < 0.001) and OEA/AEA (*r* = −0.58 and *r* = −0.52; *P* < 0.001). Moreover, both anthropometric measures positively correlated with AEA (*r* = 0.53; *P* < 0.001) and other NAEs (except DHEA) and with saturated fatty acids, and they negatively correlated with EPA (*r* = −0.32 and *r* = −0.34; *P* < 0.05). Fasting plasma glucose, insulin, and particularly HOMA-IR were positively correlated with AEA, DEA, SEA and DGLEA (*r* ranging 0.30 to 0.40; *P* < 0.05) and negatively correlated with the ratios of eCBs, especially OEA/AEA and DHE/AEA (*r* = −0.42; *P* = 0.003). Moreover, insulin and HOMA-IR negatively correlated with the fish-derived omega-3 fatty acids EPA (*r* = −0.37; *P* = 0.010) and DHA (*r* = −0.33; *P* = 0.022). On the other hand, triglycerides strongly correlated with 2-AG (*r* = 0.75; *P* < 0.001) and the ratios 2-AG/ARA (*r* = 0.62; *P* < 0.001) and POEA/palmitoleic acid (*r* = −0.65; *P* < 0.001). Triglycerides also moderately correlated with DEA, SEA and DGLEA, palmitoleic acid, ALA, palmitic acid, LA and the ratio OEA/oleic acid.

In women, most of these linear relationships were not observed, including those between MedDiet adherence and eCBs, as well as between triglycerides and 2-AG (Figure 5B). Women also showed no consistent correlations between fatty acids and the ratios OEA/AEA, OEA/PEA and DHEA/AEA, or between NAEs and anthropometric factors. Moreover, olive oil consumption positively correlated with NAEs, particularly OEA (*r* = 0.46; *P* < 0.001) and DHEA (*r* = 0.43; *P* < 0.001). Finally, the ratios PEA/AEA, OEA/AEA and DHEA/AEA negatively correlated with the glycemic profile, including both HbA1c and glucose but excluding insulin and HOMA-IR. See values in Supplementary Data 1.

### Association between endocannabinoids and *N*-acylethanolamines, weight loss, and insulin resistance

We ultimately analyzed the association between the relative change in eCBs and the probability of responding to the intervention in terms of achieving successful weight reductions at the short-term (i.e., 8% body weight reductions after 6 months) and the long-term (i.e., 5% body weight reductions after 3 years).

Successful short-term weight reductions of at least 8% were achieved by the 43.8% of men and the 22.8% of women (*P* = 0.038). Men and women who achieved this weight reduction goal after 6 months were categorized as “short-term respondents” (Supplementary Table 7). Respondents and non-respondents significantly differed in MedDiet adherence after 6 months but did not differ in physical activity levels or in total energy intake. In men, respondents and non-respondents significantly differed in the rate of change in OEA/PEA, which increased in respondents but did not change in non-respondents (+11.2 ± 9.3% vs. +2.7 ± 10.2%; *P* = 0.002). Men respondents also presented greater increases in OEA/AEA (+13.7 ± 14.9% vs. −6.9 ± 13.2%; *P* = 0.077) and greater reductions in 2-AG (−23.0 ± 28.9% vs. −6.0 ± 45.9%; *P* = 0.093). In turn, women respondents presented lower AEA concentrations after 6 months than women non-respondents (0.64 ± 0.15 nM vs. 0.83 ± 0.23 nM; *P* = 0.003) and experienced greater reductions in this eCB (−11.8 ± 15.3% vs. 2.6 ± 25.4%; *P* = 0.055). The same pattern of differences between women respondents and non-respondents were observed for all NAEs, as well as for the ratio OEA/AEA.

**FIGURE 6:**
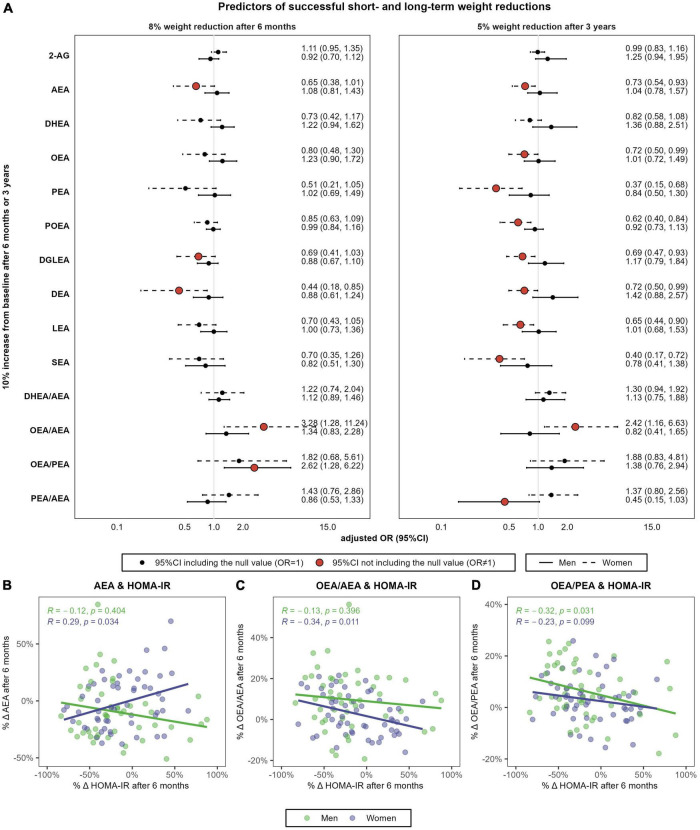
Sex-specific association between relative changes in eCBs, NAEs and their ratios, and the achievement of 8% weight reductions after 6 months or 5% weight reductions after 3 years **(A)** and correlation between 6-months percentual changes in HOMA-IR and the respective change in AEA **(B)**, OEA/AEA **(C)** and OEA/PEA **(D)**. *N* = 48 men and *N* = 57 women. **(A)** Shows multivariable-adjusted odds ratio (OR) and 95% confidence intervals (95%CI). Logistic regression models were stratified by sex and adjusted by baseline body weight, intervention group, age, and diagnostic of diabetes. **(C,D)** Shows the Spearman’s rank correlations coefficient and *P*-values. HOMA-IR, Homeostasis Model Assessment of Insulin Resistance; 2-AG, 2-arachidonoylglycerol; AEA, anandamide or *N-*arachidonoyl-ethanolamine; DHEA, *N-*docosahexaenoylethanolamine; DGLEA, *N-*dihomo-γ-linolenoyl ethanolamide; LEA, *N-*linoleoylethanolamine; OEA, oleoylethanolamide; PEA, palmitoylethanolamide; POEA, *N-*palmitoleoylethanolamine.

After 6 months, a 10% increase in AEA was associated with 35% decreased odds of 8% weight loss in women (OR = 0.65, 95%CI 0.38, 1.01) (Figure 6A). Similarly, increases in DEA and DGLEA after 6 months were also associated with decreased odds of 8% weight reductions in women (for DEA: OR = 0.44, 95%CI 0.18, 0.85; for DGLEA: OR = 0.69, 95%CI 0.41, 1.03), and similar trends were observed for DHEA, PEA, POEA, LEA, and SEA. 6-months changes in AEA positively correlated with changes in HOMA-IR in women (*r* = 0.29, *P* = 0.034) (Figure 6B). In addition, a 10% increase in the ratio OEA/AEA was associated with threefold increased odds of achieving this weight reduction goal in women (OR = 3.28, 95%CI 1.28, 11.24), and such OEA/AEA changes were negatively correlated with changes in insulin resistance (*r* = −0.34, *p* = 0.011) (Figure 6C). In men, an increase in the ratio OEA/PEA was associated with greater odds of responding to the intervention after 6 months (OR = 2.62, 95%CI 1.28, 6.22), and it negatively correlated with changes in insulin resistance (*r* = −0.32, *P* = 0.031) (Figure 6D).

Successful long-term weight reductions of at least 5% of body weight after 3 years were achieved by the 56.6% of men and the 61.8% of women (*P* = 0.737) (Supplementary Table 8). Men categorized as long-term respondents only differed than non-respondents in MedDiet change after 3 years (+5.0 ± 2.7 vs. +1.9 ± 2.1 points; *P* = 0.002), in baseline 2-AG concentrations (4.01 ± 3.34 nM in respondents vs. 6.44 ± 5.15 nM in non-respondents; *P* = 0.002) and in baseline DHEA/AEA ratio (1.56 ± 0.61 in respondents vs. 1.71 ± 0.63 in non-respondents; *P* = 0.026). Similarly, women respondents, compared to women non-respondents, experienced greater increases in MedDiet adherence (+3.1 ± 2.8 vs. 1.4 ± 2.9 points; *P* = 0.022), as well as in OEA/AEA (+5.2 ± 16.5% vs. −4.6 ± 12.2%; *P* = 0.024) and OEA/PEA (+9.5 ± 12.4% vs. +1.0 ± 7.7%; *P* = 0.007).

After 3 years, changes in all NAEs except DHEA were significantly and negatively associated with 5% weight reductions in women (Figure 6A), including AEA (OR = 0.73), OEA (OR = 0.72), PEA (OR = 0.37), POEA (OR = 0.62), DGLEA (OR = 0.69), DEA (OR = 0.72), LEA (OR = 0.65) and SEA (OR = 0.40). In women, increases in the ratio OEA/AEA were also associated with the achievement of successful long-term weight reductions (OR = 2.42, 95%CI 1.16, 6.63). Finally, changes in the ratio PEA/AEA were negatively associated with 5% weight reductions after 3 years in men (OR = 0.45, 95%CI 0.15, 1.03). However, changes in AEA and NAEs ratios after 3 years did not correlate with changes in HOMA-IR, neither in men nor in women (results not shown).

The studied relations were linear for all eCBs and NAEs at all time points, as the inclusion of splines did not improve model fit (Supplementary Table 9).

## Discussion

### Main findings

In this study we were interested in analyzing sex differences in the interplay between eCBs, NAEs, insulin resistance and weight loss, during 3 years of lifestyle intervention with MedDiet in a population of older adults with overweight or obesity and metabolic syndrome. We observed marked sex differences in AEA and NAEs concentrations, which were all higher in women, and in the efficacy the lifestyle intervention, which produced greater glycemic and cardiovascular benefits in men. In both sexes, we also observed a persistent decrease in 2-AG and an increase in the ratio OEA/PEA during the 3 years of follow-up. In addition, reductions in AEA and NAEs were detected after 6 months. Changes in eCBs and NAEs were not influenced by changes in their precursor fatty acids. Finally, changes in AEA and the ratio OEA/AEA were associated with the achievement of 8% weight reductions after 6 months in women and correlated with changes in insulin resistance. In men increases the ratios OEA/PEA and PEA/AEA appeared to be associated with clinically meaningful weight reductions at the short- and long-term, respectively.

### Sex differences in the effect of the Mediterranean diet intervention on cardiometabolic health

Mediterranean diet adherence increased during the first 6 months of intervention and remained high after 1 and 3 years. Participants also increased their levels of physical activity after 1 year. Moreover, they reported a lower intake of saturated fatty acids and trans fatty acids, and a higher consumption of fatty fish and nuts. They also increased the consumption of olive oil and total EVOO (crude and for cooking) even though baseline consumption (about 45 g/day of total olive oil and 35 g/day of EVOO) was above the recommended minimum (at least 20 g/day of olive oil, mainly virgin) (58). Moreover, they experienced average weight reductions of 7% of body weight after 6 months that were sustained over the 3 years of intervention. Participants also showed sustained improvements in the glycemic profile, including HbA1c, glucose, insulin and HOMA-IR, as well as, in metabolic syndrome characteristics, including waist circumference, triglycerides, blood pressure and HDL-c.

The observed cardiometabolic benefits were greater in men than in women despite no sex differences in MedDiet adherence or food consumption. These results were independent of baseline characteristics (e.g., greater body weight of men). Our results add to existing evidence about the sex-specific effects of a lifestyle intervention on weight loss, cardiometabolic health and insulin resistance (9, 59–63).

In older adults, alterations in glucose homeostasis have been shown to determine the dietary weight loss success (64, 65) and could partly explain the observed sex-specific response to a MedDiet intervention. Specifically in women, the drop in sex hormones after menopause induces a redistribution of adipose tissue from the lower part of the body to the central part, which results in hypertrophy of the intra-abdominal adipose tissue and induces a pro-inflammatory response (9). These changes are accompanied by a reduction in the fat oxidative capacity of skeletal muscle and liver, leading to excessive lipid accumulation in these organs and impairing their insulin sensitivity (9). To act as a satiety signal, glucose needs to be taken up by cells in the liver, muscle, adipose tissue and brain (65). Therefore, the poorer glucose metabolism of our population of post-menopause women aged 60–75 years, compared to men of the same age group, could induce a weaker satiating effect of food or increased resistance to weight loss, leading to minor cardiometabolic benefits after 3 years of MedDiet intervention.

### Sex differences in circulating endocannabinoids and *N*-acylethanolamines

We performed a comprehensive descriptive analysis by sex of the evolution of fatty acids and eCBs, and we examined for the first time the ratio DHEA/AEA and the ratios between eCBs and their precursor fatty acids. Contrary to previous studies in younger individuals (28), men and women did not differ in 2-AG concentrations, although globally were higher than in the normo-weight population (66). Menopause has been identified as one of the factors that affects circulating 2-AG, as their concentrations in lean and overweight menopausal women do not differ than those observed in obese menopausal women (26). This may explain why we have not found sex differences in 2-AG despite men presenting higher body weight, visceral fat or diastolic blood pressure than women.

In men we observed that 2-AG concentrations strongly correlated with triglycerides in agreement with a previous study (67). We did not find this relationship in women, as it has also been reported (28). The biological mechanism could be related to the modulation of protein Apolipoprotein E (ApoE), which is involved in the transport of lipids, though sex-differences have not investigated (68). However, sex differences in the effect of the *APOE* genotype have already been reported in the context of neurodegenerative diseases (69), along with sex differences in hepatic fat oxidation or *de novo* lipogenesis (9). Thus, the interplay between sex, *APOE* genotype, triglycerides and 2-AG in the context of menopause should be further explored in future studies.

On the other hand, AEA and derived NAEs were more elevated in women compared to men, although globally were equivalent to the ones already reported in post-menopausal women and obese men (23). In turn, men displayed higher ratios DHEA/AEA, OEA/AEA, and PEA/AEA. Sex differences in AEA have been also observed in previous studies (24), though no sex differences in NAEs were detected in younger cohorts (aged 30–50 years) (28, 33). In both sexes, we also observed that the ratios PEA/AEA, OEA/AEA, and DHEA/AEA were strong indicators of a healthier glycemic status as they negatively correlated with insulin and HOMA-IR in men, and with glucose and HbA1c in women. In turn, AEA positively correlated with these glycemic parameters in both sexes. Therefore, insulin resistance characteristic of the menopause state could explain the elevated NAEs concentrations in our population of women (23).

### Sex differences in the Mediterranean diet modulation of endocannabinoids, *N*-acylethanolamines, and fatty acids

The MedDiet intervention resulted in small changes in eCBs and NAEs, as reported in a shorter nutritional intervention study of 2 months (33). However, the longer intervention period of the present report has allowed to monitor the sustainability of changes over time. In this context, short-term reductions in 2-AG or AEA and derived NAEs were observed after 6 months when most metabolic changes occur and probably earlier considering the previous report of a shorter MedDiet intervention (33). Concentrations of 2-AG were steadily lower during the intervention period, while AEA and NAEs tended to recover baseline concentrations after 1 year and AEA even increased after 3 years. The sustained decrease in 2-AG after a MedDiet intervention could be associated with a reduction in visceral fat and triacylglycerol levels (70). Accordingly, 2-AG trajectory paralleled the trajectories of triglycerides, BMI, waist and hip circumferences, blood pressure and fasting plasma glucose. In turn, the evolution of NAEs reflected that of LDL-c and total cholesterol.

Throughout the study, we also observed that women were more resistant to changes in circulating NAEs than men. The underlying mechanism explaining such sex differences in NAEs concentrations and modulation after a MedDiet intervention could be linked to menopause-related impairments in glucose homeostasis (23), given that 6-months changes in AEA, OEA/AEA and OEA/PEA coupled with changes in insulin resistance.

Altogether, changes in eCBs seem to operate independently of fatty acid concentrations, probably due to the multiple pathways and intermediary compounds involved in the synthesis of eCBs and NAEs. For instance, ARA from phospholipids is mainly converted to AEA by a pathway that involves the biosynthesis of N-arachidonoyl phosphatidylethanolamine (NAPE) and its conversion to AEA by the enzyme N-acylphosphatidylethanolamine-hydrolyzing phospholipase D (NAPE-PLD) (71, 72). NAPE generation could be the initial rate-limiting step in the food-induced production of NAEs (71).

We also observed that the average trajectory of POEA/palmitoleic acid differed from the one observed for POEA alone, though the evolution of the remaining ratios eCBs/fatty acids (e.g., 2-AG/ARA, DHEA/DHA, OEA/oleic acid, LEA/linoleic acid) resemble that of individual eCBs. These results suggest a constant or increased POEA synthesis despite a reduction of palmitoleic acid concentrations during the MedDiet intervention. In animal models, POEA has been attributed with beneficial effects on body weight, liver functioning, inflammation status and dyslipidemia (73). These effects could explain the enhanced production of this eCB-like compound regardless of reductions in its precursor fatty acid during the intervention.

Unlike what was observed for eCBs, we did not find sex-differences in baseline concentrations of fatty acids. These results contrast with findings from other studies in individuals >60 years showing a higher proportion (reported as a percentage of total) of n6-PUFA and palmitoleic acid in women compared to men (74). The method used to express the levels of circulating fatty acids (i.e., as concentrations vs. a percentage of total) could affect the identification of sex-differences and the relationship between fatty acids and diet or metabolic factors (75).

To our knowledge, this is the first study in examining sex-differences in fatty acids concentrations during 3 years of MedDiet intervention. We observed that the magnitude of changes in fatty acids was generally small, which is in line with previous short-term studies with MedDiet (35, 76, 77). Moreover, changes in fatty acids presented a high variability across subjects and particularly across sexes. As observed for eCBs, women were more resistant to changes in fatty acid concentrations than men. Palmitoleic acid is the fatty acid that was mostly modified during the intervention. Specifically, it decreased in men but remained stable in women. Its plasma and tissue concentrations are produced by *de novo* lipogenesis and are regulated by several hormones including insulin (78). Sex-specific alterations in insulin-resistance could partly explain the large differences at 6-months and 3-years in palmitoleic acid change between men and women (79, 80). Accordingly, elevated plasma concentrations of palmitoleic acid are usually associated with higher fasting glucose levels (81), although this was not replicated in our study. On the other hand, omega-3 and omega-6 fatty acids were also modulated after 1 year of intervention, particularly DHA and EPA that increased and DHGLA that decreased.

### Association between endocannabinoids, *N*-acylethanolamines ratios, weight loss, and insulin resistance

In the present study, greater adherence to the MedDiet during the intervention was the primary lifestyle factor that entailed successful weight reductions at the short- and long-term. In fact, physical activity levels did not differ between those who achieved 8% weight reductions after 6 months or 5% weight reductions after 3 years. This may be due to the greater intensity of the dietary component (individual counseling visits and free provision of EVOO and mixed nuts) compared to the physical activity component (based on recommendations delivered by dietitians) of the Predimed-Plus study (39). Therefore, it is more likely that changes in eCBs and NAEs during the Predimed-Plus lifestyle intervention were due to the cardiometabolic effects of the MedDiet component than to those of the physical activity component, although some studies have already reported the modulation of eCBs by physical activity (mainly after acute exercise) (82).

We show that an increase in peripheral NAEs concentrations, including AEA, is a negative predictor of the short- and long-term response to the MedDiet intervention in women irrespective of the baseline body weight. This was not found in men, probably because after 6 months both respondents (succeeding in the 8% weight loss goal) and non-respondents (not achieving this goal) decreased their AEA, whereas in women AEA decreased in respondents and did not change in non-respondents. In women, change in the ratio OEA/AEA was also identified as a significant predictor of weight loss success after 6 months and 3 years. In men, the relative abundance of PEA seems to influence the short- and long-term dietary weight loss success, as an increase in OEA/PEA at 6 months and a decrease in PEA/AEA after 3 years were significantly associated with the achievement of 8 and 5% weight reductions, respectively. Finally, we also show that changes in those eCBs (i.e., AEA) and NAEs ratios (i.e., OEA/PEA and OEA/AEA) that were associated with short-term weight reductions, also correlated with changes in insulin resistance.

The mechanism underlying the observed sex-specific associations between NAEs and weight loss could be related to the interplay between eCBs or NAEs, insulin resistance and gut intestinal barrier integrity (33). Accordingly, plasma concentrations of eCBs and NAEs have been related with the gut barrier integrity (83), and insulin signaling has been identified as an essential gatekeeper of intestinal barrier integrity (84). Therefore, those women with lower insulin resistance could have a healthier intestinal barrier and a better NAEs profile, and could be more susceptible to dietary-induced weight loss changes. However, this potential mechanism should be investigated in future studies.

Most NAEs modify or enhance the actions of eCBs, which is called “entourage effect” (85). Moreover, they display biological activities related to their interactions with other receptors (21). It is particular the role of OEA that acts on the gut-brain axis as a fat-derived signal that induces satiety *via* the vagus nerve and satiety-controlling centers in the brain stem by binding to PPARα receptors in enterocytes (71). PEA is also an agonist of the PPARα receptor, and in a recent study in animal models has been shown to restore white adipose tissue homeostasis (86). Both OEA and PEA are associated with a reduction in adipose tissue inflammation after gastric bypass in morbid obese patients (87). The alteration of some NAEs ratios after the intervention could be related to reduced inflammation (33) but this should be evaluated in future studies. The enzymes NAPE-PLD and FAAH are responsible for the synthesis and hydrolysis of NAEs and have shown to influence their circulating levels (88). Remarkably, genetic polymorphisms of FAAH have shown to modulate plasma concentrations of OEA and DHEA (89). However, the underlying mechanism behind changes in the relative abundance of NAEs in relation to weight loss, insulin resistance and inflammation should be explored in future studies, which may also investigate the consistency and origin of the observed sex-differences.

### Strengths and limitations

Strengths of this study include its longitudinal design with 3 years of follow-up, including 4 repeated measures per subject, as well as the wide range of eCBs, NAEs and fatty acids that were objectively measured in human samples, which provide detailed evidence about the long-term modulation of these compounds by the MedDiet-based lifestyle intervention. Moreover, all the analyses were stratified by sex, which has been identified as a major determinant for weight loss and eCB system modulation (23). Sex differences in cardiometabolic disease risk have long been reported in epidemiological and clinical contexts (90, 91), in addition to sex-specific differences in the response to interventions (92, 93), which supports the importance of following a sex-balanced research strategy (94). Regarding the analysis of eCBs, a strict harmonized sample collection with the lipase inhibitor orlistat and a laborious processing protocol was used in order to avoid artifactual differences between samples due to the natural presence of enzymatic activity in plasma (53). Additionally, there were no drop-outs during the 3 years of intervention (<5% of missing rates in body weight in the 3rd-year measure), so missing in all the studied factors was assumed to be completely at random (MCAR) and may not bias the obtained results. However, some limitations must be mentioned. The sample size evaluated is relatively small and results should be replicated in larger cohorts of individuals. Moreover, the studied population was restricted to older adults with metabolic syndrome and overweight or obesity. In addition, we used an active comparator as a control group (a healthy, traditional MedDiet), while the intensive intervention consisted of real-life changes in diet and physical activity. Therefore, the generalizability of our findings to younger individuals with and without metabolic syndrome or obesity, and with other dietary patterns and lifestyle interventions, remains to be studied.

## Conclusion

Altogether, our findings support sex as an important determinant of the plasma concentrations of eCBs and of the efficacy of a MedDiet intervention in terms of obesity-related metabolic factors. We also show the impact of changes in eCB balance on the regulation of body weight loss, and we suggest the metabolic connection of eCBs and their NAEs related eCBs with the glucose homeostasis. Overall, eCBs and NAEs might respond to short-term changes in energy metabolism, though more research is needed to understand the impact of long-term changes in these lipid mediators. Results from this study would contribute to a better understanding of the biological mechanisms that control appetite and energy utilization, and offer opportunities for improving lifestyle-based preventive strategies in the context of personalized nutrition.

## Data availability statement

The datasets presented in this article are not readily available because there are restrictions on the availability of data for the PREDIMED-Plus trial, due to the signed consent agreements around data sharing. Requestors wishing to access the PREDIMED-Plus dataset generated and/or analyzed during the current study can make a request to the PREDIMED-Plus trial Steering Committee chair. Requests to access the datasets should be directed to JS-S, jordi.salas@urv.cat.

## Ethics statement

The studies involving human participants were reviewed and approved by the Parc de Salut Mar Clinical Research Ethics Committee CEIm-PSMAR. The patients/participants provided their written informed consent to participate in this study.

## Author contributions

RT and MF designed the study and obtained funding for the study. AP and AB performed the ECBs measurements. AS-V and IL performed the fatty acids measurements. DM performed the biochemical analysis. NS-D performed the statistical analyses. NS-D, AP, and RT wrote the manuscript. JS-S, MM-G, DC, FF-A, BF, OC, AP, AB, AS-V, IL, DM, and MF contributed to a critical revision of the manuscript for key intellectual content. All authors have read and approved the final manuscript.

## Abbreviations

2-AG: 2-arachidonoylglycerol
AEA: anandamide or *N*-arachidonoyl-ethanolamine
ALA: alpha-linolenic acid
ARA: arachidonic acid
BMI: body mass index
BP: blood pressure
DGLEA: *N*-dihomo–linolenoyl ethanolamide
DHA: docosahexaenoic acid
DHEA: *N*-docosahexaenoylethanolamine
DHGLA: dihomo-gamma-linolenic acid
eCBs: endocannabinoids
EPA: eicosapentaenoic acid
Er-MedDiet: energy-reduced Mediterranean diet
EVOO: extra virgin olive oil
HbA1c: glycosylated hemoglobin
HDL-c: high-density lipoprotein cholesterol
HOMA-IR: homeostasis model assessment of insulin resistance
LA: linoleic acid
LDL-c: low-density lipoprotein cholesterol
LEA: *N-*linoleoylethanolamine
MedDiet: Mediterranean diet
NAEs: *N*-acylethanolamines
OEA: oleoylethanolamide
PEA: palmitoylethanolamide
POEA: *N*-palmitoleoylethanolamine.
