# Supplementary material for: Sex differences in endocannabinoids during 3 years of Mediterranean diet intervention: Association with insulin resistance and weight loss in a population with metabolic syndrome
Source: Front Nutr. 2022 Dec 1;9:1076677. doi: 10.3389/fnut.2022.1076677 (PMC9754217; doi:10.3389/fnut.2022.1076677)
Supplement: Supplementary file 1 [file Data_Sheet_1.PDF]

Supplementary Material

Index

Supplementary Figures 2

Supplementary Figure 1. Evolution of cardiovascular and glycemic factors over time in the overall population [N=105] 2

Supplementary Figure 2. Evolution of intervention adherence and food consumption in the overall population [N=105]. 3

Supplementary Figure 3. Evolution of fatty acids over time in the overall population [N=105]. 4

Supplementary Figure 4. Evolution of fatty acids over time by intervention group [N=52 IG and N=53 CG]. 5

Supplementary Figure 5. Evolution of endocannabinoids and NAEs by intervention group [N=52 IG and N=53 CG]. 6

Supplementary Figure 6. Evolution of the ratios eCB/fatty acids over time in the overall population [N=105]. 7

Supplementary Figure 7. Evolution of the ratios eCB/fatty acids over time by intervention group [N=52 IG and N=53 CG]. 8

Supplementary Figure 8. Scatter plots showing the correlations between change in eCBs or NAEs after 6 months and the respective change in their precursor fatty acids in men and women. 9

Supplementary Tables 10

Supplementary Table 1. Change in anthropometric, cardiovascular and glycemic factors from baseline to 6 months, 1 year and 3 years by sex 10

Supplementary Table 2. Change in intervention adherence and food consumption from baseline to 6 months, 1 year and 3 years by sex 12

Supplementary Table 3. Change in fatty acids from baseline to 6 months, 1 year and 3 years by sex 14

Supplementary Table 4. Change in eCBs concentrations from baseline to 6 months, 1 year and 3 years in the overall population 16

Supplementary Table 5. Description of changes in endocannabinoids concentrations from baseline to 6 months, 1 year and 3 years stratified by sex 18

Supplementary Table 6. Change in the ratios eCB/fatty acids from baseline to 6 months, 1 year and 3 years by sex 19

Supplementary Table 7. Description of 6-months changes in endocannabinoids and intervention factors according to the achievement of 8% weight loss after 6 months (respondents/non-respondents) 20

Supplementary Table 8. Description of 3-year changes in endocannabinoids and intervention factors according to the achievement of 5% weight loss after 3 years (respondents/non-respondents) 22

Supplementary Table 9. ANOVA table of Generalized Additive Model (GAM) results for the association between relative changes in eCBs, NAEs and their ratios, and the achievement of successful weight reductions, stratified by sex 24

Supplementary Figures

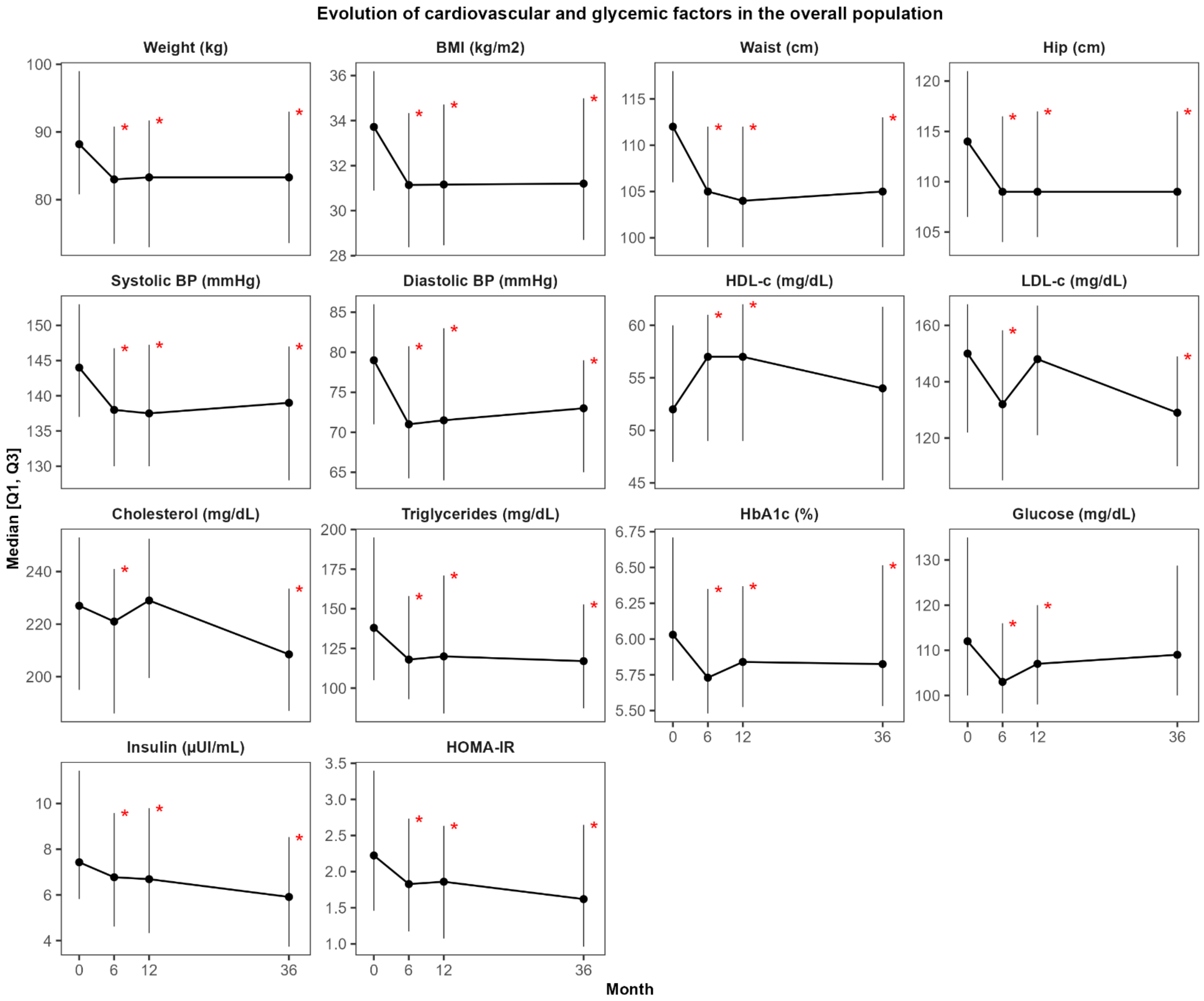

Supplementary Figure 1. Evolution of cardiovascular and glycemic factors over time in the overall population [N=105]

*Indicate significant changes from baseline (P≤0.05). Dependent variables were normalized before estimating linear or linear mixed effects models (when deemed appropriated) using ordered quantile normalization (ORQ) transformation. Models were adjusted by sex, age, intervention group and diagnostic of diabetes. Q1= first quartile. Q3= third quartile. BMI= body mass index. BP= blood pressure. HbA1c= glycosylated hemoglobin. HDL-c= high-density lipoprotein cholesterol. HOMA-IR= Homeostasis Model Assessment of Insulin Resistance. LDL-c= low-density lipoprotein cholesterol. N=number.

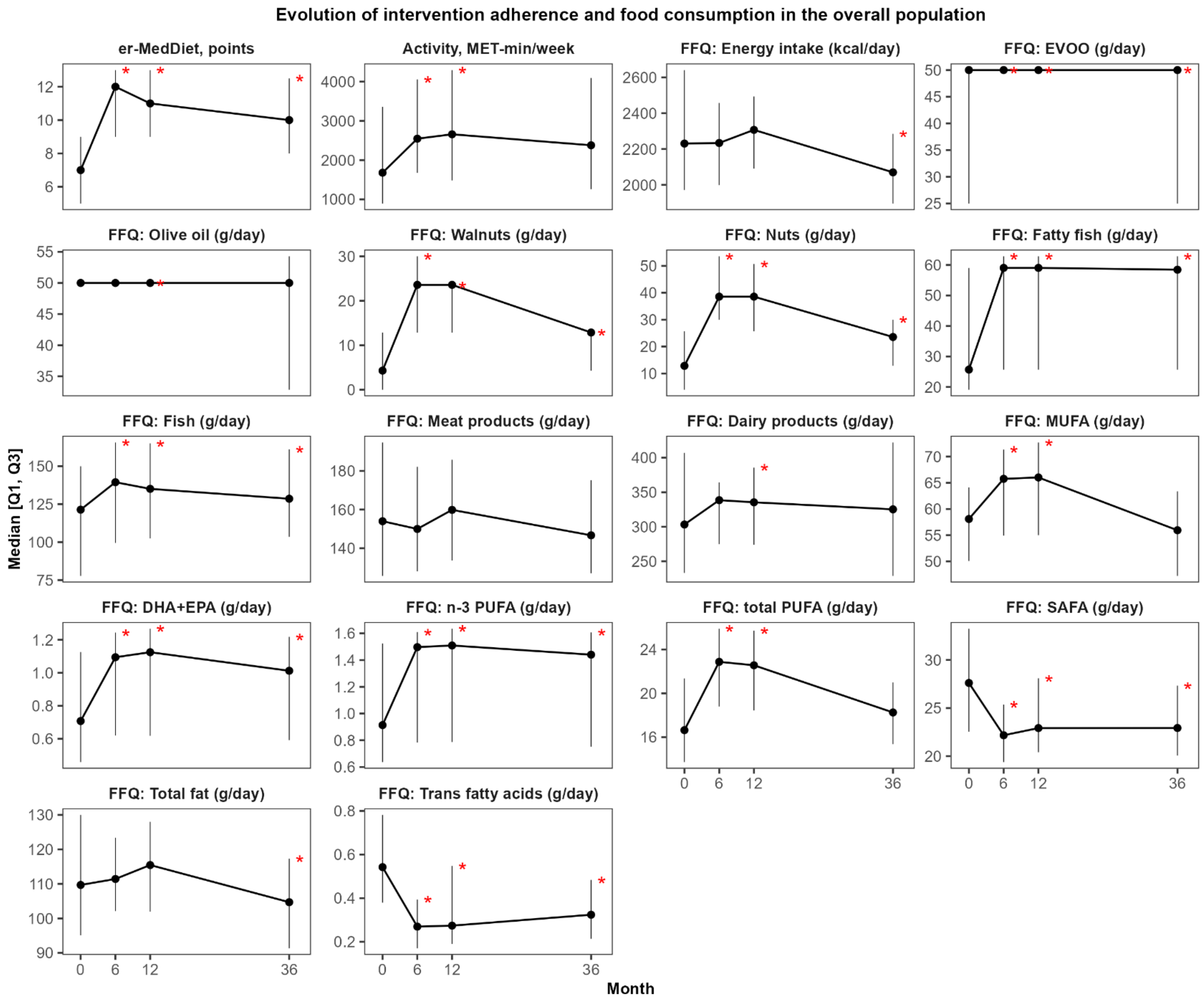

Supplementary Figure 2. Evolution of intervention adherence and food consumption in the overall population [N=105].

*Indicate significant changes from baseline (P≤0.05). Dependent variables were normalized before estimating linear or linear mixed effects models (when deemed appropriated) using ordered quantile normalization (ORQ) transformation. Models were adjusted by sex, age, intervention group and diagnostic of diabetes. Q1= first quartile. Q3= third quartile. Er-MedDiet= energy-reduced Mediterranean diet. EVOO= extra virgin olive oil. DHA= docosahexaenoic acid. EPA= eicosapentaenoic acid. FFQ= food frequency questionnaire. MET= metabolic equivalent tasks. MUFA= monounsaturated fatty acids. n3-PUFA= omega-3 polyunsaturated fatty acids. SAFA= saturated fatty acids.

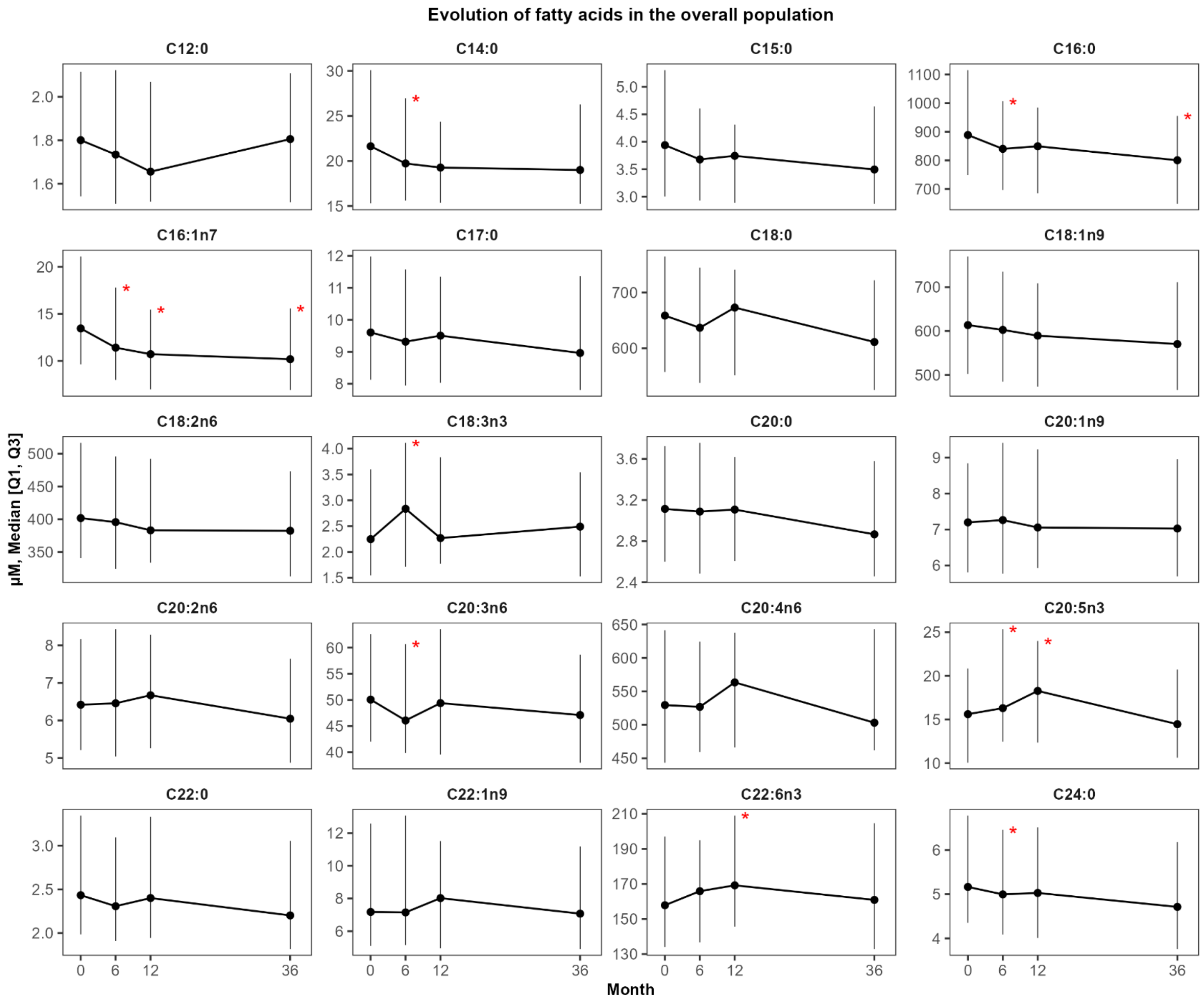

Supplementary Figure 3. Evolution of fatty acids over time in the overall population [N=105].

*Indicate significant changes from baseline (P≤0.05). Dependent variables were normalized before estimating linear or linear mixed effects models (when deemed appropriated) using ordered quantile normalization (ORQ) transformation. Models were adjusted by sex, age, intervention group and diagnostic of diabetes. Q1= first quartile. Q3= third quartile. List of common names for each fatty acid: C12:0= lauric acid. C14:0= myristic acid. C15:0= pentadecylic acid. C16:0= palmitic acid. C16:1n7= palmitoleic acid. C17:0= margaric acid. C18:0= stearic acid. C18:1n9= oleic acid. C18:2n6= linoleic acid (LA). C18:3n3= alpha-linolenic acid (ALA), C20:0= arachidic acid. C20:1n9= *cis*-11-eicosenoic acid. C20:2n6= all *cis*-11,14-eicosadienoic acid. C20:3n6= dihomo-gamma-linolenic acid (DHGLA). C20:4n6= arachidonic acid (ARA). C20:5n3= eicosapentaenoic acid (EPA). C22:0= behenic acid. C22:1n9= erucic acid. C22:6n3= docosahexaenoic acid (DHA). C24:0= lignoceric acid.

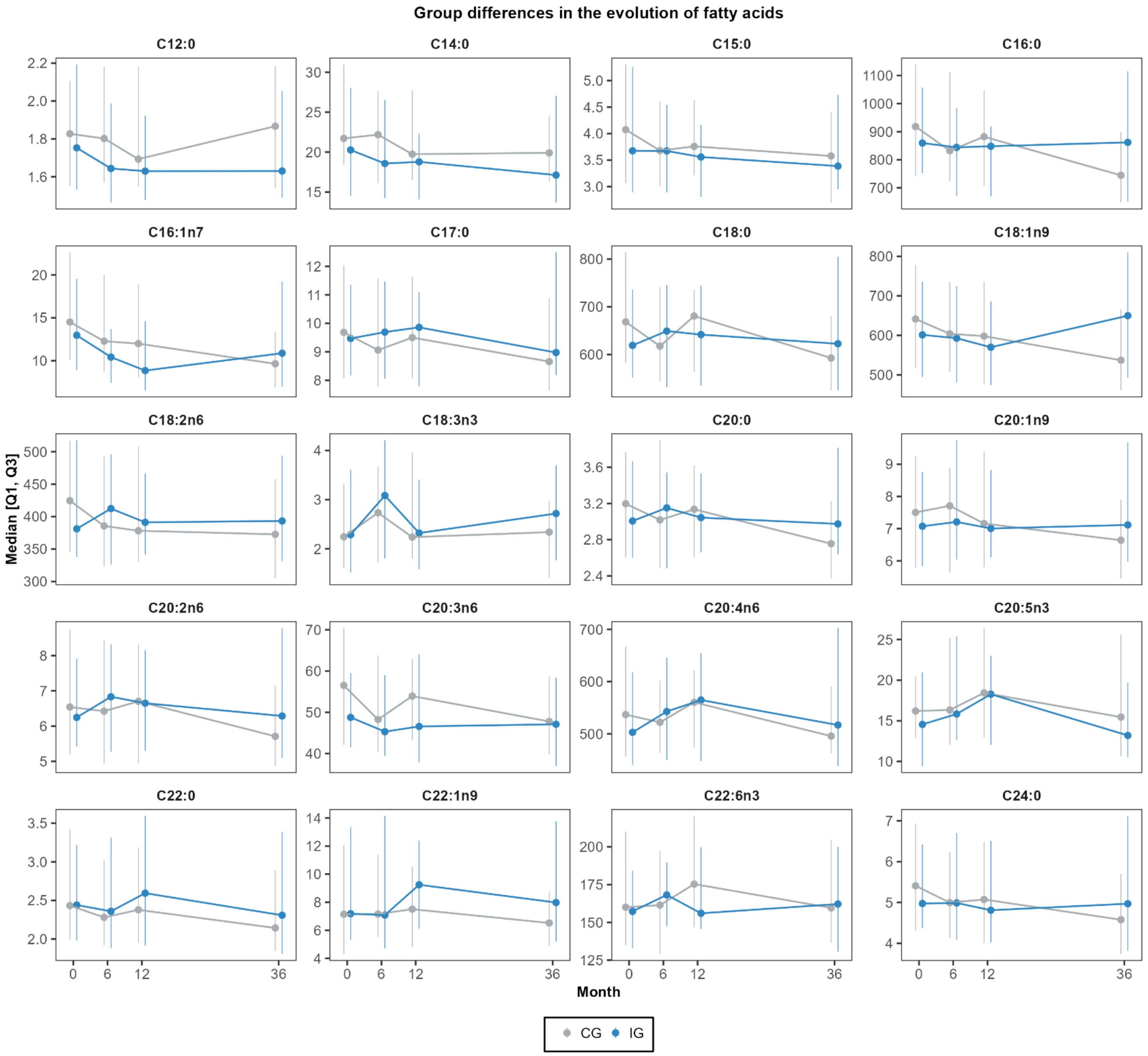

Supplementary Figure 4. Evolution of fatty acids over time by intervention group [N=52 IG and N=53 CG].

Red asterisks (*) indicate significant group differences at each time point (P<0.05). Red hashes (#) indicate significant group differences in the rate of change from baseline (P<0.05). Dependent variables were normalized before estimating linear or linear mixed effects models (when deemed appropriated) using ordered quantile normalization (ORQ) transformation. Models were adjusted by sex, age, and diagnostic of diabetes. IG= intervention group. CG= control group. Q1= first quartile. Q3= third quartile. List of common names for each fatty acid: C12:0= lauric acid. C14:0= myristic acid. C15:0= pentadecylic acid. C16:0= palmitic acid. C16:1n7= palmitoleic acid. C17:0= margaric acid. C18:0= stearic acid. C18:1n9= oleic acid. C18:2n6= linoleic acid (LA). C18:3n3= alpha-linolenic acid (ALA), C20:0= arachidic acid. C20:1n9= *cis*-11-eicosenoic acid. C20:2n6= all *cis*-11,14-eicosadienoic acid. C20:3n6= dihomo-gamma-linolenic acid (DHGLA). C20:4n6= arachidonic acid (ARA). C20:5n3= eicosapentaenoic acid (EPA). C22:0= behenic acid. C22:1n9= erucic acid. C22:6n3= docosahexaenoic acid (DHA). C24:0= lignoceric acid.

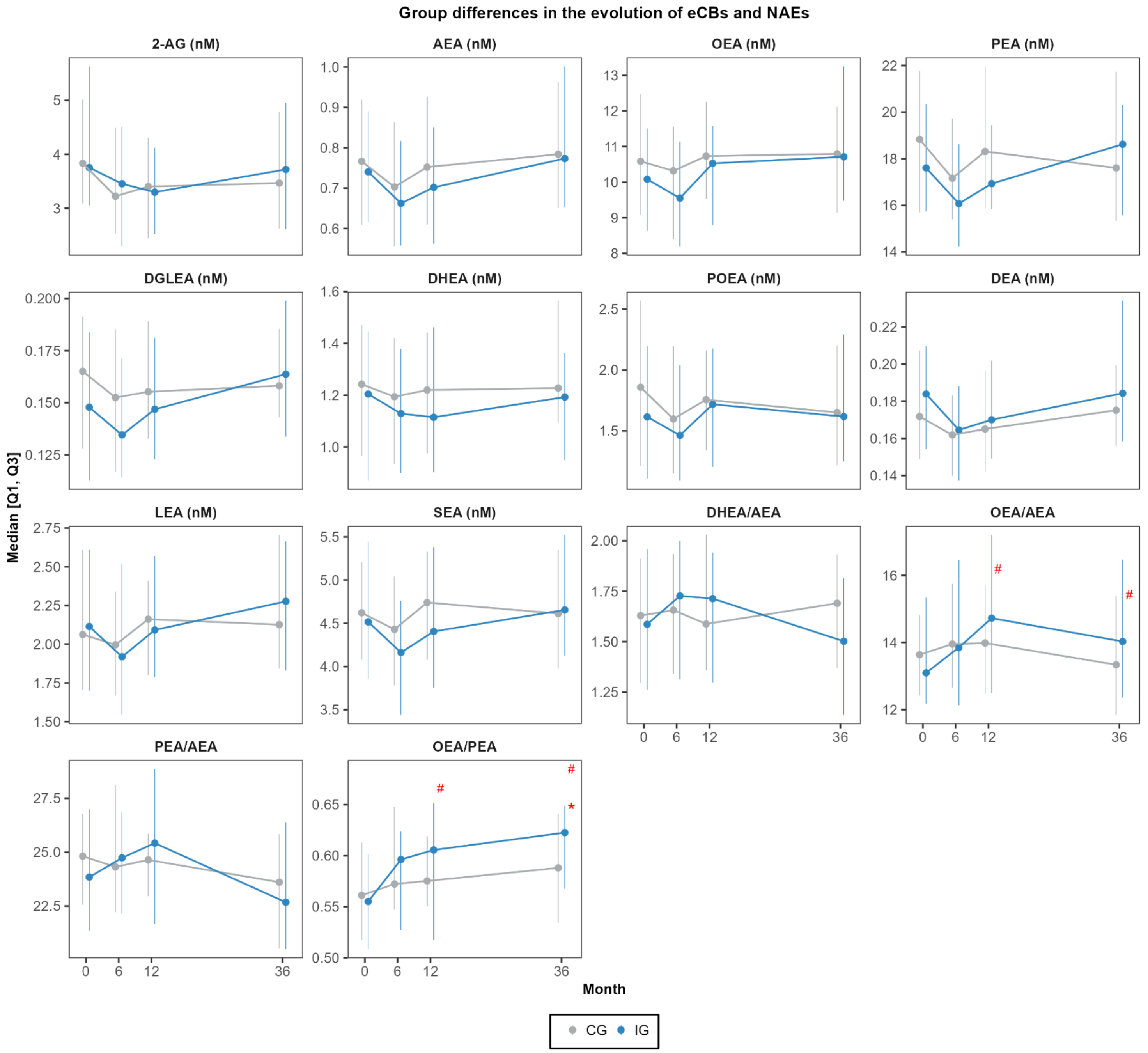

Supplementary Figure 5. Evolution of endocannabinoids and NAEs by intervention group [N=52 IG and N=53 CG].

Red asterisks (*) indicate significant group differences at each time point (P<0.05). Red hashes (#) indicate significant group differences in the rate of change from baseline (P<0.05). Dependent variables were normalized before estimating linear or linear mixed effects models (when deemed appropriated) using ordered quantile normalization (ORQ) transformation. Models were adjusted by sex, age, and diagnostic of diabetes. IG= intervention group. CG= control group. Q1= first quartile. Q3= third quartile. 2-AG= 2-arachidonoylglycerol. AEA= anandamide or *N*-arachidonoyl-ethanolamine. DEA= *N*-docosatetraenoylethanolamine. DGLEA= *N*-dihomo-ɣ-linolenoyl ethanolamide. DHEA= *N*-docosahexaenoylethanolamine. eCBs= endocannabinoids. LEA= *N*-linoleoylethanolamine. OEA= oleoylethanolamide. PEA= palmitoylethanolamide. POEA= *N*-palmitoleoylethanolamine. SEA= *N*-stearoylethanolamine.

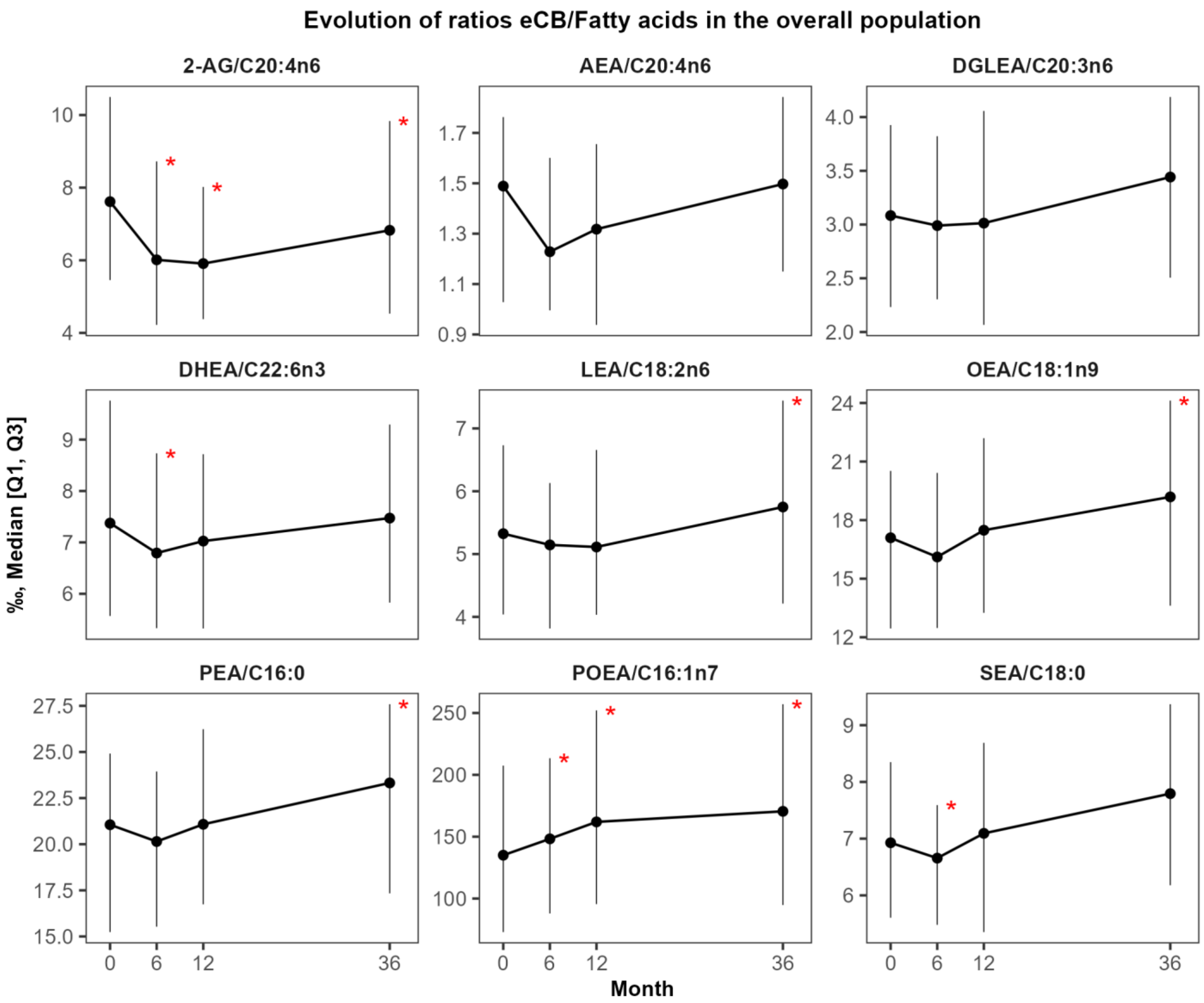

Supplementary Figure 6. Evolution of the ratios eCB/fatty acids over time in the overall population [N=105].

*Indicate significant changes from baseline (P≤0.05). Dependent variables were normalized before estimating linear or linear mixed effects models (when deemed appropriated) using ordered quantile normalization (ORQ) transformation. Models were adjusted by sex, age, intervention group and diagnostic of diabetes. Q1= first quartile. Q3= third quartile. 2-AG= 2-arachidonoylglycerol. AEA= anandamide or *N*-arachidonoyl-ethanolamine. C16:0= palmitic acid. C16:1n7= palmitoleic acid. C18:0= stearic acid. C18:1n9= oleic acid. C18:2n6= linoleic acid (LA). C20:3n6= dihomo-gamma-linolenic acid (DHGLA). C20:4n6= arachidonic acid (ARA). C22:6n3= docosahexaenoic acid (DHA). DGLEA= *N*-dihomo-ɣ-linolenoyl ethanolamide. DHA= docosahexaenoic acid. DHGLA= dihomo-gamma-linolenic acid. LEA= *N*-linoleoylethanolamine. OEA= oleoylethanolamide. PEA= palmitoylethanolamide. POEA= *N*-palmitoleoylethanolamine.

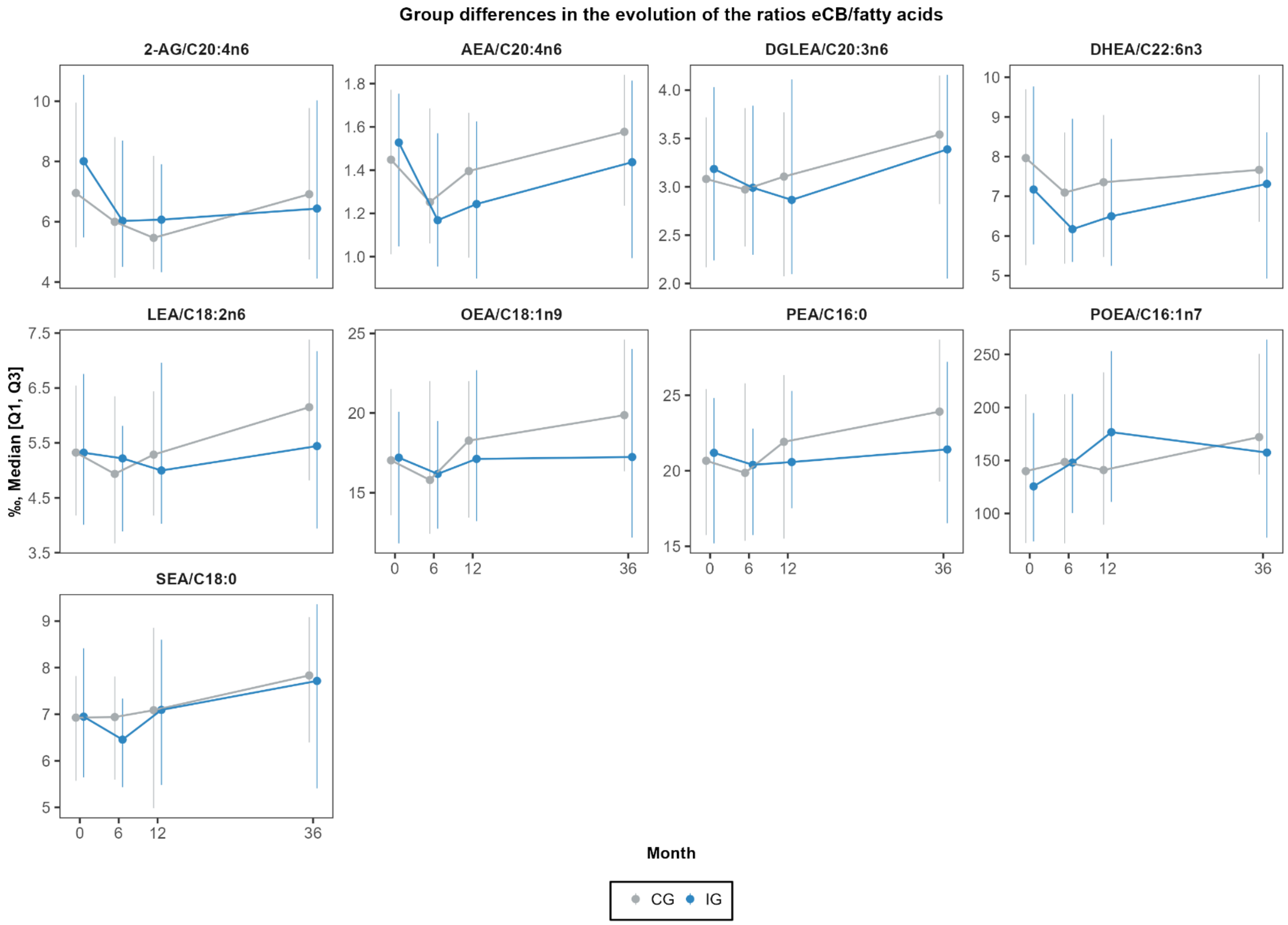

Supplementary Figure 7. Evolution of the ratios eCB/fatty acids over time by intervention group [N=52 IG and N=53 CG].

Red asterisks (*) indicate significant group differences at each time point (P<0.05). Red hashes (#) indicate significant group differences in the rate of change from baseline (P<0.05). Dependent variables were normalized before estimating linear or linear mixed effects models (when deemed appropriated) using ordered quantile normalization (ORQ) transformation. Models were adjusted by sex, age, and diagnostic of diabetes. IG= intervention group. CG= control group. Q1= first quartile. Q3= third quartile. 2-AG= 2-arachidonoylglycerol. AEA= anandamide or *N*-arachidonoyl-ethanolamine. C16:0= palmitic acid. C16:1n7= palmitoleic acid. C18:0= stearic acid. C18:1n9= oleic acid. C18:2n6= linoleic acid (LA). C20:3n6= dihomo-gamma-linolenic acid (DHGLA). C20:4n6= arachidonic acid (ARA). C22:6n3= docosahexaenoic acid (DHA). DGLEA= *N*-dihomo-ɣ-linolenoyl ethanolamide. DHA= docosahexaenoic acid. DHGLA= dihomo-gamma-linolenic acid. LEA= *N*-linoleoylethanolamine. OEA= oleoylethanolamide. PEA= palmitoylethanolamide. POEA= *N*-palmitoleoylethanolamine.

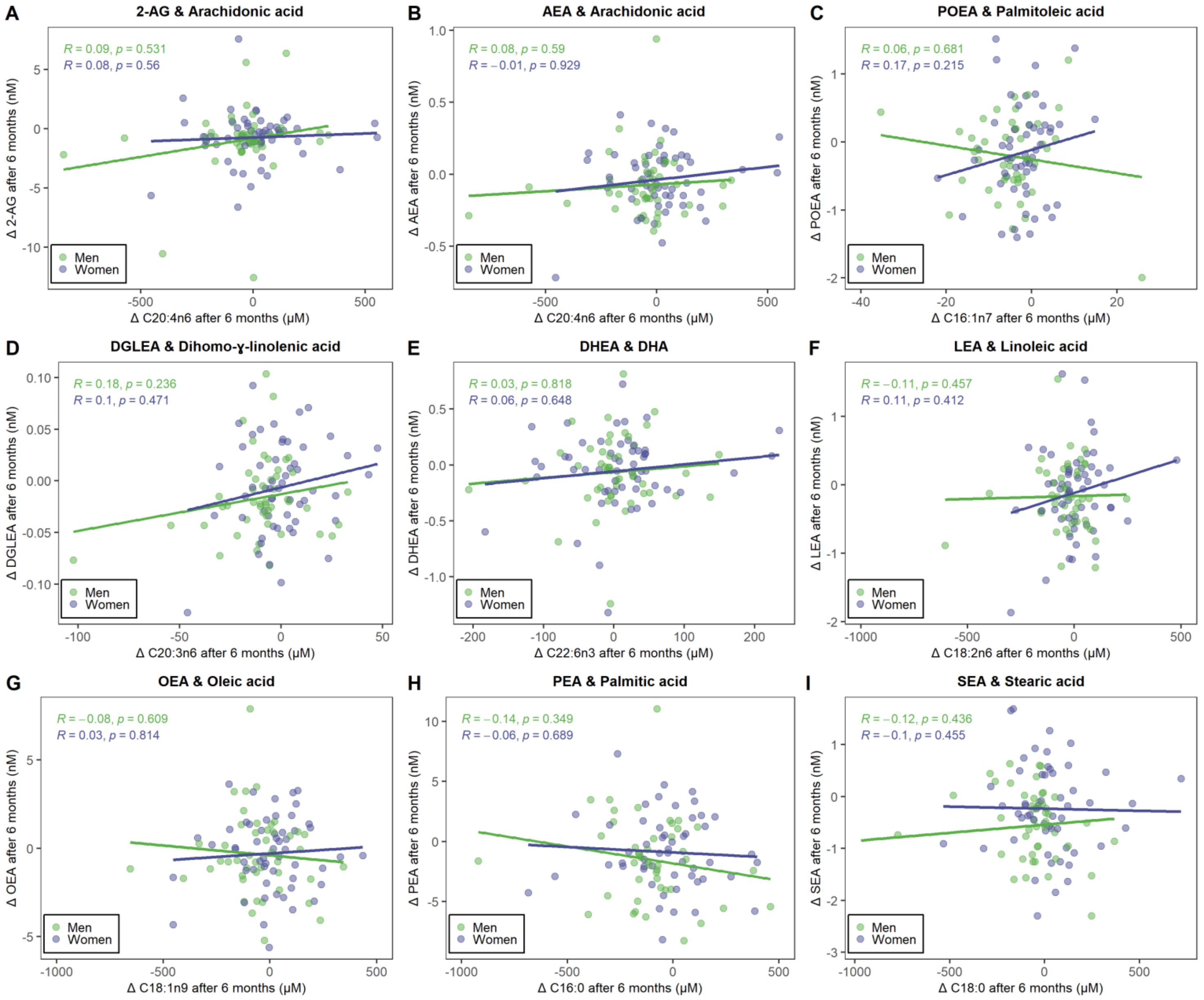

Supplementary Figure 8. Scatter plots showing the correlations between change in eCBs or NAEs after 6 months and the respective change in their precursor fatty acids in men and women.

Values indicate the Spearman's correlation coefficient and associated P-value. Δ= difference between 6 months and baseline values. 2-AG= 2-arachidonoylglycerol. AEA= anandamide or *N*-arachidonoyl-ethanolamine. C16:0= palmitic acid. C16:1n7= palmitoleic acid. C18:0= stearic acid. C18:1n9= oleic acid. C18:2n6= linoleic acid (LA). C20:3n6= dihomo-gamma-linolenic acid (DHGLA). C20:4n6= arachidonic acid (ARA). C22:6n3= docosahexaenoic acid (DHA). DGLEA= *N*-dihomo-ɣ-linolenoyl ethanolamide. DHA= docosahexaenoic acid. DHGLA= dihomo-gamma-linolenic acid. LEA= *N*-linoleoylethanolamine. OEA= oleoylethanolamide. PEA= palmitoylethanolamide. POEA= *N*-palmitoleoylethanolamine.

Supplementary Tables

Supplementary Table 1. Change in anthropometric, cardiovascular and glycemic factors from baseline to 6 months, 1 year and 3 years by sex

		Men [N=48]					Women [N=57]					Sex differences			
			Values at each time point		Change from baseline			Values at each time point		Change from baseline		At each time point		In change over time	
Variable	Time	Missing [N (%)]	Mean (SD)	Median (Q1, Q3)	Mean change (SD)	Cohen's d (size)[1]	Missing [N (%)]	Mean (SD)	Median (Q1, Q3)	Mean change (SD)	Cohen's d (size)[1]	Cohen's d (size)[1]	P-value*	Cohen's d (size)[1]	P-value*
Weight (kg)	Baseline	0 (0.0)	96.2 (13.2)	98.0 (87.3, 103.5)	Ref		0 (0.0)	83.9 (13.0)	84.1 (75.0, 92.5)	Ref	Ref	-0.95 (L)	**<0.001**	Ref	
	6 months	0 (0.0)	88.3 (12.8)	87.2 (80.8, 94.1)	-7.9 (5.5)	-0.61 (M)	0 (0.0)	79.0 (12.6)	77.0 (70.7, 87.0)	-4.9 (3.6)	-0.38 (S)	-0.74 (M)	**<0.001**	0.65 (M)	**0.003**
	1 year	0 (0.0)	88.6 (13.4)	88.3 (79.7, 94.8)	-7.6 (6.1)	-0.58 (M)	0 (0.0)	79.1 (13.0)	77.2 (70.2, 87.6)	-4.8 (4.2)	-0.37 (S)	-0.72 (M)	**0.001**	0.55 (M)	**0.016**
	3 years	2 (4.2)	89.0 (14.3)	87.1 (78.7, 96.6)	-7.5 (6.5)	-0.53 (M)	2 (3.5)	79.2 (13.7)	79.0 (68.5, 86.3)	-4.9 (5.1)	-0.35 (S)	-0.70 (M)	**0.002**	0.45 (S)	0.062
BMI (kg/m^2)	Baseline	0 (0.0)	33.5 (3.4)	33.2 (30.8, 36.0)	Ref		0 (0.0)	34.0 (3.5)	33.9 (31.6, 36.8)	Ref	Ref	0.15 (S)	0.218	Ref	
	6 months	0 (0.0)	30.7 (3.6)	30.3 (27.9, 33.4)	-2.7 (2.0)	-0.78 (L)	0 (0.0)	32.0 (3.5)	31.9 (29.4, 34.8)	-2.0 (1.4)	-0.57 (M)	0.36 (S)	**0.023**	0.44 (S)	**0.015**
	1 year	0 (0.0)	30.8 (3.7)	30.5 (28.0, 33.1)	-2.6 (2.2)	-0.74 (M)	0 (0.0)	32.0 (3.7)	32.1 (29.1, 34.9)	-1.9 (1.7)	-0.55 (M)	0.33 (S)	**0.035**	0.36 (S)	0.062
	3 years	2 (4.2)	30.9 (3.7)	30.1 (28.3, 33.1)	-2.7 (2.3)	-0.73 (M)	2 (3.5)	32.0 (3.9)	31.6 (28.9, 35.1)	-2.0 (2.0)	-0.55 (M)	0.28 (S)	0.066	0.30 (S)	0.115
Waist (cm)	Baseline	0 (0.0)	115.7 (8.5)	114.5 (109.4, 120.6)	Ref		0 (0.0)	108.6 (10.0)	108.0 (102.5, 114.5)	Ref	Ref	-0.76 (L)	**<0.001**	Ref	
	6 months	0 (0.0)	108.0 (9.0)	107.5 (100.0, 114.1)	-7.7 (5.2)	-0.88 (L)	0 (0.0)	104.1 (9.7)	103.5 (97.0, 109.0)	-4.5 (4.0)	-0.46 (M)	-0.42 (S)	0.072	0.70 (M)	**0.001**
	1 year	3 (6.2)	107.6 (9.1)	106.0 (100.0, 113.0)	-7.8 (6.1)	-0.92 (L)	1 (1.8)	104.1 (10.8)	103.2 (96.8, 111.2)	-4.5 (4.6)	-0.43 (S)	-0.35 (S)	0.145	0.61 (M)	**0.007**
	3 years	8 (16.7)	107.9 (9.5)	107.0 (101.1, 113.1)	-7.7 (7.1)	-0.87 (L)	8 (14.0)	104.9 (11.2)	104.0 (97.5, 112.7)	-4.1 (5.4)	-0.35 (S)	-0.29 (S)	0.250	0.58 (M)	**0.008**
Hip (cm)	Baseline	0 (0.0)	111.8 (7.0)	111.0 (106.5, 116.2)	Ref		0 (0.0)	116.5 (9.7)	118.0 (107.0, 124.0)	Ref	Ref	0.55 (M)	**0.002**	Ref	
	6 months	0 (0.0)	107.3 (6.3)	107.0 (103.4, 110.0)	-4.5 (4.1)	-0.68 (M)	0 (0.0)	113.3 (9.5)	113.0 (105.5, 121.0)	-3.3 (3.2)	-0.34 (S)	0.73 (M)	**<0.001**	0.35 (S)	**0.037**
	1 year	3 (6.2)	107.1 (6.7)	106.5 (103.0, 110.0)	-4.8 (4.6)	-0.68 (M)	1 (1.8)	113.6 (9.7)	113.5 (105.8, 121.0)	-2.9 (4.3)	-0.30 (S)	0.76 (L)	**<0.001**	0.41 (S)	**0.027**
	3 years	8 (16.7)	106.7 (6.3)	106.0 (102.8, 111.0)	-5.0 (5.6)	-0.76 (L)	8 (14.0)	113.7 (9.9)	112.5 (106.0, 122.0)	-3.2 (4.5)	-0.29 (S)	0.82 (L)	**<0.001**	0.36 (S)	**0.041**
Systolic blood pressure (mmHg)	Baseline	0 (0.0)	145.8 (12.9)	145.5 (137.0, 154.2)	Ref		0 (0.0)	145.5 (14.5)	144.0 (137.0, 153.0)	Ref	Ref	-0.02 (VS)	0.805	Ref	
	6 months	2 (4.2)	139.8 (16.0)	138.5 (130.0, 146.8)	-6.2 (12.9)	-0.41 (S)	1 (1.8)	139.0 (16.9)	137.0 (130.0, 146.2)	-6.4 (16.4)	-0.41 (S)	-0.04 (VS)	0.755	-0.01 (VS)	0.907
	1 year	4 (8.3)	138.6 (14.2)	137.0 (128.8, 148.5)	-5.7 (14.7)	-0.53 (M)	1 (1.8)	140.8 (16.0)	138.0 (130.8, 147.0)	-4.8 (15.3)	-0.30 (S)	0.14 (VS)	0.585	0.06 (VS)	0.444
	3 years	4 (8.3)	136.9 (16.1)	139.0 (128.5, 146.2)	-8.4 (15.1)	-0.61 (M)	4 (7.0)	138.7 (17.5)	139.0 (128.0, 147.0)	-7.1 (17.1)	-0.43 (S)	0.10 (VS)	0.741	0.08 (VS)	0.488
Diastolic blood pressure (mmHg)	Baseline	0 (0.0)	80.3 (9.8)	81.0 (75.0, 87.0)	Ref		0 (0.0)	75.7 (10.5)	77.0 (68.0, 83.0)	Ref	Ref	-0.45 (S)	**0.036**	Ref	
	6 months	2 (4.2)	76.0 (11.0)	77.5 (67.2, 85.0)	-4.6 (10.7)	-0.41 (S)	1 (1.8)	70.7 (10.8)	69.0 (64.0, 75.2)	-4.8 (12.4)	-0.47 (M)	-0.48 (M)	**0.017**	-0.02 (VS)	0.925
	1 year	4 (8.3)	77.8 (10.1)	79.0 (69.0, 87.0)	-2.4 (11.4)	-0.25 (S)	1 (1.8)	69.8 (11.5)	68.0 (63.0, 75.5)	-6.0 (10.6)	-0.53 (M)	-0.73 (M)	**<0.001**	-0.33 (S)	0.114
	3 years	4 (8.3)	75.8 (11.0)	76.0 (69.8, 83.2)	-4.3 (10.8)	-0.44 (S)	4 (7.0)	70.5 (12.1)	70.0 (62.0, 77.0)	-5.5 (13.0)	-0.46 (M)	-0.45 (M)	**0.044**	-0.10 (VS)	0.775
HbA1c (%)	Baseline	0 (0.0)	6.4 (1.0)	6.1 (5.7, 7.0)	Ref		0 (0.0)	6.3 (0.9)	6.0 (5.8, 6.6)	Ref	Ref	-0.16 (S)	0.830	Ref	
	6 months	0 (0.0)	5.9 (0.9)	5.7 (5.4, 6.3)	-0.5 (0.5)	-0.53 (M)	0 (0.0)	6.0 (0.9)	5.8 (5.6, 6.3)	-0.3 (0.5)	-0.28 (S)	0.10 (VS)	0.138	0.45 (M)	**0.004**
	1 year	5 (10.4)	6.0 (0.8)	5.8 (5.4, 6.3)	-0.4 (0.6)	-0.46 (M)	1 (1.8)	6.1 (0.8)	5.9 (5.6, 6.4)	-0.2 (0.5)	-0.23 (S)	0.09 (VS)	0.112	0.41 (S)	**0.004**
	3 years	8 (16.7)	6.0 (0.8)	5.8 (5.4, 6.4)	-0.3 (0.6)	-0.43 (S)	7 (12.3)	6.2 (1.0)	5.9 (5.6, 6.5)	-0.1 (0.6)	-0.06 (VS)	0.21 (S)	0.029	0.32 (S)	**0.037**
Glucose (mg/dL)	Baseline	0 (0.0)	125.7 (33.2)	113.0 (103.0, 140.0)	Ref		0 (0.0)	116.9 (28.1)	111.0 (100.0, 130.0)	Ref	Ref	-0.29 (S)	0.535	Ref	
	6 months	0 (0.0)	114.8 (36.0)	102.5 (94.0, 123.5)	-10.9 (19.6)	-0.31 (S)	0 (0.0)	111.5 (26.2)	105.0 (97.0, 115.0)	-5.5 (14.9)	-0.20 (S)	-0.11 (VS)	0.780	0.31 (S)	**0.046**
	1 year	5 (10.4)	112.7 (21.7)	107.0 (97.5, 123.0)	-10.4 (14.7)	-0.46 (M)	1 (1.8)	113.8 (25.8)	106.0 (98.0, 119.0)	-3.5 (17.0)	-0.12 (VS)	0.05 (VS)	0.125	0.44 (S)	0.065
	3 years	8 (16.7)	112.1 (20.5)	107.0 (97.2, 128.0)	-9.4 (20.9)	-0.48 (M)	7 (12.3)	120.3 (29.5)	110.0 (100.0, 137.5)	1.9 (23.8)	0.12 (VS)	0.32 (S)	**0.011**	0.50 (M)	**0.012**
Insulin (µUI/mL)	Baseline	0 (0.0)	9.1 (4.7)	7.9 (5.8, 12.2)	Ref		0 (0.0)	8.7 (5.2)	7.0 (5.8, 10.3)	Ref	Ref	-0.08 (VS)	0.814	Ref	
	6 months	0 (0.0)	7.3 (4.0)	6.1 (4.9, 9.7)	-1.8 (3.1)	-0.41 (S)	0 (0.0)	7.4 (4.2)	7.2 (4.3, 8.7)	-1.3 (3.4)	-0.28 (S)	0.02 (VS)	0.719	0.15 (VS)	0.388
	1 year	5 (10.4)	7.2 (3.9)	6.7 (4.1, 9.7)	-2.4 (3.8)	-0.45 (M)	1 (1.8)	7.6 (4.1)	6.8 (4.6, 10.2)	-0.9 (3.5)	-0.24 (S)	0.11 (VS)	0.337	0.40 (S)	0.056
	3 years	8 (16.7)	6.2 (3.3)	5.6 (3.5, 8.4)	-3.4 (3.6)	-0.71 (M)	7 (12.3)	7.1 (4.4)	6.0 (3.9, 8.7)	-1.8 (3.5)	-0.34 (S)	0.23 (S)	0.272	0.45 (S)	**0.029**
HOMA-IR	Baseline	0 (0.0)	2.9 (1.9)	2.4 (1.4, 3.7)	Ref		0 (0.0)	2.6 (1.8)	1.9 (1.5, 3.0)	Ref		-0.20 (S)	0.575	Ref	
	6 months	0 (0.0)	2.2 (1.7)	1.8 (1.2, 2.6)	-0.7 (1.2)	-0.41 (S)	0 (0.0)	2.1 (1.3)	1.8 (1.1, 2.8)	-0.5 (1.3)	-0.30 (S)	-0.08 (VS)	0.887	0.21 (S)	0.225
	1 year	5 (10.4)	2.1 (1.4)	1.9 (1.0, 2.5)	-0.9 (1.3)	-0.49 (M)	1 (1.8)	2.2 (1.6)	1.9 (1.1, 2.9)	-0.3 (1.2)	-0.19 (S)	0.10 (VS)	0.233	0.52 (M)	**0.030**
	3 years	8 (16.7)	1.8 (1.1)	1.5 (0.9, 2.6)	-1.2 (1.4)	-0.73 (M)	7 (12.3)	2.2 (1.9)	1.7 (1.1, 2.9)	-0.4 (1.5)	-0.18 (S)	0.30 (S)	0.087	0.56 (M)	**0.008**
HDL-c (mg/dL)	Baseline	0 (0.0)	47.2 (7.9)	47.0 (42.0, 51.2)	Ref		0 (0.0)	58.0 (9.5)	56.0 (52.0, 65.0)	Ref		1.24 (VL)	**<0.001**	Ref	
	6 months	0 (0.0)	51.0 (8.6)	50.5 (43.8, 59.0)	3.8 (6.5)	0.46 (M)	0 (0.0)	60.4 (10.1)	60.0 (54.0, 70.0)	2.3 (7.6)	0.24 (S)	1.00 (L)	**<0.001**	-0.21 (S)	0.264
	1 year	5 (10.4)	51.7 (9.2)	51.0 (45.0, 57.5)	4.4 (7.7)	0.54 (M)	1 (1.8)	59.9 (8.7)	60.0 (53.8, 65.2)	1.8 (6.9)	0.20 (S)	0.91 (L)	**<0.001**	-0.36 (S)	0.089
	3 years	8 (16.7)	49.5 (10.5)	48.5 (41.5, 57.0)	2.0 (7.6)	0.26 (S)	7 (12.3)	58.4 (10.2)	57.0 (52.0, 67.2)	0.0 (6.8)	0.04 (VS)	0.86 (L)	**<0.001**	-0.29 (S)	0.190
LDL-c (mg/dL)	Baseline	3 (6.2)	134.4 (35.5)	135.0 (109.0, 159.0)	Ref		3 (5.3)	155.9 (32.6)	152.0 (134.5, 178.5)	Ref		0.63 (M)	**0.004**	Ref	
	6 months	1 (2.1)	123.0 (34.1)	116.0 (97.0, 145.5)	-12.2 (31.6)	-0.33 (S)	2 (3.5)	141.9 (32.0)	138.0 (120.0, 160.0)	-12.9 (23.5)	-0.44 (S)	0.57 (M)	**0.004**	-0.02 (VS)	0.981
	1 year	7 (14.6)	136.5 (34.9)	137.0 (110.0, 156.0)	0.7 (39.8)	0.06 (VS)	1 (1.8)	150.2 (33.1)	151.0 (125.5, 170.0)	-3.0 (32.2)	-0.17 (S)	0.40 (S)	**0.078**	-0.10 (VS)	0.434
	3 years	9 (18.8)	123.3 (35.4)	125.0 (97.5, 140.5)	-14.1 (47.2)	-0.31 (S)	7 (12.3)	139.4 (33.4)	138.0 (111.2, 152.8)	-16.3 (38.3)	-0.50 (M)	0.47 (M)	**0.052**	-0.05 (VS)	0.726
Cholesterol (mg/dL)	Baseline	0 (0.0)	213.0 (37.7)	215.0 (189.0, 237.2)	Ref		0 (0.0)	241.4 (39.4)	232.0 (216.0, 272.0)	Ref		0.73 (M)	**<0.001**	Ref	
	6 months	0 (0.0)	199.8 (36.0)	188.5 (170.5, 232.5)	-13.2 (35.8)	-0.36 (S)	0 (0.0)	230.7 (39.5)	227.0 (204.0, 252.0)	-10.7 (29.0)	-0.27 (S)	0.81 (L)	**<0.001**	0.08 (VS)	0.617

	1 year	5 (10.4)	215.5 (40.0)	222.0 (183.0, 241.0)	2.0 (45.1)	0.06 (VS)	1 (1.8)	237.4 (37.4)	235.0 (210.8, 259.5)	-2.8 (34.5)	-0.10 (VS)	0.57 (M)	**0.008**	-0.12 (VS)	0.480
	3 years	8 (16.7)	194.9 (40.0)	201.0 (166.2, 224.8)	-17.6 (52.3)	-0.47 (M)	7 (12.3)	224.6 (38.7)	218.0 (193.2, 242.8)	-17.1 (41.4)	-0.43 (S)	0.76 (L)	**0.001**	0.01 (VS)	0.831
	Baseline	0 (0.0)	173.9 (110.1)	147.0 (108.8, 210.8)	Ref		0 (0.0)	148.1 (66.4)	130.0 (100.0, 185.0)	Ref		-0.29 (S)	0.207	Ref	
Triglycerides	6 months	0 (0.0)	134.0 (65.9)	116.0 (88.8, 159.5)	-39.8 (69.7)	-0.44 (S)	0 (0.0)	134.3 (59.0)	119.0 (96.0, 155.0)	-13.8 (51.2)	-0.22 (S)	0.00 (VS)	0.928	0.43 (S)	**0.038**
(mg/dL)	1 year	5 (10.4)	131.4 (78.4)	109.0 (76.5, 166.0)	-30.6 (65.6)	-0.44 (S)	1 (1.8)	136.5 (51.5)	123.0 (100.2, 173.2)	-11.4 (41.1)	-0.19 (S)	0.08 (VS)	0.710	0.36 (S)	**0.005**
	3 years	8 (16.7)	117.2 (58.8)	110.0 (65.0, 147.5)	-46.1 (76.1)	-0.63 (M)	7 (12.3)	134.3 (51.1)	131.0 (100.5, 159.8)	-14.5 (55.8)	-0.23 (S)	0.31 (S)	0.145	0.48 (M)	**0.005**

Positive/negative values for 6 months, 1 year and 3 years change indicate increase and decrease, respectively, compared to the baseline value.

P-values of cross-sectional differences were obtained from the analysis of variance (ANOVA) from multivariable-adjusted linear models. P-values of differences in the rate of change were obtained from linear mixed effects models. All the analyses were adjusted by intervention group, age, and diagnostic of type 2 diabetes. Dependent variables were normalized before estimating p-values, using ordered quantile normalization (ORQ) transformation.

N=number. 95%CI= 95% confidence intervals

[1]Effect Size: VS = very small (Cohen's d < 0.2); S = small [Cohen's d (0.2–0.5)]; M = medium [Cohen's d (0.5–0.8)]; L = large [Cohen's d (0.8–1.2)]; VL = very large (Cohen's d ≥ 1.2).

Bold values denote statistical significance at P<0.05.

Supplementary Table 2. Change in intervention adherence and food consumption from baseline to 6 months, 1 year and 3 years by sex

		Men [N=48]					Women [N=57]					Sex differences			
			Values at each time point		Change from baseline			Values at each time point		Change from baseline		At each time point		In change over time	
Variable	Time	Missing [N (%)]	Mean (SD)	Median (Q1, Q3)	Mean change (SD)	Cohen's d (size)[1]	Missing [N (%)]	Mean (SD)	Median (Q1, Q3)	Mean change (SD)	Cohen's d (size)[1]	Cohen's d (size)[1]	P-value*	Cohen's d (size)[1]	P-value*
er-MedDiet adherence	Baseline	0 (0.0)	6.8 (2.7)	6.0 (5.0, 9.0)	Ref		0 (0.0)	7.8 (2.4)	8.0 (6.0, 9.0)	Ref		0.43 (S)	0.058		
	6 months	0 (0.0)	11.3 (2.7)	11.5 (9.0, 13.0)	4.5 (3.1)	1.67 (VL)	0 (0.0)	11.4 (2.7)	12.0 (10.0, 13.0)	3.6 (3.1)	1.40 (VL)	0.06 (VS)	0.582	-0.29 (S)	0.109
	1 year	0 (0.0)	11.1 (3.0)	11.0 (9.0, 14.0)	4.3 (3.4)	1.50 (VL)	0 (0.0)	11.0 (2.6)	11.0 (9.0, 13.0)	3.2 (3.2)	1.26 (VL)	-0.02 (VS)	0.922	-0.34 (S)	0.065
	3 years	8 (16.7)	10.7 (3.0)	10.0 (8.0, 13.0)	3.9 (2.9)	1.37 (VL)	6 (10.5)	10.4 (2.7)	11.0 (8.5, 12.0)	2.5 (2.9)	1.00 (L)	-0.11 (VS)	0.556	-0.48 (M)	**0.017**
Activity (MET min/week)	Baseline	0 (0.0)	2735.8 (2382.1)	1958.0 (902.1, 4666.7)	Ref		0 (0.0)	2269.5 (1971.7)	1657.3 (895.1, 3076.9)	Ref		-0.21 (S)	0.189		
	6 months	0 (0.0)	3376.5 (2104.8)	2849.7 (1930.1, 4206.3)	640.7 (2149.9)	0.29 (S)	0 (0.0)	2905.6 (2029.0)	2237.8 (1405.8, 4055.9)	636.1 (2004.3)	0.32 (S)	-0.23 (S)	0.240	0.00 (VS)	0.554
	1 year	0 (0.0)	3219.2 (2059.4)	2744.8 (1304.2, 4737.8)	483.5 (2493.3)	0.22 (S)	0 (0.0)	3022.2 (2295.9)	2545.4 (1538.5, 4055.9)	752.6 (1621.4)	0.35 (S)	-0.09 (VS)	0.513	0.13 (VS)	0.981
	3 years	8 (16.7)	3304.5 (2238.5)	2944.1 (1637.5, 4821.7)	479.2 (3094.4)	0.25 (S)	6 (10.5)	2471.3 (2028.8)	1958.0 (1039.6, 3356.6)	149.8 (1899.4)	0.10 (VS)	-0.39 (S)	**0.045**	-0.13 (VS)	0.336
Total energy intake (Kcal/day)	Baseline	0 (0.0)	2510.5 (711.1)	2439.8 (1991.7, 2844.1)	Ref		0 (0.0)	2169.3 (465.9)	2150.5 (1895.3, 2408.7)	Ref		-0.58 (M)	**0.005**		
	6 months	0 (0.0)	2304.5 (327.3)	2246.3 (2140.1, 2481.6)	-206.0 (751.1)	-0.37 (S)	0 (0.0)	2190.8 (348.6)	2188.8 (1986.2, 2419.3)	21.5 (444.0)	0.05 (VS)	-0.34 (S)	0.104	0.38 (S)	0.151
	1 year	2 (4.2)	2428.2 (371.4)	2413.8 (2201.8, 2579.8)	-78.4 (715.6)	-0.14 (VS)	1 (1.8)	2274.3 (400.7)	2226.4 (1970.1, 2445.9)	112.8 (483.2)	0.24 (S)	-0.40 (S)	**0.049**	0.32 (S)	0.318
	3 years	8 (16.7)	2200.2 (392.6)	2094.5 (1979.5, 2449.5)	-289.3 (757.7)	-0.53 (M)	7 (12.3)	2043.6 (383.2)	2044.7 (1815.6, 2202.9)	-152.7 (545.8)	-0.29 (S)	-0.40 (S)	0.055	0.21 (S)	0.451
EVOO (g/day)	Baseline	0 (0.0)	37.2 (17.5)	50.0 (25.0, 50.0)	Ref		0 (0.0)	33.6 (20.0)	50.0 (10.0, 50.0)	Ref		-0.19 (S)	0.347		
	6 months	0 (0.0)	46.8 (9.5)	50.0 (50.0, 50.0)	9.6 (16.7)	0.68 (M)	0 (0.0)	44.7 (10.3)	50.0 (50.0, 50.0)	11.2 (22.8)	0.70 (M)	-0.20 (S)	0.177	0.08 (VS)	0.830
	1 year	2 (4.2)	46.6 (9.7)	50.0 (50.0, 50.0)	9.5 (18.9)	0.66 (M)	1 (1.8)	47.3 (11.8)	50.0 (50.0, 50.0)	14.0 (21.7)	0.83 (L)	0.06 (VS)	0.867	0.22 (S)	0.249
	3 years	8 (16.7)	41.6 (14.2)	50.0 (25.0, 50.0)	4.7 (23.1)	0.27 (S)	7 (12.3)	38.5 (14.4)	50.0 (25.0, 50.0)	5.8 (25.7)	0.28 (S)	-0.21 (S)	0.242	0.04 (VS)	0.928
Olive oil (g/day)	Baseline	0 (0.0)	45.6 (12.5)	50.0 (50.0, 50.0)	Ref		0 (0.0)	45.9 (14.2)	50.0 (50.0, 50.0)	Ref		0.02 (VS)	0.917		
	6 months	0 (0.0)	48.9 (9.0)	50.0 (50.0, 50.0)	3.3 (14.8)	0.31 (S)	0 (0.0)	46.5 (9.9)	50.0 (50.0, 50.0)	0.6 (18.8)	0.05 (VS)	-0.26 (S)	0.166	-0.16 (S)	0.444
	1 year	2 (4.2)	51.0 (9.8)	50.0 (50.0, 53.4)	5.1 (14.4)	0.48 (M)	1 (1.8)	49.6 (8.6)	50.0 (50.0, 50.0)	3.8 (17.2)	0.32 (S)	-0.16 (S)	0.456	-0.08 (VS)	0.297
	3 years	8 (16.7)	47.0 (12.5)	50.0 (46.2, 54.3)	0.2 (18.0)	0.11 (VS)	7 (12.3)	43.9 (13.9)	50.0 (29.3, 53.6)	-2.3 (20.5)	-0.14 (VS)	-0.23 (S)	0.252	-0.13 (VS)	0.300
Walnuts (g/day)	Baseline	0 (0.0)	7.5 (9.8)	3.1 (0.0, 12.9)	Ref		0 (0.0)	8.4 (8.9)	4.3 (2.0, 12.9)	Ref		0.09 (VS)	0.764		
	6 months	0 (0.0)	20.2 (9.0)	23.6 (12.9, 30.0)	12.7 (11.8)	1.35 (VL)	0 (0.0)	18.9 (9.7)	23.6 (12.9, 30.0)	10.5 (13.4)	1.13 (L)	-0.14 (VS)	0.590	-0.17 (S)	0.352
	1 year	2 (4.2)	18.0 (10.7)	23.6 (12.9, 30.0)	10.4 (14.4)	1.02 (L)	1 (1.8)	17.6 (10.3)	23.6 (12.9, 23.6)	9.6 (12.4)	0.96 (L)	-0.04 (VS)	0.866	-0.06 (VS)	0.479
	3 years	8 (16.7)	14.4 (13.4)	12.9 (4.3, 12.9)	6.2 (13.2)	0.59 (M)	7 (12.3)	11.0 (8.3)	12.9 (4.3, 12.9)	2.4 (11.9)	0.30 (S)	-0.31 (S)	0.143	-0.31 (S)	0.095
Nuts (g/day)	Baseline	0 (0.0)	15.0 (14.9)	10.7 (4.0, 25.7)	Ref		0 (0.0)	16.5 (16.3)	12.9 (4.0, 25.7)	Ref		0.09 (VS)	0.673		
	6 months	0 (0.0)	42.1 (21.2)	45.0 (30.0, 53.6)	27.1 (23.8)	1.48 (VL)	0 (0.0)	43.3 (20.6)	38.6 (30.0, 53.6)	26.8 (24.5)	1.44 (VL)	0.06 (VS)	0.727	-0.01 (VS)	0.814
	1 year	2 (4.2)	37.8 (21.8)	37.5 (26.2, 48.7)	22.6 (27.6)	1.23 (VL)	1 (1.8)	41.1 (21.8)	38.6 (25.7, 51.7)	24.9 (24.6)	1.28 (VL)	0.15 (VS)	0.528	0.09 (VS)	0.693
	3 years	8 (16.7)	23.6 (15.5)	23.5 (12.8, 30.0)	8.8 (16.9)	0.57 (M)	7 (12.3)	23.2 (14.1)	24.6 (12.9, 32.0)	5.9 (19.5)	0.44 (S)	-0.03 (VS)	0.817	-0.16 (S)	0.680
Fatty fish (g/day)	Baseline	0 (0.0)	38.2 (23.6)	27.1 (20.9, 59.9)	Ref		0 (0.0)	36.5 (24.7)	25.7 (18.6, 59.0)	Ref		-0.07 (VS)	0.659		
	6 months	0 (0.0)	49.9 (21.1)	59.0 (25.7, 62.8)	11.7 (30.0)	0.52 (M)	0 (0.0)	46.1 (23.2)	59.0 (25.7, 62.8)	9.6 (32.9)	0.40 (S)	-0.17 (S)	0.332	-0.07 (VS)	0.688
	1 year	2 (4.2)	51.5 (23.7)	59.0 (32.6, 62.8)	13.8 (29.4)	0.56 (M)	1 (1.8)	46.5 (22.8)	59.0 (25.7, 62.8)	10.4 (31.9)	0.42 (S)	-0.22 (S)	0.180	-0.11 (VS)	0.550
	3 years	8 (16.7)	44.6 (24.3)	49.5 (21.4, 62.8)	7.8 (30.6)	0.27 (S)	7 (12.3)	50.7 (21.7)	59.0 (30.1, 62.8)	15.3 (30.2)	0.61 (M)	0.27 (S)	0.290	0.25 (S)	0.224
Fish (g/day)	Baseline	0 (0.0)	112.7 (43.5)	117.3 (76.6, 149.9)	Ref		0 (0.0)	116.8 (44.7)	121.9 (77.8, 145.2)	Ref		0.09 (VS)	0.700		
	6 months	0 (0.0)	131.7 (43.2)	132.9 (92.9, 165.7)	19.0 (60.8)	0.44 (S)	0 (0.0)	133.4 (42.1)	140.4 (107.3, 165.9)	16.6 (53.7)	0.38 (S)	0.04 (VS)	0.880	-0.04 (VS)	0.665
	1 year	2 (4.2)	136.9 (48.4)	135.1 (108.8, 165.1)	25.8 (50.0)	0.53 (M)	1 (1.8)	136.7 (54.0)	135.3 (96.8, 165.1)	20.5 (62.8)	0.40 (S)	0.00 (VS)	0.959	-0.09 (VS)	0.611
	3 years	8 (16.7)	132.5 (36.0)	128.6 (114.7, 157.9)	23.8 (49.7)	0.49 (M)	7 (12.3)	131.0 (45.4)	129.9 (96.4, 161.2)	14.9 (59.9)	0.32 (S)	-0.04 (VS)	0.751	-0.16 (S)	0.453
Meat products (g/day)	Baseline	0 (0.0)	162.9 (43.0)	158.0 (134.5, 189.0)	Ref		0 (0.0)	158.8 (59.0)	143.4 (116.6, 198.4)	Ref		-0.08 (VS)	0.927		
	6 months	0 (0.0)	160.2 (37.7)	150.4 (134.1, 188.9)	-2.6 (56.4)	-0.06 (VS)	0 (0.0)	154.4 (41.8)	150.0 (122.8, 178.6)	-4.4 (66.7)	-0.09 (VS)	-0.15 (VS)	0.374	-0.03 (VS)	0.973
	1 year	2 (4.2)	165.4 (36.6)	162.9 (144.2, 188.5)	3.6 (56.2)	0.06 (VS)	1 (1.8)	158.4 (40.8)	159.0 (131.7, 183.2)	-2.4 (55.6)	-0.01 (VS)	-0.18 (S)	0.365	-0.11 (VS)	0.865
	3 years	8 (16.7)	152.5 (42.7)	151.4 (123.4, 184.6)	-7.6 (55.5)	-0.24 (S)	7 (12.3)	150.2 (33.1)	146.6 (134.7, 164.1)	-10.4 (63.9)	-0.18 (S)	-0.06 (VS)	0.527	-0.05 (VS)	0.703
Dairy products (g/day)	Baseline	0 (0.0)	354.2 (177.4)	328.6 (260.7, 407.0)	Ref		0 (0.0)	313.1 (152.1)	288.8 (210.0, 411.7)	Ref		-0.25 (S)	0.175		
	6 months	0 (0.0)	346.6 (164.2)	332.7 (282.5, 366.3)	-7.5 (212.9)	-0.04 (VS)	0 (0.0)	350.3 (158.1)	345.9 (275.0, 364.3)	37.3 (214.4)	0.24 (S)	0.02 (VS)	0.782	0.21 (S)	0.178
	1 year	2 (4.2)	374.1 (181.6)	332.1 (261.4, 378.2)	19.6 (224.3)	0.11 (VS)	1 (1.8)	359.3 (172.6)	345.3 (289.6, 390.9)	51.4 (191.9)	0.28 (S)	-0.08 (VS)	0.751	0.15 (S)	0.259
	3 years	8 (16.7)	371.5 (203.5)	345.9 (229.5, 430.2)	14.7 (238.1)	0.09 (VS)	7 (12.3)	337.3 (173.0)	305.7 (228.5, 418.9)	27.8 (209.3)	0.15 (VS)	-0.18 (S)	0.407	0.06 (VS)	0.812
MUFA (g/day)	Baseline	0 (0.0)	59.2 (15.1)	58.3 (51.9, 65.4)	Ref		0 (0.0)	56.0 (13.9)	58.1 (47.9, 62.1)	Ref		-0.22 (S)	0.271		
	6 months	0 (0.0)	63.7 (11.8)	65.8 (56.6, 70.6)	4.5 (20.0)	0.33 (S)	0 (0.0)	63.3 (14.9)	65.1 (54.9, 72.3)	7.4 (18.6)	0.51 (M)	-0.03 (VS)	0.823	0.15 (VS)	0.444
	1 year	2 (4.2)	64.6 (13.4)	65.8 (56.8, 73.7)	5.3 (20.7)	0.38 (S)	1 (1.8)	65.4 (14.6)	66.3 (54.9, 71.9)	9.5 (17.7)	0.66 (M)	0.06 (VS)	0.795	0.22 (S)	0.315
	3 years	8 (16.7)	56.4 (11.3)	57.2 (50.9, 61.5)	-2.7 (17.8)	-0.21 (S)	7 (12.3)	54.6 (13.8)	55.0 (43.3, 64.2)	-2.1 (19.2)	-0.10 (VS)	-0.14 (VS)	0.425	0.03 (VS)	0.667
n3-PUFA from fish (DHA+EPA) (g/day)	Baseline	0 (0.0)	0.8 (0.4)	0.7 (0.5, 1.2)	Ref		0 (0.0)	0.8 (0.4)	0.7 (0.5, 1.1)	Ref		0.02 (VS)	0.931		
	6 months	0 (0.0)	1.0 (0.4)	1.1 (0.6, 1.3)	0.2 (0.5)	0.59 (M)	0 (0.0)	0.9 (0.4)	1.1 (0.6, 1.2)	0.2 (0.5)	0.41 (S)	-0.13 (VS)	0.447	-0.11 (VS)	0.359
	1 year	2 (4.2)	1.0 (0.4)	1.1 (0.7, 1.3)	0.3 (0.5)	0.62 (M)	1 (1.8)	1.0 (0.4)	1.1 (0.6, 1.3)	0.2 (0.5)	0.43 (S)	-0.15 (VS)	0.370	-0.15 (VS)	0.337
	3 years	8 (16.7)	0.9 (0.4)	0.9 (0.5, 1.2)	0.2 (0.5)	0.36 (S)	7 (12.3)	1.0 (0.4)	1.1 (0.6, 1.2)	0.2 (0.5)	0.49 (M)	0.18 (S)	0.495	0.09 (VS)	0.917
Total n-3 PUFA (g/day)	Baseline	0 (0.0)	1.0 (0.5)	0.9 (0.6, 1.5)	Ref		0 (0.0)	1.0 (0.5)	0.9 (0.6, 1.5)	Ref		-0.02 (VS)	0.918		
	6 months	0 (0.0)	1.3 (0.5)	1.5 (0.8, 1.6)	0.3 (0.7)	0.56 (M)	0 (0.0)	1.2 (0.5)	1.5 (0.8, 1.6)	0.2 (0.7)	0.39 (S)	-0.18 (S)	0.315	-0.11 (VS)	0.330
	1 year	2 (4.2)	1.3 (0.5)	1.5 (0.9, 1.7)	0.3 (0.6)	0.57 (M)	1 (1.8)	1.2 (0.5)	1.5 (0.8, 1.6)	0.2 (0.7)	0.40 (S)	-0.18 (S)	0.286	-0.14 (VS)	0.326

	3 years	8 (16.7)	1.2 (0.5)	1.1 (0.7, 1.6)	0.2 (0.6)	0.25 (S)	7 (12.3)	1.3 (0.5)	1.5 (0.8, 1.6)	0.3 (0.7)	0.49 (M)	0.22 (S)	0.389	0.15 (VS)	0.740
	Baseline	0 (0.0)	19.2 (7.5)	17.1 (13.9, 23.5)	Ref		0 (0.0)	16.9 (5.3)	16.5 (13.0, 20.4)	Ref		-0.37 (S)	0.066		
Total PUFA	6 months	0 (0.0)	22.7 (4.1)	22.9 (19.5, 25.8)	3.5 (8.4)	0.57 (M)	0 (0.0)	21.9 (5.2)	22.7 (18.5, 25.9)	5.1 (7.0)	0.96 (L)	-0.16 (S)	0.505	0.21 (S)	0.360
(g/day)	1 year	2 (4.2)	22.5 (4.9)	22.5 (19.7, 25.8)	3.3 (9.1)	0.52 (M)	1 (1.8)	22.0 (5.5)	22.7 (17.7, 25.2)	5.3 (6.8)	0.95 (L)	-0.09 (VS)	0.634	0.25 (S)	0.299
	3 years	8 (16.7)	20.0 (5.7)	19.7 (16.5, 21.5)	1.1 (7.0)	0.11 (VS)	7 (12.3)	17.7 (4.1)	17.6 (15.2, 20.0)	0.5 (6.4)	0.18 (S)	-0.47 (M)	**0.027**	-0.09 (VS)	0.964
	Baseline	0 (0.0)	30.5 (10.2)	29.9 (23.8, 36.1)	Ref		0 (0.0)	27.4 (9.7)	26.2 (21.7, 30.9)	Ref		-0.31 (S)	0.133		
SAFA	6 months	0 (0.0)	23.2 (5.9)	21.8 (19.4, 25.2)	-7.3 (11.6)	-0.87 (L)	0 (0.0)	23.3 (5.9)	22.9 (19.4, 25.4)	-4.1 (7.4)	-0.51 (M)	0.02 (VS)	0.959	0.34 (S)	0.110
(g/day)	1 year	2 (4.2)	25.2 (6.0)	23.3 (21.0, 29.0)	-5.2 (10.9)	-0.63 (M)	1 (1.8)	25.0 (8.5)	22.5 (19.9, 27.6)	-2.4 (9.2)	-0.26 (S)	-0.02 (VS)	0.962	0.29 (S)	0.284
	3 years	8 (16.7)	24.9 (7.7)	24.0 (20.8, 30.0)	-5.4 (11.9)	-0.62 (M)	7 (12.3)	23.0 (6.2)	22.7 (18.3, 26.6)	-4.4 (10.0)	-0.53 (M)	-0.27 (S)	0.154	0.09 (VS)	0.770
	Baseline	0 (0.0)	116.2 (32.1)	110.7 (96.4, 135.3)	Ref		0 (0.0)	106.9 (26.1)	106.5 (93.4, 123.4)	Ref		-0.32 (S)	0.116		
Total fat	6 months	0 (0.0)	112.3 (16.2)	112.2 (103.1, 123.1)	-3.9 (37.0)	-0.15 (S)	0 (0.0)	110.9 (21.2)	111.4 (101.8, 123.4)	4.0 (26.8)	0.17 (S)	-0.07 (VS)	0.668	0.25 (S)	0.242
(g/day)	1 year	2 (4.2)	117.4 (20.4)	118.4 (107.2, 129.3)	1.2 (37.6)	0.04 (VS)	1 (1.8)	115.9 (24.7)	112.5 (100.5, 126.6)	9.2 (28.7)	0.35 (S)	-0.07 (VS)	0.772	0.24 (S)	0.394
	3 years	8 (16.7)	107.7 (21.3)	105.9 (97.7, 122.6)	-7.6 (34.1)	-0.31 (S)	7 (12.3)	101.0 (22.1)	104.0 (84.9, 115.7)	-6.9 (32.0)	-0.24 (S)	-0.31 (S)	0.117	0.02 (VS)	0.813
	Baseline	0 (0.0)	0.7 (0.5)	0.6 (0.4, 0.9)	Ref		0 (0.0)	0.6 (0.4)	0.5 (0.3, 0.7)	Ref		-0.35 (S)	0.098		
Trans fatty	6 months	0 (0.0)	0.3 (0.2)	0.3 (0.2, 0.4)	-0.4 (0.5)	-1.09 (L)	0 (0.0)	0.3 (0.2)	0.3 (0.2, 0.4)	-0.3 (0.4)	-0.78 (L)	-0.02 (VS)	0.738	0.34 (S)	0.074
acids (g/day)	1 year	2 (4.2)	0.4 (0.2)	0.3 (0.2, 0.6)	-0.3 (0.5)	-0.87 (L)	1 (1.8)	0.4 (0.3)	0.3 (0.2, 0.5)	-0.2 (0.4)	-0.65 (M)	-0.19 (S)	0.426	0.22 (S)	0.627
	3 years	8 (16.7)	0.4 (0.3)	0.4 (0.2, 0.5)	-0.3 (0.6)	-0.71 (M)	7 (12.3)	0.4 (0.2)	0.3 (0.2, 0.5)	-0.2 (0.4)	-0.61 (M)	-0.21 (S)	0.292	0.17 (S)	0.385

Positive/negative values for 6 months, 1 year and 3 years change indicate increase and decrease, respectively, compared to the baseline value.

P-values of cross-sectional differences were obtained from the analysis of variance (ANOVA) from multivariable-adjusted linear models. P-values of differences in the rate of change were obtained from linear mixed effects models. All the analyses were adjusted by intervention group, age, and diagnostic of type 2 diabetes. Dependent variables were normalized before estimating p-values, using ordered quantile normalization (ORQ) transformation.

N=number. 95%CI= 95% confidence intervals

[1]Effect Size: VS = very small (Cohen's d < 0.2); S = small [Cohen's d (0.2–0.5)]; M = medium [Cohen's d (0.5–0.8)]; L = large [Cohen's d (0.8–1.2)]; VL = very large (Cohen's d ≥ 1.2).

Bold values denote statistical significance at P<0.05.

Supplementary Table 3. Change in fatty acids from baseline to 6 months, 1 year and 3 years by sex

		Men [N=48]					Women [N=57]					Sex differences			
			Values at each time point		Change from baseline			Values at each time point		Change from baseline		At each time point		In change over time	
Compound (μM)	Time	Missing [N (%)]	Mean (SD)	Median (Q1, Q3)	Mean change (SD)	Cohen's d (size)[1]	Missing [N (%)]	Mean (SD)	Median (Q1, Q3)	Mean change (SD)	Cohen's d (size)[1]	Cohen's d (size)[1]	P-value*	Cohen's d (size)[1]	P-value*
C12:0	Baseline	0 (0.0)	4.0 (11.4)	2.0 (1.5, 2.4)	Ref		0 (0.0)	1.9 (0.8)	1.7 (1.5, 2.0)	Ref		-0.26 (S)	0.261		
	6 months	0 (0.0)	1.9 (0.7)	1.8 (1.5, 2.1)	-2.0 (11.0)	-0.25 (S)	0 (0.0)	1.9 (0.5)	1.7 (1.5, 2.1)	-0.1 (0.8)	-0.10 (VS)	-0.11 (VS)	0.623	0.26 (S)	0.086
	1 year	5 (10.4)	2.1 (1.0)	1.8 (1.5, 2.1)	-0.1 (1.2)	-0.22 (S)	1 (1.8)	1.9 (0.7)	1.6 (1.5, 2.0)	-0.1 (0.9)	-0.08 (VS)	-0.30 (S)	0.245	0.08 (VS)	0.689
	3 years	8 (16.7)	2.1 (1.3)	1.7 (1.5, 2.1)	-0.2 (1.6)	-0.22 (S)	7 (12.3)	2.0 (0.7)	1.9 (1.5, 2.2)	0.1 (1.0)	0.05 (VS)	-0.10 (VS)	0.625	0.25 (S)	0.052
C14:0	Baseline	0 (0.0)	29.4 (26.2)	22.1 (17.9, 34.6)	Ref		0 (0.0)	22.4 (9.3)	19.6 (14.7, 27.1)	Ref		-0.37 (S)	0.093		
	6 months	0 (0.0)	23.6 (10.2)	22.1 (15.9, 28.0)	-5.8 (22.5)	-0.29 (S)	0 (0.0)	21.8 (10.1)	18.5 (14.7, 25.7)	-0.6 (7.1)	-0.06 (VS)	-0.18 (S)	0.332	0.32 (S)	0.208
	1 year	5 (10.4)	26.2 (16.6)	20.1 (15.9, 26.8)	0.6 (11.4)	-0.14 (VS)	1 (1.8)	23.0 (15.4)	18.2 (15.0, 22.4)	0.6 (14.0)	0.05 (VS)	-0.20 (S)	0.471	-0.01 (VS)	0.934
	3 years	8 (16.7)	21.5 (10.2)	19.3 (15.5, 24.9)	-4.4 (11.0)	-0.38 (S)	7 (12.3)	23.4 (14.0)	18.9 (15.1, 27.5)	1.6 (14.8)	0.09 (VS)	0.15 (S)	0.373	0.45 (S)	0.054
C15:0	Baseline	0 (0.0)	4.6 (2.6)	3.8 (3.2, 5.0)	Ref		0 (0.0)	4.3 (1.8)	4.0 (2.8, 5.7)	Ref		-0.14 (VS)	0.556		
	6 months	0 (0.0)	5.0 (6.0)	3.6 (2.8, 4.5)	0.3 (5.6)	0.07 (VS)	0 (0.0)	4.3 (2.1)	3.7 (3.0, 4.7)	0.0 (1.4)	0.02 (VS)	-0.14 (VS)	0.356	-0.08 (VS)	0.093
	1 year	5 (10.4)	4.7 (3.1)	3.7 (2.9, 4.7)	0.3 (2.3)	0.02 (VS)	1 (1.8)	4.6 (3.6)	3.7 (2.9, 4.1)	0.3 (3.5)	0.11 (VS)	-0.01 (VS)	0.819	0.00 (VS)	0.654
	3 years	8 (16.7)	3.8 (1.8)	3.4 (2.7, 4.2)	-0.6 (1.8)	-0.37 (S)	7 (12.3)	4.8 (3.5)	3.9 (3.2, 4.9)	0.6 (3.6)	0.17 (S)	0.35 (S)	0.068	0.42 (S)	**0.036**
C16:0	Baseline	0 (0.0)	1106.4 (592.2)	993.4 (797.0, 1138.5)	Ref		0 (0.0)	895.1 (266.0)	814.4 (699.8, 1057.0)	Ref		-0.47 (M)	**0.028**		
	6 months	0 (0.0)	947.9 (331.6)	849.3 (695.3, 1121.9)	-158.4 (449.5)	-0.33 (S)	0 (0.0)	899.0 (329.7)	823.0 (696.5, 971.7)	3.9 (247.4)	0.01 (VS)	-0.15 (VS)	0.453	0.46 (M)	**0.024**
	1 year	5 (10.4)	1102.2 (660.9)	903.0 (697.3, 1068.2)	63.6 (515.1)	-0.01 (VS)	1 (1.8)	940.1 (557.3)	788.9 (675.9, 926.7)	42.0 (527.1)	0.10 (VS)	-0.27 (S)	0.300	-0.04 (VS)	0.847
	3 years	8 (16.7)	918.5 (445.9)	812.3 (647.3, 1031.4)	-121.4 (461.6)	-0.35 (S)	7 (12.3)	956.5 (603.3)	778.7 (653.1, 933.6)	77.1 (630.9)	0.13 (VS)	0.07 (VS)	0.590	0.35 (S)	0.166
C16:1n7	Baseline	0 (0.0)	21.8 (24.6)	13.2 (9.7, 22.7)	Ref		0 (0.0)	15.7 (7.9)	14.2 (9.4, 20.2)	Ref		-0.35 (S)	0.106		
	6 months	0 (0.0)	13.7 (10.8)	10.1 (5.9, 18.3)	-8.0 (18.9)	-0.42 (S)	0 (0.0)	13.7 (6.9)	11.8 (9.3, 16.9)	-2.0 (6.1)	-0.26 (S)	0.00 (VS)	0.951	0.45 (S)	**0.020**
	1 year	5 (10.4)	17.0 (20.2)	9.3 (5.6, 15.1)	-1.1 (14.8)	-0.21 (S)	1 (1.8)	14.8 (11.6)	11.0 (8.2, 16.4)	-0.9 (11.0)	-0.09 (VS)	-0.14 (VS)	0.604	0.02 (VS)	0.299
	3 years	8 (16.7)	11.1 (10.9)	8.4 (5.4, 11.5)	-7.3 (17.2)	-0.55 (M)	7 (12.3)	16.2 (12.5)	12.1 (7.6, 21.0)	0.6 (13.2)	0.05 (VS)	0.43 (S)	**0.029**	0.53 (M)	**0.002**
C17:0	Baseline	0 (0.0)	10.9 (4.3)	9.8 (8.5, 11.9)	Ref		0 (0.0)	9.9 (2.7)	9.3 (7.8, 12.0)	Ref		-0.29 (S)	0.169		
	6 months	0 (0.0)	10.4 (3.7)	9.3 (7.9, 11.6)	-0.5 (3.9)	-0.12 (VS)	0 (0.0)	10.3 (3.3)	9.5 (7.9, 11.6)	0.4 (2.6)	0.15 (S)	-0.02 (VS)	0.770	0.28 (S)	**0.046**
	1 year	5 (10.4)	11.5 (5.8)	10.0 (8.5, 11.6)	1.0 (4.8)	0.14 (VS)	1 (1.8)	10.6 (5.6)	9.2 (7.8, 10.9)	0.7 (5.5)	0.16 (S)	-0.17 (S)	0.549	-0.06 (VS)	0.991
	3 years	8 (16.7)	10.2 (3.7)	8.8 (8.2, 11.0)	-0.4 (3.4)	-0.16 (S)	7 (12.3)	10.7 (5.8)	9.1 (7.7, 11.4)	1.1 (6.0)	0.19 (S)	0.10 (VS)	0.525	0.30 (S)	0.254
C18:0	Baseline	0 (0.0)	754.6 (291.6)	682.1 (598.9, 791.5)	Ref		0 (0.0)	661.7 (169.0)	625.5 (547.9, 746.6)	Ref		-0.40 (S)	0.064		
	6 months	0 (0.0)	683.7 (194.9)	620.9 (533.8, 769.7)	-70.9 (222.3)	-0.29 (S)	0 (0.0)	682.6 (232.3)	645.3 (542.7, 721.2)	20.9 (190.4)	0.10 (VS)	-0.01 (VS)	0.983	0.45 (S)	**0.035**
	1 year	5 (10.4)	801.9 (419.4)	687.8 (575.9, 780.6)	66.8 (351.3)	0.13 (VS)	1 (1.8)	707.9 (347.7)	628.3 (531.9, 694.2)	43.8 (347.9)	0.17 (S)	-0.25 (S)	0.351	-0.07 (VS)	0.991
	3 years	8 (16.7)	701.5 (291.5)	613.1 (527.1, 751.1)	-34.9 (281.7)	-0.18 (S)	7 (12.3)	708.4 (385.6)	587.3 (520.3, 705.3)	59.2 (416.7)	0.16 (S)	0.02 (VS)	0.766	0.26 (S)	0.349
C18:1n9	Baseline	0 (0.0)	766.4 (413.2)	674.1 (529.1, 794.4)	Ref		0 (0.0)	626.3 (200.9)	562.7 (489.0, 734.9)	Ref		-0.44 (S)	**0.039**		
	6 months	0 (0.0)	659.1 (217.4)	604.5 (481.4, 820.7)	-107.3 (319.8)	-0.33 (S)	0 (0.0)	632.2 (228.1)	590.8 (485.8, 726.9)	5.9 (165.2)	0.03 (VS)	-0.12 (VS)	0.551	0.46 (M)	**0.028**
	1 year	5 (10.4)	745.9 (403.9)	625.9 (506.6, 753.7)	26.4 (370.2)	-0.05 (VS)	1 (1.8)	651.3 (346.4)	553.9 (469.9, 685.5)	23.2 (309.5)	0.09 (VS)	-0.25 (S)	0.326	-0.01 (VS)	0.705
	3 years	8 (16.7)	657.1 (295.7)	616.3 (467.9, 718.1)	-61.6 (368.0)	-0.30 (S)	7 (12.3)	684.2 (406.2)	561.2 (463.9, 704.8)	68.7 (405.9)	0.18 (S)	0.07 (VS)	0.565	0.33 (S)	0.225
C18:2n6	Baseline	0 (0.0)	508.1 (389.2)	417.7 (359.8, 526.5)	Ref		0 (0.0)	419.8 (131.1)	383.9 (323.9, 489.3)	Ref		-0.32 (S)	0.151		
	6 months	0 (0.0)	426.4 (130.8)	393.7 (324.2, 495.6)	-81.7 (320.3)	-0.28 (S)	0 (0.0)	426.8 (160.1)	400.6 (326.5, 495.8)	7.0 (122.0)	0.05 (VS)	0.00 (VS)	0.996	0.38 (S)	0.094
	1 year	5 (10.4)	495.6 (265.2)	417.9 (350.0, 533.2)	41.6 (215.1)	-0.04 (VS)	1 (1.8)	432.2 (227.0)	373.0 (314.6, 464.0)	11.8 (222.5)	0.07 (VS)	-0.26 (S)	0.294	-0.14 (VS)	0.442
	3 years	8 (16.7)	425.1 (177.9)	380.8 (329.0, 459.9)	-30.2 (190.9)	-0.27 (S)	7 (12.3)	445.2 (263.2)	382.4 (298.4, 480.2)	29.8 (266.1)	0.12 (VS)	0.09 (VS)	0.576	0.25 (S)	0.481
C18:3n3	Baseline	0 (0.0)	3.0 (2.6)	2.1 (1.6, 3.5)	Ref		0 (0.0)	2.9 (1.8)	2.4 (1.5, 3.6)	Ref		-0.08 (VS)	0.676		
	6 months	0 (0.0)	3.3 (2.2)	2.9 (1.8, 4.1)	0.3 (2.1)	0.13 (VS)	0 (0.0)	3.0 (1.6)	2.7 (1.6, 4.0)	0.2 (1.8)	0.11 (VS)	-0.16 (S)	0.400	-0.07 (VS)	0.453
	1 year	5 (10.4)	3.3 (3.4)	2.7 (1.9, 3.8)	0.6 (3.2)	0.10 (VS)	1 (1.8)	3.3 (3.0)	2.2 (1.7, 3.8)	0.5 (2.9)	0.18 (S)	-0.01 (VS)	0.991	-0.04 (VS)	0.501
	3 years	8 (16.7)	2.7 (1.5)	2.5 (1.6, 3.4)	-0.1 (2.5)	-0.16 (S)	7 (12.3)	3.3 (2.6)	2.6 (1.4, 3.6)	0.4 (2.8)	0.20 (S)	0.29 (S)	0.167	0.21 (S)	0.893
C20:0	Baseline	0 (0.0)	3.7 (1.6)	3.1 (2.7, 4.3)	Ref		0 (0.0)	3.1 (1.0)	2.9 (2.4, 3.6)	Ref		-0.41 (S)	0.064		
	6 months	0 (0.0)	3.4 (1.2)	3.1 (2.5, 4.4)	-0.3 (1.3)	-0.19 (S)	0 (0.0)	3.3 (1.1)	3.1 (2.5, 3.6)	0.1 (1.1)	0.12 (VS)	-0.12 (VS)	0.558	0.33 (S)	0.085
	1 year	5 (10.4)	4.0 (2.6)	3.1 (2.8, 3.7)	0.5 (2.2)	0.15 (VS)	1 (1.8)	3.3 (1.6)	3.0 (2.5, 3.5)	0.2 (1.7)	0.14 (VS)	-0.31 (S)	0.231	-0.15 (VS)	0.884
	3 years	8 (16.7)	3.4 (1.9)	2.8 (2.4, 3.7)	-0.1 (1.7)	-0.18 (S)	7 (12.3)	3.5 (2.1)	3.0 (2.5, 3.5)	0.4 (2.3)	0.20 (S)	0.05 (VS)	0.631	0.25 (S)	0.206
C20:1n9	Baseline	0 (0.0)	8.7 (3.6)	7.8 (6.2, 9.8)	Ref		0 (0.0)	7.2 (2.4)	6.9 (5.5, 8.3)	Ref		-0.50 (M)	**0.019**		
	6 months	0 (0.0)	8.3 (2.9)	7.8 (6.1, 9.8)	-0.4 (2.9)	-0.12 (VS)	0 (0.0)	7.6 (2.9)	6.8 (5.6, 8.7)	0.4 (2.6)	0.14 (VS)	-0.25 (S)	0.225	0.28 (S)	0.160
	1 year	5 (10.4)	9.7 (6.6)	7.8 (6.6, 9.5)	1.2 (5.5)	0.18 (S)	1 (1.8)	7.8 (4.0)	6.7 (5.8, 7.9)	0.6 (4.1)	0.17 (S)	-0.35 (S)	0.143	-0.13 (VS)	0.954
	3 years	8 (16.7)	8.4 (4.2)	7.1 (5.8, 9.4)	0.0 (4.0)	-0.08 (VS)	7 (12.3)	8.3 (5.8)	6.7 (5.5, 8.0)	1.2 (6.0)	0.25 (S)	-0.02 (VS)	0.940	0.24 (S)	0.362
C20:2n6	Baseline	0 (0.0)	8.5 (4.9)	7.0 (5.7, 9.1)	Ref		0 (0.0)	6.5 (2.2)	5.9 (5.1, 7.8)	Ref		-0.54 (M)	**0.013**		
	6 months	0 (0.0)	7.3 (2.5)	7.0 (5.3, 8.9)	-1.2 (3.7)	-0.31 (S)	0 (0.0)	6.7 (2.6)	6.2 (5.0, 7.4)	0.2 (2.2)	0.08 (VS)	-0.23 (S)	0.274	0.47 (M)	0.062
	1 year	5 (10.4)	8.9 (5.5)	7.0 (5.9, 9.4)	0.9 (4.6)	0.08 (VS)	1 (1.8)	7.2 (4.2)	6.0 (4.9, 7.9)	0.7 (4.2)	0.22 (S)	-0.35 (S)	0.157	-0.04 (VS)	0.863
	3 years	8 (16.7)	7.7 (4.4)	6.2 (5.5, 8.3)	-0.3 (4.5)	-0.16 (S)	7 (12.3)	7.4 (5.2)	5.7 (4.6, 7.3)	1.0 (5.4)	0.23 (S)	-0.07 (VS)	0.915	0.25 (S)	0.700
C20:3n6	Baseline	0 (0.0)	58.7 (25.9)	50.0 (43.2, 64.0)	Ref		0 (0.0)	53.3 (16.8)	51.6 (41.8, 60.6)	Ref		-0.25 (S)	0.221		
	6 months	0 (0.0)	49.7 (20.2)	44.0 (38.9, 61.9)	-9.0 (21.0)	-0.39 (S)	0 (0.0)	53.5 (18.7)	48.4 (41.8, 59.8)	0.2 (16.9)	0.01 (VS)	0.20 (S)	0.310	0.49 (M)	0.005
	1 year	5 (10.4)	59.9 (34.8)	49.4 (40.4, 63.6)	3.2 (30.8)	0.04 (VS)	1 (1.8)	60.2 (41.7)	49.7 (39.0, 63.5)	7.1 (42.0)	0.22 (S)	0.01 (VS)	0.760	0.10 (VS)	0.522

	3 years	8 (16.7)	51.5 (21.1)	46.0 (37.5, 58.8)	-5.0 (26.1)	-0.30 (S)	7 (12.3)	59.5 (41.8)	48.3 (39.3, 58.7)	7.0 (44.0)	0.20 (S)	0.23 (S)	0.188	0.32 (S)	0.246
C20:4n6	Baseline	0 (0.0)	609.9 (260.5)	546.2 (452.0, 642.4)	Ref		0 (0.0)	552.7 (157.0)	524.7 (440.7, 641.5)	Ref		-0.27 (S)	0.237		
	6 months	0 (0.0)	560.8 (159.5)	526.9 (439.6, 661.3)	-49.2 (191.7)	-0.23 (S)	0 (0.0)	564.4 (174.1)	526.8 (466.8, 589.2)	11.7 (174.0)	0.07 (VS)	0.02 (VS)	0.809	0.33 (S)	0.196
	1 year	5 (10.4)	649.4 (326.1)	595.0 (466.2, 658.1)	52.9 (282.7)	0.13 (VS)	1 (1.8)	598.0 (251.2)	540.0 (467.9, 622.3)	43.1 (272.9)	0.22 (S)	-0.18 (S)	0.566	-0.04 (VS)	0.858
	3 years	8 (16.7)	596.3 (244.0)	498.1 (460.2, 651.6)	0.9 (258.6)	-0.05 (VS)	7 (12.3)	597.9 (276.3)	510.3 (461.6, 640.1)	53.1 (327.4)	0.20 (S)	0.01 (VS)	0.757	0.17 (S)	0.709
C20:5n3	Baseline	0 (0.0)	16.4 (11.2)	14.2 (9.3, 19.4)	Ref		0 (0.0)	18.3 (10.7)	17.1 (11.7, 22.0)	Ref		0.17 (S)	0.529		
	6 months	0 (0.0)	18.7 (11.1)	15.5 (11.9, 25.2)	2.3 (7.8)	0.20 (S)	0 (0.0)	19.5 (9.5)	16.7 (12.6, 25.5)	1.2 (10.9)	0.12 (VS)	0.08 (VS)	0.899	-0.11 (VS)	0.420
	1 year	5 (10.4)	21.5 (14.4)	21.3 (12.4, 24.6)	5.6 (14.9)	0.40 (S)	1 (1.8)	20.7 (13.6)	18.3 (12.3, 23.4)	2.4 (15.1)	0.20 (S)	-0.06 (VS)	0.811	-0.21 (S)	0.138
	3 years	8 (16.7)	16.1 (7.5)	14.2 (10.5, 21.0)	0.8 (8.8)	-0.03 (VS)	7 (12.3)	20.4 (17.8)	14.6 (10.7, 20.7)	2.8 (18.8)	0.15 (VS)	0.30 (S)	0.239	0.14 (VS)	0.771
C22:0	Baseline	0 (0.0)	3.1 (2.0)	2.6 (2.1, 3.6)	Ref		0 (0.0)	2.7 (1.1)	2.3 (1.9, 3.1)	Ref		-0.29 (S)	0.145		
	6 months	0 (0.0)	2.8 (1.2)	2.4 (1.9, 3.2)	-0.3 (1.7)	-0.20 (S)	0 (0.0)	2.7 (1.2)	2.3 (1.9, 3.0)	0.0 (1.0)	0.01 (VS)	-0.10 (VS)	0.520	0.25 (S)	0.303
	1 year	5 (10.4)	3.2 (2.0)	2.7 (2.1, 3.7)	0.4 (1.7)	0.06 (VS)	1 (1.8)	2.9 (1.9)	2.3 (1.9, 3.1)	0.2 (1.8)	0.15 (VS)	-0.18 (S)	0.505	-0.08 (VS)	0.964
	3 years	8 (16.7)	2.7 (1.4)	2.2 (1.7, 3.0)	-0.2 (1.3)	-0.25 (S)	7 (12.3)	2.8 (1.8)	2.2 (1.9, 3.1)	0.2 (1.9)	0.12 (VS)	0.09 (VS)	0.581	0.22 (S)	0.286
C22:1n9	Baseline	0 (0.0)	9.6 (7.4)	6.8 (4.8, 12.2)	Ref		0 (0.0)	13.3 (33.2)	7.2 (5.2, 12.6)	Ref		0.15 (VS)	0.417		
	6 months	0 (0.0)	15.4 (33.0)	7.2 (5.0, 13.7)	5.8 (32.2)	0.24 (S)	0 (0.0)	9.5 (7.0)	7.1 (5.5, 11.4)	-3.9 (28.4)	-0.16 (S)	-0.26 (S)	0.172	-0.32 (S)	0.370
	1 year	5 (10.4)	11.9 (12.1)	8.5 (4.6, 12.6)	2.0 (12.4)	0.24 (S)	1 (1.8)	9.8 (7.0)	7.6 (5.4, 10.8)	-3.7 (34.2)	-0.15 (VS)	-0.22 (S)	0.373	-0.21 (S)	0.481
	3 years	8 (16.7)	10.4 (8.4)	7.1 (5.9, 15.1)	0.6 (9.9)	0.10 (VS)	7 (12.3)	8.9 (6.7)	7.3 (4.9, 10.0)	-4.7 (36.3)	-0.18 (S)	-0.20 (S)	0.441	-0.19 (S)	0.209
C22:6n3	Baseline	0 (0.0)	173.8 (79.1)	158.5 (132.9, 194.7)	Ref		0 (0.0)	173.2 (66.3)	157.8 (134.0, 206.7)	Ref		-0.01 (VS)	0.915		
	6 months	0 (0.0)	176.0 (65.9)	167.5 (133.1, 192.8)	2.2 (58.3)	0.03 (VS)	0 (0.0)	182.0 (75.3)	165.3 (141.4, 194.9)	8.8 (71.2)	0.12 (VS)	0.08 (VS)	0.816	0.10 (VS)	0.835
	1 year	5 (10.4)	200.0 (103.2)	174.1 (142.3, 220.1)	32.8 (96.1)	0.29 (S)	1 (1.8)	190.3 (93.7)	160.8 (147.3, 199.6)	16.5 (106.4)	0.21 (S)	-0.10 (VS)	0.736	-0.16 (S)	0.633
	3 years	8 (16.7)	178.5 (72.8)	159.3 (133.0, 204.7)	13.4 (73.0)	0.06 (VS)	7 (12.3)	193.3 (116.7)	160.9 (132.9, 203.5)	26.4 (129.0)	0.22 (S)	0.15 (VS)	0.468	0.12 (VS)	0.946
C24:0	Baseline	0 (0.0)	6.4 (2.8)	5.6 (4.8, 7.5)	Ref		0 (0.0)	5.2 (1.9)	5.0 (4.1, 6.1)	Ref		-0.52 (M)	**0.012**		
	6 months	0 (0.0)	5.7 (2.2)	5.0 (4.3, 7.0)	-0.7 (2.2)	-0.29 (S)	0 (0.0)	5.3 (2.2)	4.9 (3.9, 6.1)	0.1 (1.8)	0.04 (VS)	-0.18 (S)	0.351	0.41 (S)	0.057
	1 year	5 (10.4)	6.6 (3.6)	5.9 (4.7, 6.7)	0.3 (3.1)	0.04 (VS)	1 (1.8)	5.5 (3.1)	4.5 (3.8, 6.2)	0.2 (3.0)	0.11 (VS)	-0.32 (S)	0.206	-0.02 (VS)	0.874
	3 years	8 (16.7)	5.6 (2.6)	4.9 (3.9, 6.3)	-0.6 (2.4)	-0.33 (S)	7 (12.3)	5.5 (2.9)	4.5 (3.6, 5.9)	0.3 (3.3)	0.09 (VS)	-0.04 (VS)	0.986	0.31 (S)	0.229

Positive/negative values for 6 months, 1 year and 3 years change indicate increase and decrease, respectively, compared to the baseline value.
P-values of cross-sectional differences were obtained from the analysis of variance (ANOVA) from multivariable-adjusted linear models. P-values of differences in the rate of change were obtained from linear mixed effects models. All the analyses were adjusted by intervention group, age, and diagnostic of type 2 diabetes. Dependent variables were normalized before estimating p-values, using ordered quantile normalization (ORQ) transformation.
N=number. 95%CI= 95% confidence intervals
[1]Effect Size: VS = very small (Cohen's d < 0.2); S = small [Cohen's d (0.2–0.5)]; M = medium [Cohen's d (0.5–0.8)]; L = large [Cohen's d (0.8–1.2)]; VL = very large (Cohen's d ≥ 1.2).
Bold values denote statistical significance at P<0.05.

Supplementary Table 4. Change in eCBs concentrations from baseline to 6 months, 1 year and 3 years in the overall population

		Values at each time point		Change from baseline			
**Compound**	**Time**	**Mean (SD)**	**Median (Q1, Q3)**	**Absolute mean change (SD)**	**% mean change (SD)**	**Cohen's d (effect size)[1]**	**P-value***
AEA (nM)	Baseline	0.77 (0.24)	0.75 (0.61, 0.92)	Ref	Ref		
	6 months	0.72 (0.26)	0.68 (0.56, 0.84)	-0.05 (0.21)	-4.9 (25.2)	-0.21 (S)	**0.003**
	1 year	0.76 (0.23)	0.73 (0.60, 0.91)	-0.02 (0.23)	1.8 (31.4)	-0.06 (VS)	0.463
	3 years	0.83 (0.25)	0.78 (0.65, 0.97)	0.06 (0.27)	13.1 (38.9)	0.25 (S)	**0.019**
2-AG (nM)	Baseline	4.78 (3.23)	3.82 (3.06, 5.47)	Ref	Ref		
	6 months	3.94 (2.49)	3.25 (2.49, 4.50)	-0.84 (2.47)	-10.6 (41.5)	-0.29 (S)	**<0.001**
	1 year	3.89 (2.90)	3.35 (2.51, 4.16)	-0.65 (3.14)	-5.7 (63.4)	-0.29 (S)	**<0.001**
	3 years	3.92 (1.66)	3.61 (2.59, 4.86)	-0.69 (2.57)	-3.9 (47.3)	-0.33 (S)	**0.004**
DEA (nM)	Baseline	0.18 (0.05)	0.18 (0.15, 0.21)	Ref	Ref		
	6 months	0.17 (0.05)	0.16 (0.14, 0.19)	-0.02 (0.04)	-7.8 (20.7)	-0.33 (S)	**<0.001**
	1 year	0.18 (0.05)	0.17 (0.14, 0.20)	-0.01 (0.04)	-1.1 (23.5)	-0.13 (VS)	0.102
	3 years	0.19 (0.05)	0.18 (0.16, 0.20)	0.00 (0.05)	3.5 (23.8)	0.08 (VS)	0.676
DGLEA (nM)	Baseline	0.16 (0.06)	0.16 (0.13, 0.19)	Ref	Ref		
	6 months	0.15 (0.05)	0.14 (0.11, 0.18)	-0.01 (0.04)	-3.8 (27.2)	-0.19 (S)	**0.005**
	1 year	0.16 (0.05)	0.15 (0.13, 0.19)	0.00 (0.04)	2.7 (26.4)	-0.04 (VS)	0.681
	3 years	0.17 (0.05)	0.16 (0.14, 0.20)	0.00 (0.04)	7.2 (30.6)	0.09 (VS)	0.261
DHEA (nM)	Baseline	1.23 (0.43)	1.22 (0.93, 1.46)	Ref	Ref		
	6 months	1.18 (0.38)	1.16 (0.91, 1.41)	-0.06 (0.33)	-1.3 (24.0)	-0.14 (VS)	0.068
	1 year	1.25 (0.44)	1.20 (0.94, 1.45)	0.02 (0.35)	5.5 (27.7)	0.04 (VS)	0.538
	3 years	1.26 (0.39)	1.21 (0.99, 1.48)	0.03 (0.33)	6.7 (29.2)	0.06 (VS)	0.397
LEA (nM)	Baseline	2.19 (0.67)	2.11 (1.70, 2.61)	Ref	Ref		
	6 months	2.03 (0.62)	1.94 (1.58, 2.37)	-0.15 (0.59)	-3.4 (26.2)	-0.23 (S)	**0.009**
	1 year	2.16 (0.54)	2.11 (1.79, 2.49)	0.00 (0.59)	4.3 (27.0)	-0.05 (VS)	0.899
	3 years	2.28 (0.65)	2.17 (1.84, 2.70)	0.10 (0.66)	8.9 (29.9)	0.14 (VS)	0.102
OEA (nM)	Baseline	10.32 (2.48)	10.14 (8.69, 12.25)	Ref	Ref		
	6 months	9.99 (2.52)	9.87 (8.28, 11.23)	-0.36 (2.17)	-1.1 (21.0)	-0.13 (VS)	0.103
	1 year	10.66 (2.39)	10.70 (8.91, 11.69)	0.27 (2.39)	5.9 (25.1)	0.14 (VS)	0.133
	3 years	11.22 (2.75)	10.75 (9.31, 12.83)	0.88 (2.68)	12.4 (30.7)	0.35 (S)	**0.002**
PEA (nM)	Baseline	18.39 (3.87)	18.32 (15.70, 21.19)	Ref	Ref		
	6 months	17.13 (3.81)	16.66 (14.60, 19.24)	-1.28 (3.33)	-5.4 (18.0)	-0.33 (S)	**<0.001**
	1 year	18.21 (3.87)	17.88 (15.85, 20.37)	-0.07 (3.47)	1.4 (19.6)	-0.05 (VS)	0.769
	3 years	18.52 (3.76)	18.13 (15.42, 21.00)	0.23 (3.76)	3.6 (21.2)	0.03 (VS)	0.550
POEA (nM)	Baseline	1.86 (0.87)	1.74 (1.15, 2.53)	Ref	Ref		
	6 months	1.69 (0.77)	1.55 (1.10, 2.05)	-0.19 (0.64)	-1.4 (40.2)	-0.21 (S)	**0.017**
	1 year	1.82 (0.76)	1.73 (1.26, 2.17)	-0.05 (0.70)	7.4 (44.5)	-0.05 (VS)	0.952
	3 years	1.88 (1.02)	1.65 (1.23, 2.23)	0.02 (0.83)	9.0 (45.0)	0.02 (VS)	0.966
SEA (nM)	Baseline	4.73 (1.04)	4.58 (4.00, 5.42)	Ref	Ref		
	6 months	4.34 (1.00)	4.29 (3.65, 4.86)	-0.39 (0.82)	-7.0 (16.8)	-0.39 (S)	**<0.001**
	1 year	4.71 (1.14)	4.57 (3.90, 5.35)	0.00 (0.91)	1.3 (18.5)	-0.02 (VS)	0.678
	3 years	4.79 (1.07)	4.63 (4.03, 5.47)	0.04 (0.93)	2.4 (18.4)	0.05 (VS)	0.758
DHEA/AEA	Baseline	1.67 (0.55)	1.61 (1.27, 1.96)	Ref	Ref		
	6 months	1.72 (0.58)	1.67 (1.31, 1.95)	0.06 (0.42)	6.5 (24.1)	0.10 (VS)	0.054
	1 year	1.73 (0.61)	1.64 (1.35, 2.00)	0.09 (0.51)	9.3 (33.0)	0.12 (VS)	0.200
	3 years	1.59 (0.50)	1.62 (1.26, 1.86)	-0.07 (0.43)	-0.8 (26.7)	-0.15 (VS)	0.128
OEA/AEA	Baseline	13.74 (2.29)	13.37 (12.25, 15.28)	Ref	Ref		
	6 months	14.45 (2.65)	13.90 (12.42, 16.08)	0.72 (1.76)	5.8 (12.6)	0.29 (S)	**<0.001**
	1 year	14.65 (2.82)	14.44 (12.32, 16.20)	0.87 (2.11)	7.0 (15.8)	0.35 (S)	**<0.001**
	3 years	13.95 (2.74)	13.62 (12.19, 16.01)	0.23 (2.09)	2.2 (15.2)	0.08 (VS)	0.568
OEA/PEA	Baseline	0.56 (0.07)	0.56 (0.51, 0.61)	Ref	Ref		
	6 months	0.58 (0.07)	0.59 (0.53, 0.63)	0.02 (0.05)	4.5 (9.6)	0.32 (S)	**<0.001**
	1 year	0.59 (0.08)	0.59 (0.54, 0.64)	0.02 (0.06)	4.1 (11.0)	0.37 (S)	**<0.001**
	3 years	0.61 (0.08)	0.61 (0.55, 0.65)	0.04 (0.07)	8.2 (14.2)	0.58 (M)	**<0.001**
PEA/AEA	Baseline	24.72 (4.33)	24.21 (22.25, 26.97)	Ref	Ref		
	6 months	24.92 (4.46)	24.41 (22.17, 27.54)	0.25 (3.64)	2.0 (14.8)	0.04 (VS)	0.533
	1 year	25.05 (4.45)	24.75 (22.36, 27.26)	0.57 (4.21)	3.7 (17.2)	0.07 (VS)	0.294
	3 years	23.15 (4.05)	22.83 (20.43, 26.02)	-1.40 (4.58)	-4.1 (17.9)	-0.37 (S)	**0.002**

N=105 at baseline. Proportion of missing values for all compounds is N=0 at baseline, N=3 (2.9%) after 6 months, N=12 (11.4%) after 1 year, and N=19 (18.1%) after 3 years.

Positive/negative values for 6 months, 1 year and 3 years change indicate increase and decrease, respectively, compared to the baseline value. Bold values denote statistical significance at P<0.05.

[1]Effect Size of absolute mean changes: VS= very small (Cohen's d < 0.2); S= small [Cohen's d (0.2–0.5)]; M= moderate [Cohen's d (0.5–0.8)]; L= large [Cohen's d (0.8–1.2)]; VL= very large (Cohen's d ≥ 1.2).

*P-values were obtained from linear mixed effects models adjusted by sex, age, intervention group and diagnostic of type 2 diabetes. Dependent variables were normalized before estimating p-values, using ordered quantile normalization (ORQ) transformation.

N= number. SD= standard deviation. Q1=quartile 1. Q3= quartile 3. 2-AG= 2-arachidonoylglycerol. AEA= anandamide or *N*-arachidonoyl-ethanolamine. DEA= *N*-docosatetraenoylethanolamine. DGLEA= *N*-dihomo-γ-linolenoyl ethanolamide. DHEA= *N*-docosahexaenoylethanolamine. eCBs= endocannabinoids. LEA= *N*-linoleoylethanolamine. OEA= oleoylethanolamide. PEA= palmitoylethanolamide. POEA= *N*-palmitoleoylethanolamine. SEA= *N*-stearoylethanolamine.

Supplementary Table 5. Description of changes in endocannabinoids concentrations from baseline to 6 months, 1 year and 3 years stratified by sex

		Men [N=48]						Women [N=57]						Sex differences			
			Values at each time point		Change from baseline				Values at each time point		Change from baseline			At each time point		In change over time	
Variable	Time	Missing [N (%)]	Mean (SD)	Median (Q1, Q3)	Mean change (SD)	% mean change (SD)	Cohen's d (size)[1]	Missing [N (%)]	Mean (SD)	Median (Q1, Q3)	Mean change (SD)	% mean change (SD)	Cohen's d (size)[1]	Cohen's d (size)[1]	P-value*	Cohen's d (size)[1]	P-value*
AEA (nM)	Baseline	0 (0.0)	0.72 (0.19)	0.72 (0.58, 0.86)	Ref	Ref		0 (0.0)	0.81 (0.26)	0.78 (0.62, 0.97)	Ref	Ref		0.40 (S)	**0.013**		
	6 months	1 (2.1)	0.65 (0.28)	0.59 (0.50, 0.73)	-0.08 (0.22)	-9.6 (25.9)	-0.31 (S)	2 (3.5)	0.78 (0.23)	0.72 (0.63, 0.94)	-0.04 (0.21)	-0.8 (24.1)	-0.13 (VS)	0.54 (M)	**<0.001**	0.19 (S)	**0.047**
	1 year	8 (16.7)	0.70 (0.21)	0.63 (0.55, 0.83)	-0.03 (0.20)	-1.1 (29.7)	-0.13 (VS)	4 (7.0)	0.80 (0.24)	0.77 (0.64, 0.96)	-0.01 (0.26)	4.0 (32.7)	-0.05 (VS)	0.47 (M)	**0.004**	0.10 (VS)	0.544
	3 years	11 (22.9)	0.79 (0.26)	0.71 (0.61, 0.89)	0.07 (0.23)	12.6 (40.7)	0.30 (S)	8 (14.0)	0.87 (0.25)	0.84 (0.68, 0.99)	0.05 (0.30)	13.5 (37.9)	0.20 (S)	0.31 (S)	0.066	-0.05 (VS)	0.992
2-AG (nM)	Baseline	0 (0.0)	5.01 (4.28)	3.83 (2.91, 5.46)	Ref	Ref		0 (0.0)	4.58 (1.99)	3.82 (3.37, 5.58)	Ref	Ref		-0.13 (VS)	0.654		
	6 months	1 (2.1)	4.12 (3.21)	2.89 (2.20, 4.56)	-0.97 (2.82)	-13.2 (40.1)	-0.24 (S)	2 (3.5)	3.78 (1.65)	3.55 (2.71, 4.50)	-0.73 (2.15)	-8.3 (42.9)	-0.44 (S)	-0.14 (VS)	0.815	0.09 (VS)	0.707
	1 year	8 (16.7)	4.20 (4.16)	2.85 (2.26, 4.23)	-0.32 (4.18)	-0.4 (83.3)	-0.19 (S)	4 (7.0)	3.65 (1.32)	3.45 (2.70, 4.16)	-0.90 (2.05)	-9.7 (43.2)	-0.54 (M)	-0.19 (S)	0.483	-0.18 (S)	0.877
	3 years	11 (22.9)	3.58 (1.70)	3.21 (2.35, 4.51)	-0.91 (2.86)	-9.5 (46.7)	-0.42 (S)	8 (14.0)	4.17 (1.61)	3.84 (2.98, 5.30)	-0.52 (2.35)	0.2 (47.9)	-0.22 (S)	0.36 (S)	0.057	0.15 (S)	0.192
DEA (nM)	Baseline	0 (0.0)	0.19 (0.05)	0.18 (0.15, 0.22)	Ref	Ref		0 (0.0)	0.18 (0.04)	0.18 (0.15, 0.20)	Ref	Ref		-0.18 (S)	0.679		
	6 months	1 (2.1)	0.16 (0.06)	0.16 (0.13, 0.19)	-0.03 (0.05)	-12.8 (22.3)	-0.41 (S)	2 (3.5)	0.17 (0.04)	0.17 (0.15, 0.18)	-0.01 (0.03)	-3.4 (18.3)	-0.24 (S)	0.13 (VS)	0.151	0.38 (S)	**0.011**
	1 year	8 (16.7)	0.18 (0.06)	0.16 (0.13, 0.20)	-0.01 (0.05)	-3.8 (23.8)	-0.18 (S)	4 (7.0)	0.18 (0.04)	0.17 (0.16, 0.20)	0.00 (0.04)	0.9 (23.3)	-0.08 (VS)	-0.03 (VS)	0.672	0.18 (S)	0.357
	3 years	11 (22.9)	0.19 (0.05)	0.19 (0.16, 0.21)	0.00 (0.05)	3.1 (22.3)	0.04 (VS)	8 (14.0)	0.18 (0.05)	0.18 (0.15, 0.20)	0.00 (0.04)	3.7 (25.1)	0.12 (VS)	-0.10 (VS)	0.977	0.03 (VS)	0.975
DGLEA (nM)	Baseline	0 (0.0)	0.14 (0.04)	0.14 (0.11, 0.17)	Ref	Ref		0 (0.0)	0.18 (0.06)	0.17 (0.14, 0.21)	Ref	Ref		0.77 (L)	**<0.001**		
	6 months	1 (2.1)	0.13 (0.04)	0.12 (0.10, 0.15)	-0.02 (0.04)	-8.6 (30.1)	-0.38 (S)	2 (3.5)	0.18 (0.05)	0.16 (0.13, 0.20)	-0.01 (0.04)	0.3 (24.0)	-0.10 (VS)	1.03 (L)	**<0.001**	0.24 (S)	0.054
	1 year	8 (16.7)	0.13 (0.04)	0.13 (0.11, 0.15)	-0.01 (0.03)	-1.9 (22.6)	-0.15 (S)	4 (7.0)	0.18 (0.05)	0.17 (0.15, 0.20)	0.00 (0.05)	6.2 (28.6)	-0.02 (VS)	1.00 (L)	**<0.001**	0.20 (S)	0.237
	3 years	11 (22.9)	0.14 (0.04)	0.14 (0.11, 0.16)	0.00 (0.03)	4.3 (29.7)	0.01 (VS)	8 (14.0)	0.19 (0.05)	0.17 (0.16, 0.21)	0.01 (0.05)	9.4 (31.5)	0.11 (VS)	1.05 (L)	**<0.001**	0.11 (VS)	0.372
DHEA (nM)	Baseline	0 (0.0)	1.13 (0.40)	1.07 (0.81, 1.41)	Ref	Ref		0 (0.0)	1.32 (0.44)	1.28 (1.02, 1.52)	Ref	Ref		0.47 (M)	**0.040**		
	6 months	1 (2.1)	1.08 (0.39)	1.08 (0.80, 1.26)	-0.06 (0.33)	-2.5 (26.1)	-0.13 (VS)	2 (3.5)	1.27 (0.35)	1.22 (1.06, 1.47)	-0.05 (0.33)	-0.4 (22.3)	-0.14 (VS)	0.52 (M)	**0.009**	0.03 (VS)	0.508
	1 year	8 (16.7)	1.20 (0.44)	1.12 (0.92, 1.42)	0.07 (0.35)	9.5 (30.6)	0.18 (S)	4 (7.0)	1.29 (0.44)	1.22 (0.97, 1.50)	-0.01 (0.35)	2.4 (25.2)	-0.09 (VS)	0.20 (S)	0.363	-0.21 (S)	0.289
	3 years	11 (22.9)	1.13 (0.34)	1.09 (0.92, 1.39)	0.02 (0.29)	5.9 (29.4)	0.01 (VS)	8 (14.0)	1.36 (0.40)	1.25 (1.10, 1.58)	0.04 (0.36)	7.3 (29.3)	0.08 (VS)	0.60 (M)	**0.017**	0.06 (VS)	0.865
LEA (nM)	Baseline	0 (0.0)	2.09 (0.58)	2.06 (1.64, 2.48)	Ref	Ref		0 (0.0)	2.27 (0.72)	2.13 (1.73, 2.79)	Ref	Ref		0.27 (S)	0.146		
	6 months	1 (2.1)	1.88 (0.60)	1.79 (1.51, 2.13)	-0.20 (0.54)	-7.3 (23.0)	-0.34 (S)	2 (3.5)	2.16 (0.61)	2.04 (1.73, 2.49)	-0.11 (0.63)	-0.1 (28.5)	-0.15 (S)	0.46 (M)	**0.005**	0.15 (S)	0.166
	1 year	8 (16.7)	2.08 (0.56)	2.07 (1.69, 2.37)	0.02 (0.46)	3.1 (24.4)	-0.01 (VS)	4 (7.0)	2.21 (0.53)	2.15 (1.88, 2.52)	-0.02 (0.67)	5.1 (29.0)	-0.08 (VS)	0.25 (S)	0.116	-0.07 (VS)	0.922
	3 years	11 (22.9)	2.17 (0.58)	2.08 (1.81, 2.50)	0.13 (0.52)	8.7 (27.5)	0.15 (VS)	8 (14.0)	2.36 (0.69)	2.28 (1.85, 2.87)	0.08 (0.75)	9.1 (31.8)	0.13 (VS)	0.29 (S)	0.172	-0.07 (VS)	0.952
OEA (nM)	Baseline	0 (0.0)	9.78 (2.19)	9.72 (8.02, 11.15)	Ref	Ref		0 (0.0)	10.77 (2.63)	10.59 (9.22, 12.72)	Ref	Ref		0.40 (S)	**0.042**		
	6 months	1 (2.1)	9.36 (2.73)	8.69 (7.58, 10.89)	-0.43 (2.33)	-2.6 (23.0)	-0.17 (S)	2 (3.5)	10.52 (2.22)	10.61 (9.06, 12.03)	-0.29 (2.05)	0.2 (19.2)	-0.10 (VS)	0.47 (M)	**0.006**	0.06 (VS)	0.436
	1 year	8 (16.7)	10.23 (2.27)	9.97 (8.68, 11.43)	0.30 (2.28)	5.9 (26.0)	0.20 (S)	4 (7.0)	10.99 (2.44)	10.87 (9.87, 12.57)	0.25 (2.49)	5.9 (24.7)	0.09 (VS)	0.32 (S)	0.078	-0.02 (VS)	0.870
	3 years	11 (22.9)	10.68 (2.54)	10.07 (9.15, 11.42)	0.93 (2.62)	13.3 (33.8)	0.38 (S)	8 (14.0)	11.64 (2.85)	11.12 (9.35, 13.66)	0.84 (2.76)	11.6 (28.5)	0.32 (S)	0.35 (S)	0.130	-0.03 (VS)	0.905
PEA (nM)	Baseline	0 (0.0)	17.82 (3.63)	17.88 (15.30, 19.93)	Ref	Ref		0 (0.0)	18.87 (4.03)	18.54 (15.95, 21.86)	Ref	Ref		0.27 (S)	0.176		
	6 months	1 (2.1)	16.09 (3.86)	15.55 (13.85, 17.44)	-1.77 (3.63)	-8.5 (19.2)	-0.46 (M)	2 (3.5)	18.02 (3.56)	17.76 (15.84, 19.88)	-0.87 (3.03)	-2.8 (16.7)	-0.22 (S)	0.52 (M)	**0.003**	0.27 (S)	0.085
	1 year	8 (16.7)	17.46 (3.70)	17.09 (14.83, 19.72)	-0.32 (2.90)	-0.8 (16.5)	-0.10 (VS)	4 (7.0)	18.77 (3.93)	18.20 (16.11, 21.64)	0.12 (3.86)	3.0 (21.7)	-0.03 (VS)	0.34 (S)	**0.047**	0.12 (VS)	0.560
	3 years	11 (22.9)	17.64 (3.79)	16.84 (14.81, 19.62)	0.09 (3.81)	2.5 (21.8)	-0.05 (VS)	8 (14.0)	19.18 (3.63)	18.83 (16.86, 21.68)	0.34 (3.75)	4.4 (20.9)	0.08 (VS)	0.42 (S)	0.053	0.07 (VS)	0.524
POEA (nM)	Baseline	0 (0.0)	1.56 (0.70)	1.41 (1.02, 1.99)	Ref	Ref		0 (0.0)	2.12 (0.91)	1.93 (1.50, 2.72)	Ref	Ref		0.67 (M)	**0.002**		
	6 months	1 (2.1)	1.35 (0.52)	1.31 (1.00, 1.64)	-0.22 (0.59)	-4.4 (41.2)	-0.35 (S)	2 (3.5)	1.99 (0.82)	1.95 (1.40, 2.47)	-0.16 (0.69)	1.1 (39.5)	-0.14 (VS)	0.91 (L)	**<0.001**	0.10 (VS)	0.299
	1 year	8 (16.7)	1.49 (0.50)	1.33 (1.18, 1.90)	-0.05 (0.51)	5.4 (34.3)	-0.11 (VS)	4 (7.0)	2.07 (0.83)	1.86 (1.50, 2.72)	-0.05 (0.82)	9.0 (51.2)	-0.05 (VS)	0.81 (L)	**<0.001**	0.00 (VS)	0.978
	3 years	11 (22.9)	1.57 (1.04)	1.39 (1.02, 1.62)	0.04 (0.81)	7.8 (46.5)	0.01 (VS)	8 (14.0)	2.11 (0.96)	2.04 (1.54, 2.54)	0.01 (0.85)	9.8 (44.3)	0.00 (VS)	0.54 (M)	**0.020**	-0.03 (VS)	0.864
SEA (nM)	Baseline	0 (0.0)	4.41 (0.88)	4.32 (3.82, 4.93)	Ref	Ref		0 (0.0)	5.00 (1.10)	4.79 (4.25, 5.81)	Ref	Ref		0.59 (M)	**0.003**		
	6 months	1 (2.1)	3.87 (0.79)	3.79 (3.30, 4.39)	-0.57 (0.75)	-11.7 (15.2)	-0.65 (M)	2 (3.5)	4.74 (0.98)	4.69 (4.12, 5.21)	-0.24 (0.85)	-3.0 (17.2)	-0.25 (S)	0.97 (L)	**<0.001**	0.41 (S)	**0.020**
	1 year	8 (16.7)	4.30 (1.07)	4.22 (3.59, 4.78)	-0.11 (0.87)	-1.7 (18.5)	-0.12 (VS)	4 (7.0)	5.02 (1.10)	4.85 (4.14, 5.73)	0.09 (0.95)	3.5 (18.4)	0.01 (VS)	0.66 (M)	**0.001**	0.21 (S)	0.226
	3 years	11 (22.9)	4.35 (0.83)	4.34 (3.58, 5.00)	-0.06 (0.84)	0.3 (17.7)	-0.08 (VS)	8 (14.0)	5.13 (1.11)	5.18 (4.33, 5.71)	0.11 (0.99)	3.9 (18.9)	0.11 (VS)	0.78 (L)	**<0.001**	0.19 (S)	0.397
DHEA/AEA	Baseline	0 (0.0)	1.64 (0.64)	1.54 (1.12, 2.01)	Ref	Ref		0 (0.0)	1.69 (0.47)	1.63 (1.32, 1.94)	Ref	Ref		0.08 (VS)	0.753		
	6 months	1 (2.1)	1.79 (0.75)	1.62 (1.26, 2.12)	0.13 (0.49)	11.5 (29.4)	0.21 (S)	2 (3.5)	1.66 (0.37)	1.73 (1.40, 1.89)	0.00 (0.33)	2.3 (17.7)	-0.05 (VS)	-0.22 (S)	0.078	-0.33 (S)	0.119
	1 year	8 (16.7)	1.83 (0.74)	1.63 (1.37, 2.20)	0.19 (0.55)	16.8 (38.2)	0.27 (S)	4 (7.0)	1.66 (0.49)	1.65 (1.25, 1.91)	0.01 (0.47)	3.6 (27.4)	-0.05 (VS)	-0.27 (S)	0.074	-0.35 (S)	0.107
	3 years	11 (22.9)	1.53 (0.55)	1.49 (1.12, 1.71)	-0.09 (0.45)	-1.5 (25.7)	-0.18 (S)	8 (14.0)	1.63 (0.45)	1.69 (1.30, 1.94)	-0.05 (0.43)	-0.2 (27.6)	-0.12 (VS)	0.19 (S)	0.848	0.08 (VS)	0.649
OEA/AEA	Baseline	0 (0.0)	13.90 (2.23)	13.55 (12.42, 15.52)	Ref	Ref		0 (0.0)	13.61 (2.35)	13.32 (12.08, 14.78)	Ref	Ref		-0.13 (VS)	0.142		
	6 months	1 (2.1)	15.12 (2.71)	14.95 (13.40, 16.67)	1.25 (2.02)	9.7 (14.2)	0.49 (M)	2 (3.5)	13.88 (2.48)	13.11 (12.10, 15.24)	0.27 (1.36)	2.5 (10.1)	0.11 (VS)	-0.48 (M)	**<0.001**	-0.58 (M)	**0.005**
	1 year	8 (16.7)	15.25 (2.79)	15.16 (13.08, 17.26)	1.27 (2.37)	10.0 (17.3)	0.54 (M)	4 (7.0)	14.19 (2.78)	14.02 (12.08, 15.81)	0.57 (1.86)	4.8 (14.2)	0.23 (S)	-0.38 (S)	**0.012**	-0.34 (S)	0.104
	3 years	11 (22.9)	14.16 (2.79)	13.40 (12.24, 16.24)	0.38 (2.15)	3.0 (14.9)	0.10 (VS)	8 (14.0)	13.79 (2.72)	13.87 (12.03, 15.73)	0.12 (2.06)	1.6 (15.7)	0.07 (VS)	-0.13 (VS)	0.151	-0.12 (VS)	0.713
OEA/PEA	Baseline	0 (0.0)	0.55 (0.07)	0.56 (0.50, 0.60)	Ref	Ref		0 (0.0)	0.57 (0.07)	0.57 (0.54, 0.61)	Ref	Ref		0.26 (S)	0.191		
	6 months	1 (2.1)	0.58 (0.07)	0.59 (0.53, 0.62)	0.03 (0.06)	6.3 (10.6)	0.43 (S)	2 (3.5)	0.59 (0.07)	0.58 (0.54, 0.63)	0.02 (0.05)	3.0 (8.5)	0.24 (S)	0.07 (VS)	0.704	-0.31 (S)	0.180
	1 year	8 (16.7)	0.59 (0.08)	0.60 (0.54, 0.65)	0.03 (0.07)	6.1 (13.2)	0.51 (M)	4 (7.0)	0.59 (0.08)	0.59 (0.54, 0.63)	0.01 (0.05)	2.7 (9.0)	0.24 (S)	-0.04 (VS)	0.824	-0.28 (S)	0.157
	3 years	11 (22.9)	0.61 (0.07)	0.62 (0.57, 0.65)	0.05 (0.09)	10.5 (17.0)	0.77 (L)	8 (14.0)	0.61 (0.09)	0.61 (0.54, 0.65)	0.03 (0.06)	6.4 (11.6)	0.45 (S)	0.00 (VS)	0.829	-0.20 (S)	0.285
PEA/AEA	Baseline	0 (0.0)	25.39 (3.80)	24.82 (23.23, 27.06)	Ref	Ref		0 (0.0)	24.17 (4.69)	23.35 (21.03, 26.77)	Ref	Ref		-0.28 (S)	**0.021**		
	6 months	1 (2.1)	26.18 (4.57)	25.48 (23.19, 28.71)	0.83 (3.96)	4.0 (15.9)	0.19 (S)	2 (3.5)	23.85 (4.10)	23.30 (21.36, 26.02)	-0.24 (3.29)	0.3 (13.7)	-0.07 (VS)	-0.54 (M)	**<0.001**	-0.29 (S)	0.178
	1 year	8 (16.7)	26.00 (4.32)	25.48 (23.48, 28.26)	0.88 (4.65)	4.8 (18.5)	0.15 (S)	4 (7.0)	24.33 (4.45)	24.09 (21.53, 26.59)	0.33 (3.88)	2.8 (16.3)	0.04 (VS)	-0.38 (S)	**0.013**	-0.13 (VS)	0.672
	3 years	11 (22.9)	23.48 (4.59)	22.83 (20.28, 26.31)	-1.46 (4.51)	-5.2 (16.2)	-0.46 (M)	8 (14.0)	22.89 (3.62)	22.83 (20.84, 25.76)	-1.34 (4.67)	-3.3 (19.2)	-0.30 (S)	-0.14 (VS)	0.160	0.03 (VS)	0.430

Positive/negative values for 6 months, 1 year and 3 years change indicate increase and decrease, respectively, compared to the baseline value.

P-values of cross-sectional differences were obtained from the analysis of variance (ANOVA) from multivariable-adjusted linear models. P-values of differences in the rate of change were obtained from linear mixed effects models. All the analyses were adjusted by intervention group, age, and diagnostic of type 2 diabetes. Dependent variables were normalized before estimating p-values, using ordered quantile normalization (ORQ) transformation.

N=number. 95%CI= 95% confidence intervals

[1]Effect Size: VS = very small (Cohen's d < 0.2); S = small [Cohen's d (0.2–0.5)]; M = medium [Cohen's d (0.5–0.8)]; L = large [Cohen's d (0.8–1.2)]; VL = very large (Cohen's d ≥ 1.2).

Bold values denote statistical significance at P<0.05.

Supplementary Table 6. Change in the ratios eCB/fatty acids from baseline to 6 months, 1 year and 3 years by sex

		Men [N=48]					Women [N=57]					Sex differences			
			Values at each time point		Change from baseline			Values at each time point		Change from baseline		At each time point		In change over time	
Compound (‰)	Time	Missing [N (%)]	Mean (SD)	Median (Q1, Q3)	Mean change (SD)	Cohen's d (size)[1]	Missing [N (%)]	Mean (SD)	Median (Q1, Q3)	Mean change (SD)	Cohen's d (size)[1]	Cohen's d (size)[1]	P-value*	Cohen's d (size)[1]	P-value*
AEA/C20:4n6	Baseline	0 (0.0)	1.3 (0.5)	1.3 (1.0, 1.6)	Ref		0 (0.0)	1.6 (0.6)	1.6 (1.1, 1.9)	Ref		0.49 (M)	**0.010**		
	6 months	1 (2.1)	1.2 (0.5)	1.1 (0.9, 1.5)	-0.1 (0.5)	-0.20 (S)	2 (3.5)	1.5 (0.5)	1.3 (1.1, 1.8)	-0.1 (0.6)	-0.20 (S)	0.48 (M)	**0.005**	0.00 (VS)	0.881
	1 year	8 (16.7)	1.2 (0.5)	1.2 (0.9, 1.5)	-0.1 (0.6)	-0.19 (S)	4 (7.0)	1.5 (0.6)	1.5 (1.0, 1.8)	-0.1 (0.7)	-0.13 (VS)	0.47 (M)	**0.027**	0.10 (VS)	0.876
	3 years	11 (22.9)	1.4 (0.5)	1.4 (1.1, 1.8)	0.1 (0.6)	0.27 (S)	8 (14.0)	1.7 (0.7)	1.6 (1.2, 1.9)	0.1 (0.7)	0.15 (VS)	0.34 (S)	0.090	-0.07 (VS)	0.638
2-AG/C20:4n6	Baseline	0 (0.0)	8.6 (6.1)	6.8 (5.2, 10.2)	Ref		0 (0.0)	8.7 (4.0)	7.9 (5.5, 10.5)	Ref		0.02 (VS)	0.831		
	6 months	1 (2.1)	7.8 (6.2)	6.0 (3.9, 10.5)	-0.9 (4.8)	-0.13 (VS)	2 (3.5)	7.1 (3.5)	6.0 (4.8, 8.4)	-1.4 (4.5)	-0.45 (S)	-0.16 (S)	0.618	-0.11 (VS)	0.526
	1 year	8 (16.7)	7.4 (9.6)	5.6 (4.2, 7.8)	-0.8 (9.5)	-0.16 (S)	4 (7.0)	6.6 (3.0)	6.1 (4.7, 8.3)	-1.9 (4.0)	-0.59 (M)	-0.11 (VS)	0.622	-0.16 (S)	0.865
	3 years	11 (22.9)	6.8 (4.0)	5.4 (3.9, 9.7)	-1.3 (5.2)	-0.35 (S)	8 (14.0)	8.0 (3.9)	7.3 (5.4, 10.3)	-1.1 (5.3)	-0.19 (S)	0.31 (S)	0.130	0.04 (VS)	0.537
DGLEA/C20:3n6	Baseline	0 (0.0)	2.7 (1.0)	2.3 (2.0, 3.4)	Ref		0 (0.0)	3.6 (1.3)	3.6 (2.7, 4.4)	Ref		0.81 (L)	**<0.001**		
	6 months	1 (2.1)	2.8 (1.2)	2.5 (2.0, 3.6)	0.2 (1.1)	0.15 (VS)	2 (3.5)	3.5 (1.3)	3.3 (2.7, 4.1)	-0.1 (1.3)	-0.09 (VS)	0.52 (M)	**0.004**	-0.19 (S)	0.265
	1 year	8 (16.7)	2.7 (1.2)	2.5 (2.0, 3.4)	-0.1 (1.2)	0.04 (VS)	4 (7.0)	3.6 (1.6)	3.4 (2.8, 4.5)	0.1 (1.6)	0.01 (VS)	0.62 (M)	**0.004**	0.09 (VS)	0.981
	3 years	11 (22.9)	3.1 (1.1)	3.3 (2.1, 3.7)	0.3 (1.2)	0.37 (S)	8 (14.0)	3.9 (1.8)	3.6 (2.9, 4.7)	0.3 (1.8)	0.20 (S)	0.56 (M)	**0.007**	-0.06 (VS)	0.454
DHEA/C22:6n3	Baseline	0 (0.0)	7.3 (3.4)	6.8 (5.1, 9.3)	Ref		0 (0.0)	8.4 (3.3)	8.7 (5.8, 10.0)	Ref		0.31 (S)	0.159		
	6 months	1 (2.1)	6.6 (2.7)	6.0 (4.8, 8.2)	-0.7 (3.2)	-0.23 (S)	2 (3.5)	7.6 (2.7)	7.1 (5.8, 9.3)	-0.7 (3.6)	-0.27 (S)	0.34 (S)	0.077	0.01 (VS)	0.897
	1 year	8 (16.7)	7.0 (3.3)	6.4 (5.1, 8.3)	-0.6 (3.6)	-0.09 (VS)	4 (7.0)	7.6 (2.9)	7.4 (5.9, 8.9)	-0.6 (4.0)	-0.27 (S)	0.17 (S)	0.544	0.01 (VS)	0.762
	3 years	11 (22.9)	7.0 (2.4)	7.3 (5.7, 8.2)	-0.5 (3.3)	-0.12 (VS)	8 (14.0)	8.6 (4.2)	7.9 (6.0, 10.1)	0.0 (5.0)	0.05 (VS)	0.44 (S)	0.057	0.12 (VS)	0.956
LEA/C18:2n6	Baseline	0 (0.0)	4.8 (1.7)	4.7 (3.3, 6.0)	Ref		0 (0.0)	5.7 (1.9)	5.7 (4.3, 7.2)	Ref		0.50 (M)	**0.015**		
	6 months	1 (2.1)	4.8 (2.0)	4.7 (3.1, 5.7)	0.0 (1.7)	0.00 (VS)	2 (3.5)	5.4 (1.9)	5.3 (4.0, 6.3)	-0.2 (1.9)	-0.15 (S)	0.33 (S)	**0.049**	-0.14 (VS)	0.551
	1 year	8 (16.7)	4.9 (2.0)	4.4 (3.9, 6.2)	-0.1 (2.0)	0.05 (VS)	4 (7.0)	5.9 (2.3)	5.5 (4.6, 7.2)	0.3 (2.3)	0.08 (VS)	0.47 (M)	**0.032**	0.18 (S)	0.612
	3 years	11 (22.9)	5.8 (2.2)	5.7 (4.2, 7.3)	0.8 (2.2)	0.50 (M)	8 (14.0)	6.3 (2.8)	5.8 (4.2, 8.0)	0.4 (2.6)	0.24 (S)	0.20 (S)	**0.331**	-0.16 (S)	0.262
OEA/C18:1n9	Baseline	0 (0.0)	14.7 (5.2)	15.0 (10.0, 18.7)	Ref		0 (0.0)	18.7 (6.5)	18.2 (14.3, 24.3)	Ref		0.68 (M)	**0.002**		
	6 months	1 (2.1)	15.7 (6.1)	14.6 (11.1, 19.8)	1.0 (4.9)	0.18 (S)	2 (3.5)	18.1 (6.4)	17.2 (13.7, 22.5)	-0.4 (6.0)	-0.09 (VS)	0.38 (S)	**0.039**	-0.25 (S)	0.141
	1 year	8 (16.7)	16.4 (6.9)	15.6 (12.2, 21.0)	1.1 (6.7)	0.28 (S)	4 (7.0)	19.7 (7.9)	19.0 (15.6, 23.2)	1.3 (7.4)	0.14 (VS)	0.44 (S)	0.058	0.02 (VS)	0.778
	3 years	11 (22.9)	18.6 (6.6)	18.7 (13.7, 23.0)	3.5 (6.3)	0.68 (M)	8 (14.0)	21.2 (10.3)	20.1 (13.6, 26.2)	1.9 (9.3)	0.29 (S)	0.29 (S)	0.225	-0.19 (S)	0.103
PEA/C16:0	Baseline	0 (0.0)	18.2 (5.5)	19.4 (14.5, 22.2)	Ref		0 (0.0)	22.6 (7.0)	23.4 (17.3, 28.3)	Ref		0.69 (M)	**0.002**		
	6 months	1 (2.1)	18.6 (6.0)	18.6 (13.8, 22.4)	0.3 (5.3)	0.06 (VS)	2 (3.5)	21.7 (7.2)	20.6 (16.9, 25.3)	-0.6 (7.0)	-0.13 (VS)	0.46 (M)	**0.015**	-0.15 (VS)	0.313
	1 year	8 (16.7)	19.4 (8.4)	18.0 (15.1, 22.6)	0.6 (8.1)	0.17 (S)	4 (7.0)	23.6 (9.2)	23.8 (19.0, 26.8)	1.5 (9.0)	0.13 (VS)	0.48 (M)	**0.042**	0.11 (VS)	0.919
	3 years	11 (22.9)	22.2 (7.6)	21.2 (17.3, 27.0)	3.4 (7.8)	0.61 (M)	8 (14.0)	25.0 (12.1)	23.9 (19.2, 27.9)	1.8 (11.7)	0.25 (S)	0.27 (S)	0.231	-0.15 (S)	0.146
POEA/C16:1n7	Baseline	0 (0.0)	120.6 (75.5)	116.2 (59.3, 149.8)	Ref		0 (0.0)	160.1 (83.8)	156.1 (78.8, 228.5)	Ref		0.49 (M)	**0.032**		
	6 months	1 (2.1)	148.9 (95.6)	133.8 (72.1, 203.4)	31.4 (80.4)	0.33 (S)	2 (3.5)	172.4 (96.3)	153.0 (96.9, 227.8)	12.9 (82.8)	0.14 (VS)	0.24 (S)	0.245	-0.23 (S)	0.134
	1 year	8 (16.7)	183.2 (143.4)	153.9 (89.6, 234.6)	53.0 (135.0)	0.56 (M)	4 (7.0)	198.1 (126.2)	176.7 (116.7, 253.2)	39.2 (110.1)	0.36 (S)	0.11 (VS)	0.765	-0.11 (VS)	0.355
	3 years	11 (22.9)	197.7 (118.2)	171.5 (130.4, 257.6)	71.5 (104.0)	0.80 (L)	8 (14.0)	200.7 (166.2)	169.6 (86.1, 249.9)	36.3 (148.5)	0.32 (S)	0.02 (VS)	0.983	-0.27 (S)	**0.014**
SEA/C18:0	Baseline	0 (0.0)	6.3 (1.7)	6.6 (5.2, 7.4)	Ref		0 (0.0)	7.9 (2.3)	7.8 (6.2, 9.9)	Ref		0.79 (L)	**<0.001**		
	6 months	1 (2.1)	6.0 (1.7)	6.3 (5.0, 7.1)	-0.3 (1.6)	-0.15 (VS)	2 (3.5)	7.4 (2.4)	6.8 (6.0, 8.4)	-0.4 (2.5)	-0.23 (S)	0.62 (M)	**0.001**	-0.05 (VS)	0.515
	1 year	8 (16.7)	6.3 (2.6)	6.0 (4.8, 7.6)	-0.1 (2.5)	-0.01 (VS)	4 (7.0)	8.0 (3.0)	7.8 (6.5, 9.0)	0.3 (3.0)	0.02 (VS)	0.60 (M)	**0.010**	0.14 (VS)	0.645
	3 years	11 (22.9)	7.0 (2.3)	7.1 (5.2, 8.6)	0.6 (2.3)	0.37 (S)	8 (14.0)	8.5 (3.3)	8.0 (6.4, 10.0)	0.3 (3.7)	0.19 (S)	0.49 (M)	**0.026**	-0.09 (VS)	0.470

Positive/negative values for 6 months, 1 year and 3 years change indicate increase and decrease, respectively, compared to the baseline value.
P-values of cross-sectional differences were obtained from the analysis of variance (ANOVA) from multivariable-adjusted linear models. P-values of differences in the rate of change were obtained from linear mixed effects models. All the analyses were adjusted by intervention group, age, and diagnostic of type 2 diabetes. Dependent variables were normalized before estimating p-values, using ordered quantile normalization (ORQ) transformation.
N=number. 95%CI= 95% confidence intervals
[1]Effect Size: VS = very small (Cohen's d < 0.2); S = small [Cohen's d (0.2–0.5)]; M = medium [Cohen's d (0.5–0.8)]; L = large [Cohen's d (0.8–1.2)]; VL = very large (Cohen's d ≥ 1.2).
Bold values denote statistical significance at P<0.05.

Supplementary Table 7. Description of 6-months changes in endocannabinoids and intervention factors according to the achievement of 8% weight loss after 6 months (respondents/non-respondents)

				NON-RESPONDENTS *(do not achieve 8% weight reductions after 6 months)* *[N=27 men and N=44 women]*				RESPONDENTS *(achieve 8% weight reductions after 6 months)* *[N=21 men and N=13 women]*				Between-group differences	
**Sex**	**Factor**	**Compound**	**Time**	**Mean (SD)**	**Median (Q1, Q3)**	**Mean change (SD)**	**% mean change**	**Mean (SD)**	**Median (Q1, Q3)**	**Mean change (SD)**	**% mean change**	**At each time point**	**In change over time**
Men [N=48]	eCBs	AEA (nM)	Baseline	0.72 (0.21)	0.73 (0.56, 0.85)	Ref	Ref	0.72 (0.18)	0.70 (0.59, 0.88)	Ref	Ref	0.373	
			6 months	0.66 (0.34)	0.57 (0.48, 0.71)	-0.06 (0.25)	-9.1 (28.2)	0.64 (0.16)	0.67 (0.52, 0.74)	-0.09 (0.17)	-10.4 (23.3)	0.262	0.945
		2-AG (nM)	Baseline	5.57 (4.72)	4.42 (3.01, 5.83)	Ref	Ref	4.30 (3.62)	3.57 (2.68, 4.51)	Ref	Ref	0.240	
			6 months	4.98 (3.90)	3.53 (2.37, 6.69)	-0.58 (2.84)	-6.0 (45.9)	2.96 (1.28)	2.69 (2.09, 3.32)	-1.49 (2.78)	-23.0 (28.9)	0.258	0.093
		DEA (nM)	Baseline	0.19 (0.06)	0.17 (0.14, 0.22)	Ref	Ref	0.19 (0.05)	0.18 (0.15, 0.22)	Ref	Ref	0.409	
			6 months	0.17 (0.08)	0.16 (0.12, 0.20)	-0.02 (0.05)	-10.1 (23.8)	0.16 (0.04)	0.16 (0.14, 0.18)	-0.03 (0.04)	-16.6 (20.0)	0.651	0.414
		DGLEA (nM)	Baseline	0.15 (0.04)	0.14 (0.11, 0.18)	Ref	Ref	0.14 (0.03)	0.13 (0.11, 0.15)	Ref	Ref	0.740	
			6 months	0.13 (0.05)	0.13 (0.10, 0.16)	-0.01 (0.04)	-4.9 (34.1)	0.11 (0.03)	0.11 (0.10, 0.13)	-0.02 (0.03)	-13.7 (23.6)	0.477	0.335
		DHEA (nM)	Baseline	1.18 (0.45)	1.09 (0.82, 1.43)	Ref	Ref	1.06 (0.34)	0.99 (0.80, 1.28)	Ref	Ref	0.298	
			6 months	1.07 (0.43)	0.95 (0.78, 1.24)	-0.11 (0.38)	-5.9 (27.6)	1.08 (0.32)	1.09 (0.89, 1.26)	0.00 (0.25)	2.1 (24.0)	0.486	0.191
		DHEA/AEA	Baseline	1.73 (0.69)	1.63 (1.08, 2.14)	Ref	Ref	1.54 (0.57)	1.37 (1.13, 1.97)	Ref	Ref	0.202	
			6 months	1.80 (0.83)	1.44 (1.22, 2.11)	0.08 (0.58)	7.1 (29.6)	1.77 (0.63)	1.73 (1.31, 2.10)	0.21 (0.34)	17.5 (28.7)	0.797	0.205
		LEA (nM)	Baseline	2.17 (0.68)	2.14 (1.57, 2.63)	Ref	Ref	1.98 (0.43)	1.88 (1.69, 2.26)	Ref	Ref	0.895	
			6 months	1.96 (0.68)	1.84 (1.59, 2.25)	-0.21 (0.64)	-6.9 (25.0)	1.79 (0.47)	1.65 (1.49, 2.04)	-0.18 (0.37)	-7.9 (20.5)	0.679	0.914
		OEA (nM)	Baseline	9.85 (2.43)	9.68 (7.65, 11.33)	Ref	Ref	9.70 (1.88)	9.96 (8.64, 10.59)	Ref	Ref	0.970	
			6 months	9.25 (3.30)	8.45 (7.40, 10.75)	-0.60 (2.65)	-4.8 (24.0)	9.52 (1.76)	9.54 (8.39, 11.09)	-0.21 (1.86)	0.3 (21.9)	0.175	0.350
		OEA/AEA	Baseline	14.04 (2.33)	13.66 (12.61, 15.72)	Ref	Ref	13.72 (2.13)	13.18 (12.25, 14.92)	Ref	Ref	0.437	
			6 months	14.89 (2.70)	14.84 (13.32, 16.53)	0.85 (1.92)	6.6 (13.2)	15.43 (2.76)	15.35 (13.40, 16.60)	1.80 (2.08)	13.7 (14.9)	0.971	0.077
		OEA/PEA	Baseline	0.56 (0.09)	0.55 (0.49, 0.62)	Ref	Ref	0.55 (0.05)	0.56 (0.51, 0.59)	Ref	Ref	0.873	
			6 months	0.57 (0.08)	0.57 (0.52, 0.60)	0.01 (0.05)	2.7 (10.2)	0.60 (0.05)	0.62 (0.56, 0.63)	0.06 (0.05)	11.2 (9.3)	0.091	**0.002**
		PEA (nM)	Baseline	17.73 (3.48)	18.18 (15.20, 19.80)	Ref	Ref	17.93 (3.91)	17.31 (15.70, 20.14)	Ref	Ref	0.657	
			6 months	16.24 (4.40)	15.52 (14.03, 17.70)	-1.49 (4.10)	-7.4 (21.1)	15.89 (3.08)	15.85 (13.62, 17.13)	-2.14 (2.93)	-10.0 (16.6)	0.723	0.620
		PEA/AEA	Baseline	25.49 (3.87)	25.84 (23.36, 27.01)	Ref	Ref	25.25 (3.81)	24.39 (22.97, 27.06)	Ref	Ref	0.621	
			6 months	26.53 (4.63)	27.37 (23.19, 29.62)	1.03 (3.95)	4.7 (15.8)	25.71 (4.56)	24.41 (23.06, 26.86)	0.55 (4.06)	3.0 (16.3)	0.104	0.572
		POEA (nM)	Baseline	1.56 (0.72)	1.37 (0.90, 1.93)	Ref	Ref	1.57 (0.70)	1.44 (1.10, 1.98)	Ref	Ref	0.928	
			6 months	1.36 (0.59)	1.25 (0.98, 1.71)	-0.20 (0.65)	-4.0 (41.8)	1.34 (0.43)	1.35 (1.13, 1.56)	-0.24 (0.52)	-5.0 (41.3)	0.854	0.995
		SEA (nM)	Baseline	4.40 (0.85)	4.33 (3.91, 4.74)	Ref	Ref	4.43 (0.95)	4.24 (3.71, 5.21)	Ref	Ref	0.826	
			6 months	3.94 (0.85)	3.79 (3.37, 4.59)	-0.46 (0.81)	-9.7 (16.1)	3.78 (0.71)	3.77 (3.27, 4.28)	-0.71 (0.66)	-14.3 (14.0)	0.563	0.260
	Intervention factors	MedDiet adherence	Baseline	6.5 (2.6)	6.0 (5.0, 8.5)	Ref		7.1 (2.7)	7.0 (5.0, 9.0)	Ref		0.708	
			6 months	10.4 (2.6)	11.0 (9.0, 12.0)	3.9 (2.7)		12.4 (2.4)	13.0 (11.0, 15.0)	5.2 (3.5)		**0.046**	0.129
		Physical activity (METs x min/week)	Baseline	2655.3 (2579.1)	1678.3 (512.8, 4717.9)	Ref		2839.3 (2160.6)	2237.8 (1174.8, 3524.5)	Ref		0.286	
			6 months	3005.0 (2066.9)	2517.5 (1711.0, 3465.0)	349.7 (2171.4)		3854.1 (2105.1)	3230.8 (2545.4, 5035.0)	1014.8 (2114.6)		0.417	0.889
		Total energy intake (Kcal/day)	Baseline	2518.9 (586.4)	2443.2 (2061.1, 2758.9)	Ref		2499.7 (860.9)	2253.4 (1971.0, 2926.5)	Ref		0.628	
			6 months	2290.8 (338.3)	2236.1 (2135.5, 2482.2)	-228.1 (647.0)		2322.2 (320.1)	2266.4 (2146.6, 2474.2)	-177.5 (883.3)		0.145	0.558
Women [N=57]	eCBs	AEA (nM)	Baseline	0.84 (0.29)	0.77 (0.62, 0.99)	Ref	Ref	0.73 (0.15)	0.79 (0.69, 0.84)	Ref	Ref	0.134	
			6 months	0.83 (0.23)	0.79 (0.67, 0.99)	-0.02 (0.23)	2.6 (25.4)	0.64 (0.15)	0.66 (0.59, 0.67)	-0.09 (0.11)	-11.8 (15.3)	**0.003**	**0.055**
		2-AG (nM)	Baseline	4.63 (2.12)	3.77 (3.33, 5.15)	Ref	Ref	4.43 (1.55)	3.82 (3.56, 5.76)	Ref	Ref	0.724	
			6 months	3.66 (1.28)	3.56 (2.68, 4.46)	-0.88 (1.92)	-11.3 (32.8)	4.17 (2.54)	3.51 (2.86, 4.93)	-0.26 (2.81)	1.4 (66.8)	0.669	0.742
		DEA (nM)	Baseline	0.18 (0.04)	0.17 (0.15, 0.20)	Ref	Ref	0.18 (0.03)	0.19 (0.15, 0.21)	Ref	Ref	0.279	
			6 months	0.18 (0.04)	0.17 (0.15, 0.19)	-0.01 (0.04)	-0.3 (19.6)	0.15 (0.03)	0.16 (0.14, 0.17)	-0.02 (0.01)	-13.4 (6.8)	**0.003**	**0.024**
		DGLEA (nM)	Baseline	0.19 (0.07)	0.17 (0.14, 0.22)	Ref	Ref	0.16 (0.03)	0.17 (0.14, 0.18)	Ref	Ref	0.166	
			6 months	0.18 (0.06)	0.18 (0.15, 0.22)	0.00 (0.05)	3.5 (25.7)	0.14 (0.03)	0.13 (0.12, 0.16)	-0.02 (0.02)	-10.1 (13.2)	**0.004**	0.117
		DHEA (nM)	Baseline	1.34 (0.47)	1.27 (1.03, 1.56)	Ref	Ref	1.25 (0.29)	1.29 (1.02, 1.45)	Ref	Ref	0.642	
			6 months	1.31 (0.36)	1.27 (1.07, 1.50)	-0.03 (0.36)	2.6 (23.3)	1.12 (0.28)	1.15 (0.93, 1.29)	-0.13 (0.21)	-9.9 (16.2)	0.263	0.104
		DHEA/AEA	Baseline	1.66 (0.48)	1.59 (1.30, 1.89)	Ref	Ref	1.76 (0.45)	1.76 (1.57, 1.96)	Ref	Ref	0.151	
			6 months	1.63 (0.38)	1.61 (1.32, 1.87)	-0.01 (0.31)	1.8 (17.8)	1.77 (0.33)	1.81 (1.66, 1.96)	0.01 (0.39)	3.8 (18.0)	0.063	0.707
		LEA (nM)	Baseline	2.27 (0.76)	2.12 (1.77, 2.83)	Ref	Ref	2.25 (0.63)	2.23 (1.72, 2.42)	Ref	Ref	0.387	

		6 months	2.23 (0.64)	2.15 (1.74, 2.50)	-0.05 (0.67)	3.6 (30.7)	1.94 (0.48)	1.92 (1.58, 2.02)	-0.31 (0.42)	-12.0 (15.5)	**0.015**	0.087
	OEA (nM)	Baseline	10.84 (2.79)	10.84 (9.33, 13.02)	Ref	Ref	10.51 (2.11)	9.88 (9.09, 10.93)	Ref	Ref	0.301	
		6 months	10.79 (2.34)	10.67 (9.21, 12.38)	-0.12 (2.18)	2.4 (20.3)	9.66 (1.55)	9.60 (8.62, 10.61)	-0.85 (1.49)	-6.8 (13.2)	0.249	0.203
	OEA/AEA	Baseline	13.28 (1.92)	13.21 (12.07, 14.44)	Ref	Ref	14.74 (3.27)	15.00 (12.46, 16.33)	Ref	Ref	**0.035**	
		6 months	13.35 (1.91)	12.89 (12.04, 14.77)	0.08 (1.27)	1.2 (9.5)	15.60 (3.31)	15.42 (13.08, 17.97)	0.87 (1.52)	6.7 (11.1)	**0.001**	0.112
	OEA/PEA	Baseline	0.57 (0.07)	0.56 (0.52, 0.61)	Ref	Ref	0.58 (0.06)	0.58 (0.54, 0.61)	Ref	Ref	0.495	
		6 months	0.58 (0.08)	0.57 (0.53, 0.63)	0.01 (0.05)	2.1 (8.0)	0.61 (0.06)	0.61 (0.57, 0.67)	0.03 (0.05)	5.7 (9.9)	0.127	0.241
	PEA (nM)	Baseline	19.10 (4.33)	18.80 (16.37, 22.08)	Ref	Ref	18.11 (2.78)	16.86 (15.87, 20.22)	Ref	Ref	0.224	
		6 months	18.70 (3.68)	18.45 (16.13, 20.87)	-0.44 (3.15)	0.0 (17.3)	15.86 (2.04)	16.19 (14.63, 17.52)	-2.26 (2.16)	-11.6 (10.8)	**0.013**	**0.025**
	PEA/AEA	Baseline	23.69 (3.61)	23.32 (21.37, 26.54)	Ref	Ref	25.78 (7.23)	24.46 (20.08, 28.76)	Ref	Ref	0.274	
		6 months	23.26 (3.32)	23.17 (21.28, 24.80)	-0.30 (2.92)	-0.3 (13.2)	25.76 (5.73)	25.81 (22.93, 29.37)	-0.03 (4.44)	2.0 (15.6)	0.042	0.549
	POEA (nM)	Baseline	2.19 (1.00)	2.20 (1.45, 2.89)	Ref	Ref	1.88 (0.52)	1.81 (1.60, 2.20)	Ref	Ref	0.156	
		6 months	2.11 (0.86)	2.04 (1.45, 2.51)	-0.12 (0.74)	5.4 (42.2)	1.61 (0.60)	1.66 (1.09, 1.86)	-0.26 (0.52)	-12.8 (25.5)	**0.028**	0.205
	SEA (nM)	Baseline	5.01 (1.07)	4.85 (4.26, 5.72)	Ref	Ref	4.99 (1.23)	4.70 (4.09, 5.81)	Ref	Ref	0.396	
		6 months	4.90 (1.01)	4.76 (4.15, 5.36)	-0.07 (0.78)	0.2 (16.7)	4.21 (0.69)	4.33 (3.55, 4.76)	-0.79 (0.84)	-13.1 (15.6)	**0.043**	**0.011**
Intervention factors	MedDiet adherence	Baseline	8.0 (2.1)	8.0 (6.0, 9.0)	Ref		7.5 (3.2)	7.0 (5.0, 11.0)	Ref		0.474	
		6 months	10.9 (2.8)	11.0 (9.0, 13.0)	2.9 (2.5)		13.3 (1.4)	13.0 (13.0, 15.0)	5.8 (3.7)		**0.004**	**0.001**
	Physical activity (METs x min/week)	Baseline	2153.5 (1728.7)	1667.8 (881.1, 2972.0)	Ref		2662.4 (2686.5)	1608.4 (1202.8, 3104.9)	Ref		0.829	
		6 months	2591.9 (2009.6)	2028.0 (1279.7, 3304.2)	438.5 (1913.1)		3967.4 (1777.3)	3449.9 (3095.6, 5132.9)	1305.0 (2236.9)		0.147	0.099
	Total energy intake (Kcal/day)	Baseline	2168.3 (495.5)	2112.1 (1886.0, 2401.0)	Ref		2172.7 (365.3)	2194.4 (1979.8, 2408.7)	Ref		0.341	
		6 months	2177.2 (375.2)	2176.4 (1958.5, 2425.8)	8.9 (458.5)		2236.9 (244.1)	2307.4 (2067.5, 2371.0)	64.2 (405.3)		0.241	0.774

Positive/negative values for 6 months change indicate increase and decrease, respectively, compared to the baseline value.
P-values of cross-sectional differences were obtained from the analysis of variance (ANOVA) from multivariable-adjusted linear models. P-values of differences in the rate of change were obtained from linear mixed effects models. All the analyses were adjusted by intervention group, age, diagnostic of type 2 diabetes and baseline body weight. Dependent variables were normalized before estimating p-values, using ordered quantile normalization (ORQ) transformation.
N=number. 95%CI= 95% confidence intervals
Bold values denote statistical significance at P<0.05.

Supplementary Table 8. Description of 3-year changes in endocannabinoids and intervention factors according to the achievement of 5% weight loss after 3 years (respondents/non-respondents)

Sex	Factor	Compound		NON-RESPONDENTS (*do not achieve 5% weight reductions after 3 years*) *[N=22 men and N=23 women]* Missing [N (%)]	Mean (SD)	Median (Q1, Q3)	Mean change (SD)	% mean change	RESPONDENTS (*achieve 5% weight reductions after 3 years*) *[N=26 men and N=34 women]* Missing [N (%)]	Mean (SD)	Median (Q1, Q3)	Mean change (SD)	% mean change	Between-group differences: At each time point	In change over time
Men [N=48]	eCBs	AEA (nM)	Baseline	0 (0.0)	0.76 (0.22)	0.79 (0.53, 0.91)	Ref	Ref	0 (0.0)	0.71 (0.16)	0.69 (0.60, 0.82)	Ref	Ref	0.789	
			3 years	7 (35.0)	0.79 (0.26)	0.71 (0.66, 0.89)	0.06 (0.21)	12.4 (31.0)	2 (7.7)	0.79 (0.26)	0.71 (0.60, 0.86)	0.07 (0.25)	12.8 (45.7)	0.795	0.815
		2-AG (nM)	Baseline	0 (0.0)	6.44 (5.15)	4.64 (3.56, 6.57)	Ref	Ref	0 (0.0)	4.01 (3.34)	3.05 (2.63, 4.49)	Ref	Ref	**0.002**	
			3 years	7 (35.0)	3.76 (1.05)	3.47 (3.36, 4.28)	-1.25 (1.76)	-19.7 (26.1)	2 (7.7)	3.48 (1.97)	2.74 (2.02, 4.60)	-0.73 (3.33)	-3.9 (54.5)	0.242	0.297
		DEA (nM)	Baseline	0 (0.0)	0.20 (0.06)	0.19 (0.16, 0.24)	Ref	Ref	0 (0.0)	0.18 (0.05)	0.18 (0.14, 0.21)	Ref	Ref	0.560	
			3 years	7 (35.0)	0.19 (0.06)	0.19 (0.16, 0.19)	0.00 (0.06)	1.6 (25.1)	2 (7.7)	0.19 (0.06)	0.19 (0.16, 0.23)	0.00 (0.04)	4.0 (21.2)	0.529	0.619
		DGLEA (nM)	Baseline	0 (0.0)	0.16 (0.03)	0.16 (0.13, 0.18)	Ref	Ref	0 (0.0)	0.13 (0.04)	0.13 (0.10, 0.15)	Ref	Ref	0.294	
			3 years	7 (35.0)	0.15 (0.03)	0.15 (0.13, 0.16)	0.00 (0.04)	2.4 (25.2)	2 (7.7)	0.14 (0.05)	0.13 (0.11, 0.16)	0.00 (0.04)	5.3 (32.3)	0.603	0.525
		DHEA (nM)	Baseline	0 (0.0)	1.24 (0.45)	1.25 (0.94, 1.48)	Ref	Ref	0 (0.0)	1.06 (0.36)	0.97 (0.79, 1.27)	Ref	Ref	0.086	
			3 years	7 (35.0)	1.15 (0.35)	1.10 (0.94, 1.39)	-0.03 (0.25)	-0.9 (20.7)	2 (7.7)	1.12 (0.35)	1.04 (0.87, 1.36)	0.05 (0.32)	9.5 (33.0)	0.802	0.322
		DHEA/AEA	Baseline	0 (0.0)	1.71 (0.63)	1.67 (1.21, 1.99)	Ref	Ref	0 (0.0)	1.56 (0.61)	1.40 (1.11, 1.93)	Ref	Ref	**0.026**	
			3 years	7 (35.0)	1.56 (0.60)	1.45 (1.08, 1.71)	-0.17 (0.48)	-6.8 (26.3)	2 (7.7)	1.52 (0.54)	1.50 (1.15, 1.73)	-0.04 (0.43)	1.3 (25.4)	0.427	0.355
		LEA (nM)	Baseline	0 (0.0)	2.31 (0.67)	2.34 (1.74, 2.67)	Ref	Ref	0 (0.0)	1.97 (0.45)	1.92 (1.66, 2.29)	Ref	Ref	0.400	
			3 years	7 (35.0)	2.22 (0.51)	2.18 (2.01, 2.50)	0.05 (0.48)	5.8 (25.2)	2 (7.7)	2.15 (0.62)	2.07 (1.76, 2.52)	0.17 (0.54)	10.2 (29.0)	0.779	0.492
		OEA (nM)	Baseline	0 (0.0)	10.43 (2.51)	10.18 (9.21, 12.56)	Ref	Ref	0 (0.0)	9.50 (1.78)	9.62 (8.15, 10.62)	Ref	Ref	0.679	
			3 years	7 (35.0)	10.72 (2.43)	10.65 (9.61, 11.42)	0.56 (2.31)	10.2 (28.3)	2 (7.7)	10.65 (2.65)	10.06 (8.56, 11.31)	1.12 (2.80)	15.0 (36.9)	0.840	0.464
		OEA/AEA	Baseline	0 (0.0)	14.17 (2.71)	13.69 (12.91, 15.97)	Ref	Ref	0 (0.0)	13.69 (1.82)	13.41 (12.42, 14.89)	Ref	Ref	0.163	
			3 years	7 (35.0)	14.21 (2.77)	13.40 (12.44, 15.93)	-0.14 (2.14)	-0.1 (13.1)	2 (7.7)	14.12 (2.86)	13.35 (12.13, 16.77)	0.65 (2.16)	4.7 (15.7)	0.103	0.493
		OEA/PEA	Baseline	0 (0.0)	0.56 (0.09)	0.56 (0.49, 0.62)	Ref	Ref	0 (0.0)	0.55 (0.06)	0.56 (0.51, 0.60)	Ref	Ref	0.940	
			3 years	7 (35.0)	0.59 (0.09)	0.59 (0.55, 0.65)	0.02 (0.10)	5.1 (19.9)	2 (7.7)	0.62 (0.06)	0.62 (0.58, 0.65)	0.07 (0.07)	13.4 (14.8)	0.418	0.094
		PEA (nM)	Baseline	0 (0.0)	18.82 (4.01)	19.13 (15.44, 20.53)	Ref	Ref	0 (0.0)	17.36 (3.16)	16.88 (15.38, 18.75)	Ref	Ref	0.554	
			3 years	7 (35.0)	18.36 (3.87)	18.50 (15.56, 20.33)	0.66 (4.16)	6.2 (24.5)	2 (7.7)	17.25 (3.77)	16.32 (14.63, 18.54)	-0.21 (3.66)	0.4 (20.5)	0.822	0.784
		PEA/AEA	Baseline	0 (0.0)	25.72 (4.63)	25.26 (22.84, 27.92)	Ref	Ref	0 (0.0)	25.02 (3.20)	24.62 (23.40, 26.43)	Ref	Ref	0.321	
			3 years	7 (35.0)	24.52 (5.51)	23.89 (20.28, 25.66)	-0.85 (3.45)	-3.4 (12.6)	2 (7.7)	22.92 (4.03)	22.29 (20.29, 26.73)	-1.79 (5.03)	-6.2 (18.1)	0.083	0.588
		POEA (nM)	Baseline	0 (0.0)	1.64 (0.77)	1.62 (1.08, 2.22)	Ref	Ref	0 (0.0)	1.56 (0.66)	1.36 (1.10, 1.90)	Ref	Ref	0.346	
			3 years	7 (35.0)	1.41 (0.59)	1.34 (1.07, 1.54)	-0.04 (0.44)	11.1 (52.0)	2 (7.7)	1.66 (1.22)	1.48 (1.01, 1.63)	0.08 (0.96)	6.0 (44.4)	0.277	0.943
		SEA (nM)	Baseline	0 (0.0)	4.71 (0.85)	4.65 (4.17, 5.22)	Ref	Ref	0 (0.0)	4.24 (0.87)	4.18 (3.62, 4.76)	Ref	Ref	0.176	
			3 years	7 (35.0)	4.62 (0.72)	4.61 (4.34, 5.27)	0.01 (0.72)	1.7 (15.5)	2 (7.7)	4.20 (0.86)	4.12 (3.49, 4.58)	-0.10 (0.90)	-0.4 (19.1)	0.261	0.763
	Interven-tion factors	MedDiet adherence	Baseline	0 (0.0)	7.0 (2.9)	6.0 (5.0, 9.2)	Ref		0 (0.0)	6.6 (2.6)	6.5 (4.2, 8.0)	Ref		0.141	
			3 years	6 (30.0)	9.1 (2.6)	9.5 (7.0, 10.0)	1.9 (2.1)		0 (0.0)	11.5 (2.9)	12.0 (9.2, 14.0)	5.0 (2.7)		0.702	**0.002**
		Physical activity (METs x min/week)	Baseline	0 (0.0)	2376.0 (1996.9)	1678.3 (720.3, 3496.5)	Ref		0 (0.0)	3029.0 (2640.3)	2276.2 (961.5, 4993.0)	Ref		0.188	
			3 years	6 (30.0)	2206.1 (1657.3)	1686.7 (1311.9, 3003.5)	-240.8 (2927.7)		0 (0.0)	3896.0 (2312.8)	3723.8 (2360.1, 5337.4)	867.0 (3167.6)		0.292	0.441
		Total energy intake (Kcal/day)	Baseline	0 (0.0)	2467.3 (525.9)	2344.9 (2039.6, 2778.0)	Ref		0 (0.0)	2550.3 (858.2)	2558.4 (1978.3, 2917.4)	Ref		0.561	
			3 years	6 (30.0)	2400.4 (433.3)	2315.9 (2086.8, 2570.4)	23.6 (538.5)		0 (0.0)	2092.4 (329.0)	2067.7 (1963.0, 2348.6)	-457.9 (812.8)		0.155	0.080
Women [N=57]	eCBs	AEA (nM)	Baseline	0 (0.0)	0.79 (0.25)	0.71 (0.61, 0.92)	Ref	Ref	0 (0.0)	0.84 (0.27)	0.80 (0.69, 0.98)	Ref	Ref	0.526	
			3 years	3 (14.3)	0.89 (0.27)	0.85 (0.68, 1.02)	0.13 (0.21)	20.8 (32.2)	3 (8.8)	0.85 (0.23)	0.83 (0.69, 0.95)	0.01 (0.34)	9.3 (40.8)	0.222	0.254
		2-AG (nM)	Baseline	0 (0.0)	4.65 (1.95)	3.83 (3.37, 5.01)	Ref	Ref	0 (0.0)	4.60 (2.09)	3.76 (3.38, 5.72)	Ref	Ref	0.564	
			3 years	3 (14.3)	4.21 (1.49)	4.13 (3.43, 5.10)	-0.51 (2.19)	-0.4 (49.8)	3 (8.8)	4.15 (1.70)	3.73 (2.91, 5.30)	-0.52 (2.47)	0.5 (47.6)	0.280	0.966
		DEA (nM)	Baseline	0 (0.0)	0.17 (0.04)	0.16 (0.14, 0.18)	Ref	Ref	0 (0.0)	0.19 (0.04)	0.18 (0.17, 0.21)	Ref	Ref	0.150	
			3 years	3 (14.3)	0.18 (0.05)	0.17 (0.15, 0.20)	0.01 (0.02)	8.4 (15.9)	3 (8.8)	0.18 (0.05)	0.18 (0.15, 0.20)	0.00 (0.05)	1.0 (29.1)	0.410	0.121
		DGLEA (nM)	Baseline	0 (0.0)	0.18 (0.06)	0.17 (0.15, 0.20)	Ref	Ref	0 (0.0)	0.18 (0.06)	0.17 (0.14, 0.21)	Ref	Ref	0.726	
			3 years	3 (14.3)	0.19 (0.06)	0.17 (0.16, 0.24)	0.02 (0.04)	15.1 (30.1)	3 (8.8)	0.18 (0.03)	0.18 (0.16, 0.20)	0.00 (0.06)	6.1 (32.3)	0.373	0.467
		DHEA (nM)	Baseline	0 (0.0)	1.31 (0.44)	1.28 (0.93, 1.61)	Ref	Ref	0 (0.0)	1.33 (0.44)	1.28 (1.06, 1.45)	Ref	Ref	0.542	
			3 years	3 (14.3)	1.34 (0.34)	1.31 (1.10, 1.66)	0.11 (0.34)	13.9 (33.3)	3 (8.8)	1.37 (0.43)	1.25 (1.09, 1.52)	0.00 (0.37)	3.4 (26.5)	0.560	0.459
		DHEA/AEA	Baseline	0 (0.0)	1.72 (0.48)	1.60 (1.30, 1.91)	Ref	Ref	0 (0.0)	1.66 (0.48)	1.62 (1.35, 1.95)	Ref	Ref	0.787	
			3 years	3 (14.3)	1.60 (0.44)	1.76 (1.35, 1.92)	-0.08 (0.41)	-3.0 (23.6)	3 (8.8)	1.65 (0.46)	1.66 (1.29, 1.95)	-0.04 (0.44)	1.4 (29.9)	0.078	0.537
		LEA (nM)	Baseline	0 (0.0)	2.08 (0.59)	2.00 (1.69, 2.33)	Ref	Ref	0 (0.0)	2.38 (0.76)	2.27 (1.79, 2.92)	Ref	Ref	0.292	
			3 years	3 (14.3)	2.33 (0.76)	2.14 (1.84, 2.85)	0.32 (0.63)	19.2 (33.2)	3 (8.8)	2.38 (0.65)	2.31 (1.94, 2.86)	-0.06 (0.79)	3.2 (30.0)	0.695	0.143
		OEA (nM)	Baseline	0 (0.0)	10.25 (2.48)	10.29 (9.30, 11.69)	Ref	Ref	0 (0.0)	11.10 (2.72)	10.82 (9.32, 13.02)	Ref	Ref	0.281	

		3 years	3 (14.3)	10.89 (2.41)	11.09 (9.16, 12.30)	0.98 (1.95)	12.7 (24.3)	3 (8.8)	12.07 (3.03)	11.47 (9.76, 13.76)	0.76 (3.16)	11.0 (31.1)	0.700	0.853
	OEA/AEA	Baseline	0 (0.0)	13.38 (2.02)	13.20 (12.00, 14.46)	Ref	Ref	0 (0.0)	13.69 (2.60)	13.27 (12.46, 14.93)	Ref	Ref	0.270	
		3 years	3 (14.3)	12.68 (2.24)	12.60 (10.79, 14.01)	-0.68 (1.69)	-4.6 (12.2)	3 (8.8)	14.44 (2.79)	14.56 (12.57, 16.05)	0.58 (2.13)	5.2 (16.5)	**0.019**	**0.024**
	OEA/PEA	Baseline	0 (0.0)	0.56 (0.07)	0.55 (0.52, 0.60)	Ref	Ref	0 (0.0)	0.57 (0.07)	0.58 (0.54, 0.61)	Ref	Ref	0.412	
		3 years	3 (14.3)	0.56 (0.07)	0.55 (0.51, 0.64)	0.00 (0.04)	1.0 (7.7)	3 (8.8)	0.63 (0.10)	0.63 (0.55, 0.70)	0.05 (0.07)	9.5 (12.4)	0.189	**0.007**
	PEA (nM)	Baseline	0 (0.0)	18.27 (4.00)	17.08 (16.50, 21.19)	Ref	Ref	0 (0.0)	19.26 (3.96)	19.62 (15.96, 21.96)	Ref	Ref	0.302	
		3 years	3 (14.3)	19.37 (3.82)	18.23 (17.14, 21.34)	1.65 (2.75)	11.0 (18.4)	3 (8.8)	19.08 (3.57)	19.01 (15.58, 21.69)	-0.42 (4.08)	0.6 (21.6)	0.611	0.114
	PEA/AEA	Baseline	0 (0.0)	24.04 (3.37)	23.24 (21.69, 26.77)	Ref	Ref	0 (0.0)	24.16 (5.50)	23.34 (20.22, 26.83)	Ref	Ref	0.764	
		3 years	3 (14.3)	22.68 (3.52)	21.74 (20.00, 25.73)	-1.41 (3.38)	-5.1 (13.5)	3 (8.8)	23.02 (3.73)	22.99 (20.90, 25.82)	-1.31 (5.33)	-2.2 (22.0)	0.154	0.595
	POEA (nM)	Baseline	0 (0.0)	2.06 (0.85)	1.93 (1.50, 2.72)	Ref	Ref	0 (0.0)	2.16 (0.94)	1.90 (1.60, 2.62)	Ref	Ref	0.347	
		3 years	3 (14.3)	2.01 (0.59)	2.08 (1.68, 2.48)	0.06 (0.63)	15.7 (46.7)	3 (8.8)	2.17 (1.12)	2.00 (1.41, 2.68)	-0.02 (0.97)	6.4 (43.3)	0.718	0.493
	SEA (nM)	Baseline	0 (0.0)	4.89 (1.07)	4.62 (4.25, 5.22)	Ref	Ref	0 (0.0)	5.07 (1.10)	5.08 (4.27, 5.84)	Ref	Ref	0.639	
		3 years	3 (14.3)	5.29 (1.42)	4.96 (4.60, 5.75)	0.54 (0.85)	11.0 (17.2)	3 (8.8)	5.03 (0.89)	5.37 (4.26, 5.63)	-0.13 (1.00)	-0.2 (18.9)	0.448	0.091
Interven-tion factors	MedDiet adherence	Baseline	0 (0.0)	7.7 (1.8)	8.0 (6.0, 9.0)	Ref		0 (0.0)	7.7 (2.5)	8.0 (6.0, 9.8)	Ref		0.923	
		3 years	3 (14.3)	9.2 (2.7)	9.0 (8.0, 11.0)	1.4 (2.9)		1 (2.9)	11.0 (2.4)	11.0 (9.0, 12.0)	3.1 (2.8)		**0.008**	**0.022**
	Physical activity (METs x min/week)	Baseline	0 (0.0)	1925.2 (2006.0)	1258.7 (559.4, 1958.0)	Ref		0 (0.0)	2512.4 (1992.7)	1682.0 (1235.2, 3117.5)	Ref		0.105	
		3 years	3 (14.3)	2094.5 (2332.1)	1223.8 (842.2, 2587.4)	169.4 (1970.9)		1 (2.9)	2676.8 (1848.9)	2255.0 (1447.6, 3608.4)	139.1 (1890.2)		0.686	0.964
	Total energy intake (Kcal/day)	Baseline	0 (0.0)	2248.6 (564.8)	2195.3 (1964.6, 2488.9)	Ref		0 (0.0)	2130.4 (380.9)	2137.1 (1902.9, 2374.5)	Ref		0.503	
		3 years	3 (14.3)	2003.5 (405.1)	2014.4 (1797.7, 2143.9)	-259.9 (689.6)		2 (5.9)	2066.1 (375.0)	2077.6 (1850.8, 2213.8)	-92.3 (446.7)		0.141	0.288

Positive/negative values for 3 years change indicate increase and decrease, respectively, compared to the baseline value.
P-values of cross-sectional differences were obtained from the analysis of variance (ANOVA) from multivariable-adjusted linear models. P-values of differences in the rate of change were obtained from linear mixed effects models. All the analyses were adjusted by intervention group, age, diagnostic of type 2 diabetes and baseline body weight. Dependent variables were normalized before estimating p-values, using ordered quantile normalization (ORQ) transformation.
N=number. 95%CI= 95% confidence intervals
Bold values denote statistical significance at $P<0.05$.

Supplementary Table 9. ANOVA table of Generalized Additive Model (GAM) results for the association between relative changes in eCBs, NAEs and their ratios, and the achievement of successful weight reductions, stratified by sex

	Exposure:	Outcome: 8% weight reductions after 6 months				Outcome: 5% weight reductions after 3 years			
**Sex**	**% change**	**edf**	**Ref. df**	**Chi.sq**	**P-value**	**edf**	**Ref.df**	**Chi.sq**	**p.value**
Women [N=57]	2-AG	3.91	4.03	0.00	1.000	1.00	1.00	0.02	0.894
	AEA	3.89	4.65	7.15	0.194	5.85	6.34	1.58	0.967
	OEA	1.85	2.34	2.32	0.372	1.00	1.00	4.66	**0.031**
	DHEA	4.27	5.11	5.68	0.351	8.14	8.32	0.93	0.999
	PEA	1.32	1.58	3.35	0.156	1.00	1.00	0.92	0.336
	OEA/AEA	1.00	1.00	5.39	**0.020**	1.00	1.00	4.22	**0.040**
	DHEA/AEA	1.00	1.00	0.63	0.427	2.86	3.56	5.83	0.168
	PEA/AEA	3.04	3.80	3.87	0.443	1.00	1.00	2.22	0.136
	OEA/AEA	1.00	1.00	0.15	0.695	1.00	1.00	2.26	0.132
	POEA	1.00	1.00	1.31	0.252	1.00	1.00	6.11	**0.013**
	DEA	3.77	4.08	1.00	0.917	3.04	3.37	1.09	0.860
	DGLEA	5.53	5.64	0.35	0.999	5.24	5.32	0.01	1.000
	SEA	1.00	1.00	0.06	0.802	1.00	1.00	6.13	**0.013**
	LEA	1.75	2.20	2.89	0.267	4.14	4.23	0.00	1.000
Men [N=48]	2-AG	7.32	7.34	0.90	0.998	1.00	1.00	1.52	0.217
	AEA	3.72	4.62	2.49	0.654	3.47	4.24	5.73	0.239
	OEA	1.00	1.00	1.58	0.209	1.00	1.00	0.01	0.922
	DHEA	1.00	1.00	2.02	0.155	1.00	1.00	1.41	0.235
	PEA	6.12	7.11	5.02	0.686	7.58	7.93	1.14	0.997
	OEA/AEA	3.89	4.76	5.69	0.280	2.94	3.21	0.20	0.989
	DHEA/AEA	1.00	1.00	1.25	0.264	1.00	1.00	0.42	0.518
	PEA/AEA	7.10	7.74	6.47	0.579	5.56	5.93	0.80	0.990
	OEA/AEA	1.00	1.00	5.95	**0.015**	1.53	1.91	1.02	0.511
	POEA	6.16	7.12	5.59	0.619	1.00	1.00	0.71	0.400
	DEA	1.00	1.00	1.65	0.199	4.91	5.38	0.57	0.994
	DGLEA	1.00	1.00	2.65	0.104	1.00	1.00	0.81	0.368
	SEA	3.64	4.51	6.65	0.217	3.74	4.70	3.68	0.615
	LEA	2.42	3.07	4.50	0.220	1.00	1.00	0.00	1.000

Models were adjusted by baseline body weight, age, intervention group and diagnostic of diabetes.

The effective degrees of freedom (edf) estimated from generalized additive models were used as a proxy for the degree of non-linearity in exposure-response relationships. The edf would equal 1 if the model penalized the smooth term to a simple linear relationship. An edf < 1 and ≤ 2 represents a weakly non-linear relationship, and (c) an edf > 2 indicates a highly non-linear relationship. Ref.df= reference degrees of freedom used in computing test statistic and the p-values. Chi.sq= test statistics for assessing the significance of model smooth terms. P-values indicate the significance of model smooth terms.

Bold values denote statistical significance at P<0.05.
